# Selective Boryl‐Anion Migration in a Vinyl sp^2^−sp^3^ Diborane Induced by Soft Borane Lewis Acids

**DOI:** 10.1002/anie.201808216

**Published:** 2018-09-10

**Authors:** Valerio Fasano, Jessica Cid, Richard J. Procter, Emily Ross, Michael J. Ingleson

**Affiliations:** ^1^ School of Chemistry University of Manchester Oxford Road Manchester M13 9PL UK

**Keywords:** boranes, borylation, Grignard reagents, Lewis acids, 1,2-migration

## Abstract

An intramolecular 1,2‐boryl‐anion migration from boron to carbon has been achieved by selective activation of the π system in [(vinyl)B_2_Pin_2_)]^−^ using “soft” BR_3_ electrophiles (BR_3_=BPh_3_ or 9‐aryl‐BBN). The soft character is key to ensure 1,2‐migration proceeds instead of oxygen coordination/B−O activation. The BR_3_‐induced 1,2‐boryl‐anion migration represents a triple borylation of a vinyl Grignard reagent using only B_2_Pin_2_ and BR_3_ and forms differentially protected 1,1,2‐triborylated alkanes. Notably, by increasing the steric bulk at the β position of the vinyl Grignard reagent used to activate B_2_Pin_2_, 1,2‐boryl‐anion migration can be suppressed in favor of intermolecular {BPin}^−^ transfer to BPh_3_, thus enabling simple access to unsymmetrical sp^2^−sp^3^ diboranes.

The coordination of a Lewis base (LB) to diborane(4) compounds, such as B_2_Pin_2_ (**1**), generates an sp^2^−sp^3^ diborane in which the boron–boron bond is polarised,^1^ which imparts nucleophilic character to the sp^2^ boron atom, thereby enabling the mild generation of a functional equivalent of {BPin}^−^.^1^, ^2^ This strategy has become a powerful transition‐metal‐free method to borylate organic substrates and generate desirable organoboronate esters. Alkoxides or N‐heterocyclic carbenes (NHCs) are the typical LBs employed in the activation of **1**,^1^, ^2^, ^3^ with the use of carbanions (R^−^) having much less precedent,^4^, ^5^, ^6^, ^7^, ^8^, ^9^ despite the ability of carbanions to generate a more nucleophilic {BPin} moiety owing to their greater basicity relative to alkoxides and NHCs. Among the limited examples in this area, recent studies have shown that complex **A** synthesised from **1** and *n*Bu−MgL (L=β‐diketiminato) transfers a boryl anion to boranes to form new unsymmetrical sp^2^−sp^3^ diboranes (Scheme 1 a).^10^ Indeed, transfer of a boryl nucleophile to an external electrophile is the dominant reactivity pathway reported for B_2_Pin_2_ activated by simple carbanions.^10^ It is important to extend the chemistry of [(R)B_2_Pin_2_)]^−^ to enable new routes to highly functionalized organoboronates to be discovered. Such routes will be particularly desirable if readily accessible starting materials (e.g. RMgX/B_2_pin_2_) can be used.

**Scheme 1:**
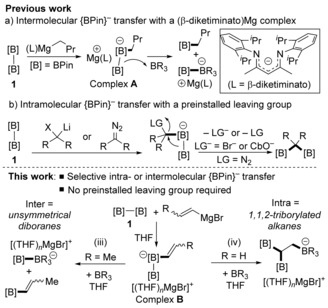
Top: Previous studies on intermolecular/intramolecular {BPin}^−^ transfer in carbanion‐activated B_2_Pin_2_. Bottom: Selective boryl‐anion migration in vinyl sp^2^−sp^3^ diboranes as induced by soft borane Lewis acids.

Prior to this study, 1,2‐boryl‐anion migration from boron to carbon in [(R)B_2_Pin_2_]^−^ species had been limited to the use of functionalized “R^−^” equivalents. For example, coordination of a carbanion containing a Br or OCb group (or a diazoalkane) to **1** led to loss of [OCb]^−^ or [Br]^−^ (or N_2_) and the formation of 1,1‐diborylalkanes (Scheme 1 b).^11^, ^12^, ^13^, ^14^, ^15^, ^16^, ^17^ We hypothesised that an alternative route to induce intramolecular 1,2‐boryl‐anion migration would be the activation of an unsaturated R^−^ group (e.g. −CH=CH_2_) in [(R)B_2_Pin_2_]^−^ by a borane Lewis acid. This approach is attractive as it avoids prefunctionalization of the carbanion activator. It is conceptually related to the Zweifel reaction,^18^ but the use of borane Lewis acids and {BPin}^−^ as the migrating group will lead to differentially functionalised 1,1,2‐triborylated alkanes in one step. Related 1,1‐diborylated alkanes have emerged as highly versatile reagents used in selective C−C bond formation by the Suzuki–Miyaura coupling reaction or by deprotonation/deborylation of the diborylated carbon atom.^19^, ^20^, ^21^, ^22^

The selective (for intramolecular 1,2‐boryl migration) activation of [(vinyl)B_2_Pin_2_]^−^ (complex **B**, Scheme 1, bottom), requires judicious choice of the borane, BR_3_, as a range of outcomes are feasible, including: i) vinyl‐anion transfer from **B** to BR_3_; ii) binding of BR_3_ to an oxygen atom in **B** and subsequent C−O or B−O cleavage; iii) {BPin}^−^ anion transfer from **B** to BR_3_; iv) BR_3_ activation of the vinyl π system and intramolecular {BPin}^−^ transfer. While (i) and (ii) are undesirable, pathway (iii) would be an attractive route to unsymmetrical diboranes using commercial Grignard reagents as activators. Equally notable and our primary focus, intramolecular 1,2‐boryl migration (pathway (iv)) would be a new and simple route to 1,1,2‐triborylated alkanes.

Herein, we report that intramolecular 1,2‐boryl migration in sp^2^−sp^3^ diboranes does not require preinstalled leaving groups in the carbanion. Instead, the formation of [(vinyl)B_2_Pin_2_]^−^, followed by selective activation of the π system by certain boranes, forms differentially functionalised (at boron) 1,1,2‐triborylated alkanes. The use of a β‐methyl vinyl Grignard reagent changes the reaction outcome to intermolecular {BPin}^−^ transfer to BR_3_, generating an unsymmetrical diborane from simple starting materials.

We started our investigation by probing the accessibility of the simplest vinyl adduct of **1**, [(CH_2_=CH)B_2_Pin_2_]^−^ (**[2]^−^**), which could be generated as the major product by the addition of 1 equivalent of commercial vinyl magnesium bromide to **1** in THF at −78 °C (Scheme 2, left). The successful formation of **[2]^−^** was indicated by ^11^B NMR spectroscopy, which showed two new resonances: one at 37.3 ppm (three‐coordinate boron) and the other at 4.8 ppm (four‐coordinate boron), analogous to the spectrum reported for [(Ph)B_2_Pin_2_]^−^ (39.2 and 4.0 ppm, respectively).^6^ Since B(C_6_F_5_)_3_ can activate alkenes and alkynes even in the presence of certain oxo functionalities, the ability of B(C_6_F_5_)_3_ to trigger the 1,2‐boryl migration was explored.^23^ The addition of B(C_6_F_5_)_3_ (1 equiv) to **[2]^−^** (at −78 °C) led after 2 h to a single new ^11^B resonance at −3.2 ppm, consistent with an [RO−B(C_6_F_5_)_3_]^−^ species (in contrast, [alkyl−B(C_6_F_5_)_3_]^−^ anions have a ^11^B resonance at ca. −15 ppm). The ^19^F NMR spectrum confirmed [RO−B(C_6_F_5_)_3_]^−^ formation, with ESIMS analysis supporting the formation of an [RO−B(C_6_F_5_)_3_]^−^ species derived from ring opening of one BPin moiety in **[2]^−^**. With two additional ^11^B resonances observed at 48.0 and 29.2 ppm, we tentatively assign the product as derived from B(C_6_F_5_)_3_ activation of pinacol bound to the four‐coordinate boron atom (Scheme 2, top). This assignment is consistent with reports on BPin moieties in anionic borates undergoing B−O cleavage on addition of electrophiles.^24^

**Scheme 2:**
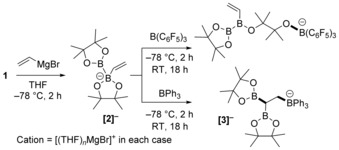
Reaction of **1** with a vinyl Grignard reagent and B(C_6_F_5_)_3_ or BPh_3_.

The oxo‐based reactivity of B(C_6_F_5_)_3_ with **[2]^−^** was attributed to the high electrophilicity and oxophilicity of this borane. Therefore, softer boron electrophiles were explored, in particular BPh_3_, since this borane reacts with complex **A** to generate [PinB−BPh_3_]^−^ with no competitive reactivity at the oxo sites reported (Scheme 1 a).^10^ The addition of BPh_3_ (1 equiv) in THF to **[2][(THF)**_***n***_ **MgBr]** generated in situ (at −78 °C) resulted in the formation of the desired product **[3]^−^** formed by intramolecular {BPin}^−^ transfer (Scheme 2, bottom). Anion **[3]^−^** has diagnostic resonances in the ^11^B NMR spectrum (34.7 ppm for the C−BPin moieties, and −9.5 ppm for [C−BPh_3_]^−^) and in the ^1^H NMR spectrum (broad signal at 0.55 ppm for C*H*(BPin)_2_), with the formulation further confirmed by accurate mass spectrometry. Performing the reaction at −78 °C for 2 h and then room temperature for 18 h resulted in complete consumption of **[2]^−^** to yield **[3]^−^** (71 % in situ conversion) as the major product. When the reaction was repeated on a larger scale, **[3][(THF)_2_MgBr]** was isolated as a white solid in 70 % yield by solvent removal and washing with Et_2_O.

Single crystals of **[3][(THF)_2_MgBr]** were obtained by slow diffusion of pentane into a THF solution (Figure 1). In the solid‐state structure, the cation is chelated by the two pinacolato moieties of **[3]^−^** through oxygen coordination to magnesium, which results in modest elongation of the B−O bonds involving oxygen atoms coordinated to Mg (compare bonds e and f in Figure 1).^25^ Other distances and angles in **[3][(THF)_2_MgBr]** are within the expected values, with C−BPin bond distances shorter than the C−BPh_3_ distance (c and d vs. a in Figure 1). In solution in [D_8_]THF, **[3][(THF)_2_MgBr]** shows two singlets in the ^1^H NMR spectrum at 298 K for the methyl groups of the pinacols, thus indicating the inequivalence of these hydrogen atoms on the NMR timescale owing to chelation to Mg. Cation metathesis using [Me_4_N][Cl] formed the air‐stable product **[3][Me_4_N]**, in which the pinacol methyl groups now exhibit a single resonance in the ^1^H NMR spectrum at 298 K (in THF). The one‐pot triborylation of a vinyl Grignard reagent has not been reported previously to the best of our knowledge.

**Figure 1:**
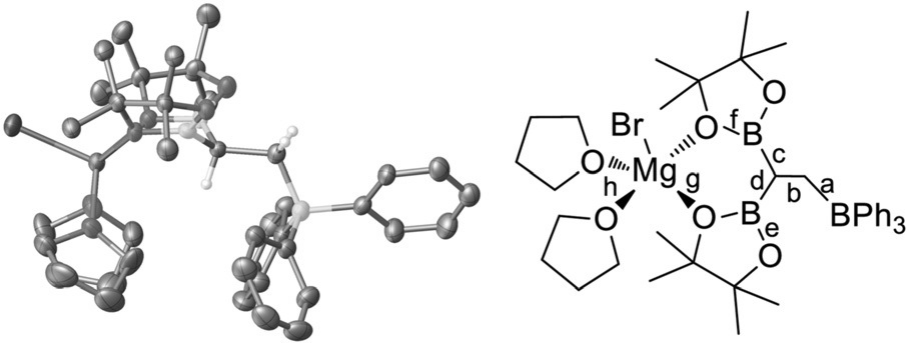
Left: Solid‐state structure of **[3][(THF)_2_MgBr]** with ellipsoids at 50 % probability (some hydrogen atoms omitted for clarity). Right: Molecular structure with selected bonds labelled, distances [Å]: *a*=1.663(9), *b*=1.571(7), *c*=1.545(8), *d*=1.554(8), *e*=1.358(8), *f*=1.417(7), *g*=2.118(3), and *h*=2.066(4).

Regarding the mechanism of formation, the arrangement of boranes in **[3]^−^** excludes the possibility of vinyl transfer from **[2]^−^** to BPh_3_, followed by diboration of the vinyl group in [(CH_2_=CH)BPh_3_]^−^ with B_2_Pin_2_ (or base‐activated B_2_pin_2_), since this reaction pathway would lead to 1,2‐arrangement of the BPin groups and not 1,1.^1^, ^2^ To gain further insight into the reaction mechanism and the disparity between BPh_3_ and B(C_6_F_5_)_3_, we performed DFT calculations at the M06‐2X/6–311G(d,p) level with a solvent polarisable continuum model (PCM, THF). On the basis of the structure of **[3][(THF)_2_MgBr]**, the cation [(THF)_2_MgBr]^+^ was included initially. The formation of the neutral adduct **2′** from **1** and the vinyl Grignard reagent is energetically favoured (Δ*G*_298K_=−9.8 kcal mol^−1^), despite the adverse entropic contribution (Scheme 3). Adduct **2′** showed a slightly elongated B−B bond relative to that of **1** (1.73 and 1.70 Å, respectively), as reported for other sp^2^−sp^3^ diboranes.^1^, ^2^ The addition of BPh_3_ to **2′** to yield the product **[3][(THF)_2_MgBr]** is energetically downhill (Δ*G*_298K_=−42.0 kcal mol^−1^). To gain insight into the disparate borane reactivity (B−O activation vs. π activation), we probed the change in energy upon BR_3_ coordination to the oxygen atom of **2′**. For BPh_3_, this process is energetically uphill (Δ*G*_298K_=5.2 kcal mol^−1^), in agreement with the reduced electrophilicity and oxophilicity of this borane relative to B(C_6_F_5_)_3_. Upon replacement of BPh_3_ with B(C_6_F_5_)_3_ (Scheme 3, bottom), O‐coordination becomes significantly exergonic (Δ*G*_298K_=−8.8 kcal mol^−1^), consistent with the observation of B−O cleavage on mixing **[2]^−^** and B(C_6_F_5_)_3_. Thus, the correct tuning of the oxophilicity/electrophilicity of the borane employed is a key aspect in selectively triggering 1,2‐boryl migration. This feature is further emphasised by replacing B(C_6_F_5_)_3_ with the harder Lewis acid BF_3_, with O‐coordination now becoming much more exergonic (Δ*G*_298K_=−26.4 kcal mol^−1^ relative to **2′** and BF_3_). Attempts to crystallise **[2][(THF)**_***n***_ **MgBr]** were unsuccessful in our hands; thus, owing to the unknown exact nature of the magnesium species coordinated to **[2]^−^**, and to facilitate more detailed computational studies, additional DFT calculations were performed in the absence of the counterion. The calculated HOMO and HOMO‐1 of **[2]^−^** are analogous to those of **2′**, thus indicating that while Mg coordination will effect energies to some extent it does not drastically effect the electronic distribution of the frontier orbitals. The HOMO of **[2]^−^** has polarised σ B−B character (consistent with the observed {BPin} nucleophilic character), as well as some σ B−C(vinyl) and lone‐pair oxygen character (Figure 2, left). The π C=C orbital instead contributes to the HOMO‐1, with the vinyl system almost completely aligned with the B−B bond (B‐B‐C=C 12.10°).

**Figure 2:**
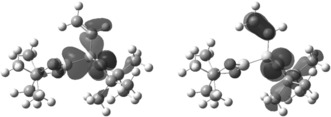
Calculated HOMO and HOMO‐1 of **[2]^−^** (isovalue=0.04). **[2]^−^** and **2′** showed similar geometry (particularly regarding the B‐B‐C=C dihedral angle) and HOMOs; thus, the former is provided and not **[2_B_]^−^**.

**Scheme 3:**
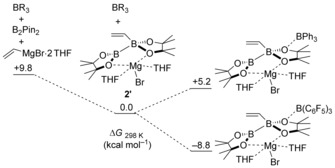
Free‐energy profile for the formation of **2′** and O‐coordination of the latter to the borane (the zero‐energy reference is set as **2′**+BR_3_ in each case).

The potential‐energy surface is flat where complex **[2]^−^** is located, with different local minima obtained by rotation of the vinyl group around the B−C(vinyl) bond. To trigger the intramolecular 1,2‐boryl migration, a correct arrangement of the vinyl moiety relative to the B−B bond is required for the *trans* addition of BPh_3_ and BPin to the C=C bond (Scheme 4). From this arrangement (**[2_B_]^−^**), the reaction proceeds via transition state **TS** with a low free‐energy barrier of 15.2 kcal mol^−1^ at 298 K. In **TS**, the vinyl system is almost perpendicular to the B−B bond (torsional angle B‐B‐C=C 85.96°), with both the B−B and the C=C bonds slightly elongated as compared to **[2_B_]^−^** (1.75 vs. 1.73 Å, and 1.36 vs. 1.33 Å, respectively). Bond‐order analysis of **TS** revealed that the reaction proceeds through an asynchronous concerted mechanism, with the C−BPh_3_ bond formed to a greater extent than the C−BPin bond (0.29 and 0.08, respectively).

**Scheme 4:**
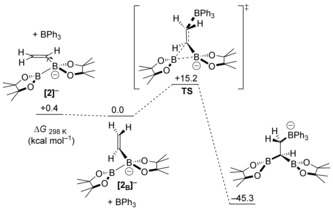
Free‐energy reaction profile for BPh_3_‐induced 1,2‐boryl migration.

Having gained this understanding of the reaction mechanism, we tested other soft boron‐based Lewis acids. The addition of 9‐Ph‐BBN (1 equiv) to **[2]^−^** (at −78 °C) gave the desired product **[4]^−^**, with diagnostic peaks observed in the ^11^B NMR spectrum (34.0 ppm for the −BPin moieties, and −15.3 ppm for [R(Ph)BBN]^−^) and in the ^1^H NMR spectrum (upfield broad signal at 0.24 ppm for C*H*(BPin)_2_), and mass spectrometry confirming the formulation for the anion **[4]^−^** (Scheme 5, top). **[4][(THF)_2_MgBr]** was isolated in 52 % yield (^1^H NMR spectroscopy indicated the coordination of two molecules of THF to [MgBr]^+^). Interestingly, in this case the tetracoordinated boron centre in **[4]^−^** has restricted rotation causing desymmetrization of the bicyclo moiety. Notably, **[4][(THF)_2_MgBr]** could be selectively deborylated by the addition of HNTf_2_ (1.1 equiv), which yielded 9‐Ph‐BBN and (PinB)_2_CHMe as the major products, thus indicating that cleavage of the C−(Ph)BBN bond dominates. In contrast, (PinB)_2_CHMe was formed in low amounts from the addition of HNTf_2_ to **[2]^−^**, with the formation of ethene and **1** dominating (Scheme 5, bottom).

**Scheme 5:**
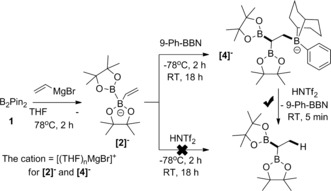
Top: Reaction of **1**, a vinyl Grignard reagent, and 9‐phenyl‐9‐borabicyclo[3.3.1]nonane (9‐Ph‐BBN). Bottom: Synthesis of a 1,1‐diborylethane through protodeboronation of **[4]^−^**; the same product was formed in only low yield by the direct protonation of **[2]^−^**.

These results highlight the importance of using a soft Lewis acid to selectively trigger the 1,2‐boryl migration over other potential pathways. To confirm that the reactivity difference between B(C_6_F_5_)_3_ and BPh_3_ (or 9‐Ph‐BBN) is not due to steric factors (as B(C_6_F_5_)_3_ is significantly bulkier than BPh_3_), we evaluated 9‐mesityl‐BBN and 9‐*o*‐tolyl‐BBN. Whereas the former gave no reaction with **[2]^−^** (presumably owing to the large steric bulk around boron), the addition of *o*‐tolyl‐BBN to **[2]^−^** in THF led to the intramolecular 1,2‐boryl‐anion migration product **[5]^−^**, albeit slower than when using 9‐Ph‐BBN. Importantly, no B−O cleavage products were observed, with the mass balance at this point being unreacted **[2]^−^** and *o*‐tolyl‐BBN. Thus, with bulkier, less Lewis acidic 9‐aryl‐BBN boranes, the 1,2‐boryl migration still proceeds selectively but is slower. This reactivity was further emphasised by adding 9‐*p*‐anisyl‐BBN to **[2]^−^**, upon which the 1,2‐boryl‐anion migration proceeded to form **[6]^−^** but significantly slower owing to the reduced borane Lewis acidity (see the Supporting Information).

With the aim to disfavour the interaction of borane Lewis acids with the vinylic π system and thus switch the selectivity from intra‐ to intermolecular {BPin}^−^ transfer, we explored the effect of increasing steric hindrance at the β‐vinylic carbon atom by using the adduct **[7]^−^**, which was generated in situ by the addition of (*E*/*Z*)‐1‐propenylmagnesium bromide (1 equiv) to **1** in THF at −78 °C. The subsequent addition of BPh_3_ to **[7]^−^** resulted in suppression of 1,2‐boryl migration, with **[8]^−^** detected only in trace amounts (Scheme 6). In this case, [PinB−BPh_3_]^−^ (40 % yield) and (*E*/*Z*)‐1‐propenyl‐BPin were observed as the major new species after 18 h at room temperature, thus confirming the switching of selectivity from intra‐ to intermolecular {BPin}^−^ transfer. This represents a simple route to an unsymmetrical sp^2^−sp^3^ diborane using only commercial reagents.

**Scheme 6:**
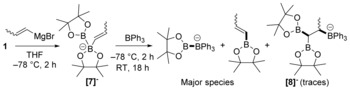
Reaction of **1** with 1‐propenyl Grignard reagent and then BPh_3_. The cation is assigned as [(THF)_*n*_MgBr]^+^ throughout.

In summary, a novel intramolecular 1,2‐boryl‐anion migration has been induced by the addition of soft boranes to vinyl sp^2^−sp^3^ diboranes. Competitive strong oxygen coordination has to be prevented; thus, the softness of the borane is key in providing selective boryl transfer. With BPh_3_ and 9‐Ph‐BBN, intramolecular 1,2‐boryl migration enables the one‐pot synthesis of differentially protected 1,1,2‐triborylated alkanes from simple starting materials. Furthermore, the ability to switch {BPin}^−^ transfer from an intra‐ to an intermolecular process by increasing the steric hindrance in the vinyl group allows access to unsymmetrical sp^2^−sp^3^ diboranes using commercial Grignard reagents and B_2_Pin_2_.

## Conflict of interest

The authors declare no conflict of interest.

## Supporting information

As a service to our authors and readers, this journal provides supporting information supplied by the authors. Such materials are peer reviewed and may be re‐organized for online delivery, but are not copy‐edited or typeset. Technical support issues arising from supporting information (other than missing files) should be addressed to the authors.

Supplementary
