# Supplementary material for: Selective Boryl‐Anion Migration in a Vinyl sp2−sp3 Diborane Induced by Soft Borane Lewis Acids
Source: Angew Chem Int Ed Engl. 2018 Sep 10;57(40):13293–7. doi: 10.1002/anie.201808216 (PMC6221116; doi:10.1002/anie.201808216)
Supplement: Supplementary file 1 — Supplementary [file ANIE-57-13293-s001.pdf]

Angewandte Chemie
Eine Zeitschrift der Gesellschaft Deutscher Chemiker

Supporting Information

Selective Boryl-Anion Migration in a Vinyl sp^2–sp^3 Diborane Induced by Soft Borane Lewis Acids

*Valerio Fasano, Jessica Cid, Richard J. Procter, Emily Ross, and Michael J. Ingleson**

anie_201808216_sm_miscellaneous_information.pdf

Supporting Information

Table of Contents

1. General Remarks S2
2. Formation of [B_2Pin$_2$-vin] (**[2]**$^-$) S2
3. Borane-induced activation of **[2]**$^-$ S4
3.1 Addition of $B(C_6F_5)_3$ S4
3.2 Addition of BPh_3 S6
3.3 Addition of 9-Ph-BBN S13
3.4 Addition of 9-Mesityl-BBN S18
3.5 Addition of 9-*o*-Tolyl-BBN S20
3.6 Addition of 9-*p*-Anisyl-BBN S23
4. Addition of BPh_3 to **[7]**$^-$ S26
5. Addition of $HNTf_2$ to **[4][MgBr(THF)$_2$]** S30
6. Addition of $HNTf_2$ to **[2]**$^-$ S33
7. Computational data S36
8. Crystallographic details of **[3][MgBr(THF)$_2$]** S60
9. References S61

1. General Remarks

Unless otherwise indicated all manipulations were conducted under nitrogen atmosphere. B_2Pin_2 was kindly provided by AllylChem. Vinyl Grignard (1 M in THF), isopropenyl Grignard (0.5 M in THF), (E/Z)-1-propenyl Grignard (0.5 M in THF), BPh_3 (0.25 M in THF) were purchased from commercial sources and used as received unless otherwise stated. $B(C_6F_5)_3$ was dried over Et_3SiH (in a pentane solution), followed by sublimation. 9-Aryl-BBN compounds were synthesized from commercially available 9-methoxy-BBN and ArylMgBr, following the procedure reported in the literature.[1] THF was dried over elemental potassium. NMR spectra were recorded with a Bruker AV-400 spectrometer (400 MHz ^{1}H; 100 MHz ^{13}C; 128 MHz ^{11}B; 376 MHz ^{19}F). ^{1}H NMR chemical shifts are reported in ppm relative to *protio* impurities in the deuterated solvents and ^{13}C NMR chemical shifts using the solvent resonances unless otherwise stated. ^{11}B NMR spectra were referenced to external BF_3:Et_2O, ^{19}F to Cl_3CF). Coupling constants *J* are given in Hertz (Hz), while the multiplicity of the signals are indicated as “s”, “d”, “t”, “q”, “pent”, “sept” or “m” for singlet, doublet, triplet, quartet, pentet, septet or multiplet, respectively. A sealed capillary containing *d*$_6$-DMSO is inserted in the J. Young NMR tube for the locking of the NMR sample. Mass spectra were recorded on a Waters QTOF mass spectrometer.

2. Formation of [B_2Pin_2-vinyl] ([2]$^-$)

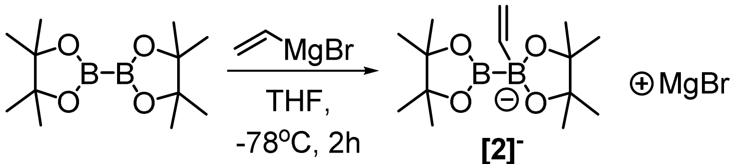

[2]$^-$

In a J. Young NMR tube, B_2Pin_2 (53 mg, 0.200 mmol, 1.0 eq.) was dissolved in dry THF (0.3 mL) and the solution was then cooled down to -78°C. After 5 min, a 1 M solution of vinyl magnesium bromide (200 µL, 0.200 mmol, 1.0 eq.) was added. The solution was kept at -78°C for 2 hours, with the J. Young NMR tube inverted each 30 min. The sample was left warming to room temperature and then was monitored by multi-nuclear NMR spectroscopy. The adduct **[2]$^-$** shows two signals in ^{11}B{^{1}H}-NMR spectrum (broad signal at 37.3 ppm for the sp^2 boron and a sharp peak at 4.8 ppm for the sp^3 boron), while the vinyl signals appeared in the ^{1}H-NMR spectrum at 6.12 and 5.14 ppm. The singlet at 5.30 ppm is attributed to ethene due to protic traces, in accordance with the literature.[2]

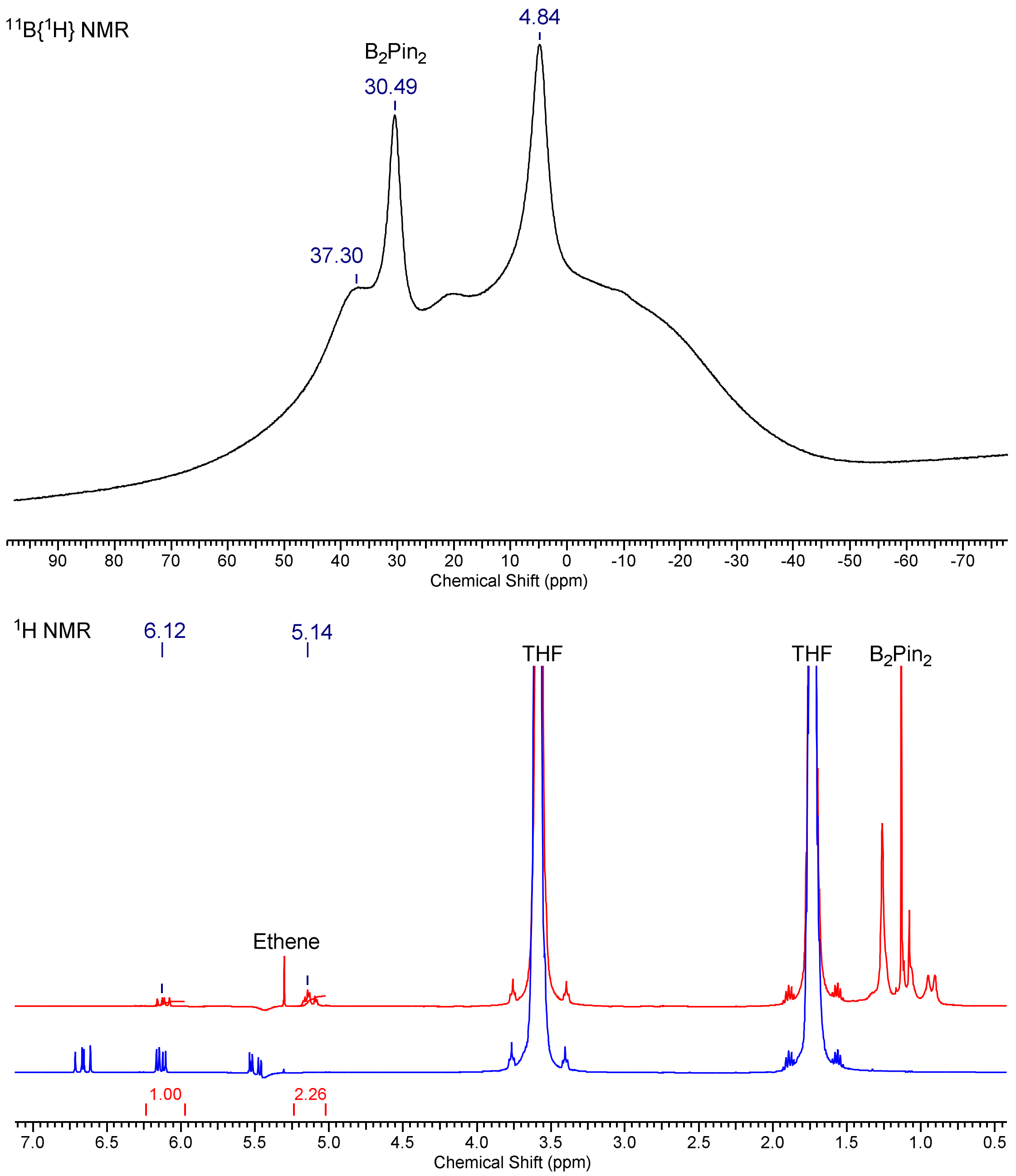

Figure S1. In-situ ^{1}H and $^{11}B\{^{1}H\}$-NMR spectra of an equimolar mixture of B_2Pin_2 and vinyl Grignard in dry THF. Blue (reference NMR for vinylMgBr), red (the equimolar mixture after 2 hours at -78°C and 15 min at RT).

3. Borane activation of [2]$^-$

3.1 Addition of $B(C_6F_5)_3$

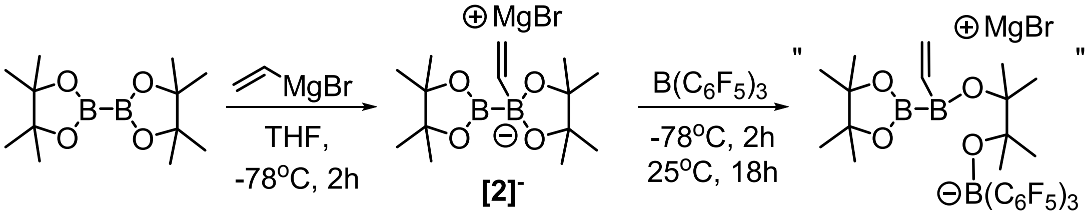

In a J. Young NMR tube, B_2Pin_2 (15 mg, 0.057 mmol, 1.0 eq.) was dissolved in dry THF (0.3 mL) and the solution was then cooled down to -78°C. After 5 min, a 1 M solution of vinyl magnesium bromide (57 µL, 0.057 mmol, 1.0 eq.) was added. The solution was kept at -78°C for 2 hours, with the J. Young NMR tube inverted each 30 min . While still at -78°C, a solution of $B(C_6F_5)_3$ (30 mg, 0.057 mmol, 1.0 eq.) in THF (0.2 mL) was added to the solution. The solution was kept at -78°C for 2 hours, with the J. Young NMR tube inverted each 30 min. The sample was left warming to room temperature and then was monitored by multi-nuclear NMR spectroscopy, revealing significant decrease in the resonances attributable to **[2]**$^-$ and the formation of an alkoxy-$B(C_6F_5)_3$ species, as confirmed by the resonance at -3.2 ppm in the $^{11}B\{^1H\}$-NMR spectrum ([alkyl-$B(C_6F_5)_3$] have a resonance around -15 ppm). The nature of the anionic species was further confirmed by the $\Delta\delta^{19}F$ < 4ppm between the *ortho*- and *para*-fluorine of the phenyl groups in the $^{19}F\{^1H\}$-NMR spectrum. In the $^{11}B\{^1H\}$-NMR spectrum a new resonances at 48.0 appeared as well, typically of borinic acid / $R_2B(OR)$ species. Moreover, the vinyl signals still persisted, although with a different chemical shift compared to those of **[2]**$^-$. The J. Young NMR tube was then left at room temperature for 18 hours and periodically inverted. After this time, the alkoxy-$B(C_6F_5)_3$ species was found as major product (by multinuclear NMR spectroscopy). An aliquot of the sample was analysed by ESI-MS (negative mode), observing the following alkoxy species as major products:

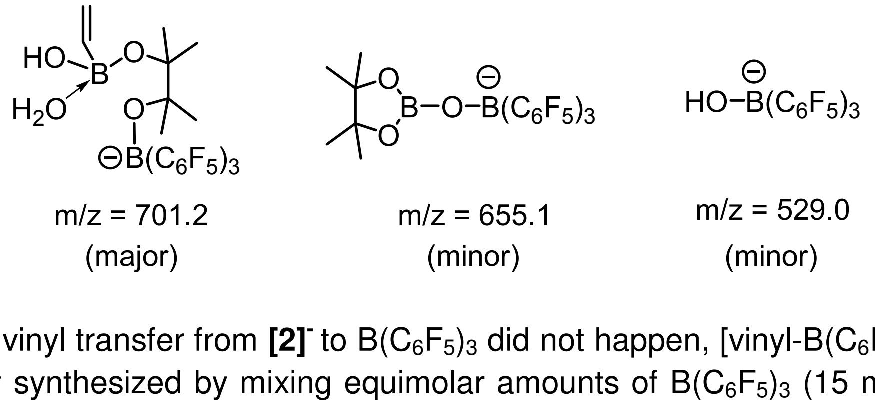

To prove that vinyl transfer from **[2]**$^-$ to $B(C_6F_5)_3$ did not happen, [vinyl-$B(C_6F_5)_3$]$^-$ was independently synthesized by mixing equimolar amounts of $B(C_6F_5)_3$ (15 mg, 0.028 mmol, 1.0 eq.) and vinyl magnesium bromide (28 µL, 0.028 mmol, 1.0 eq.) in dry THF (observed $^{11}B\{^1H\}$-NMR = -14.60 ppm).

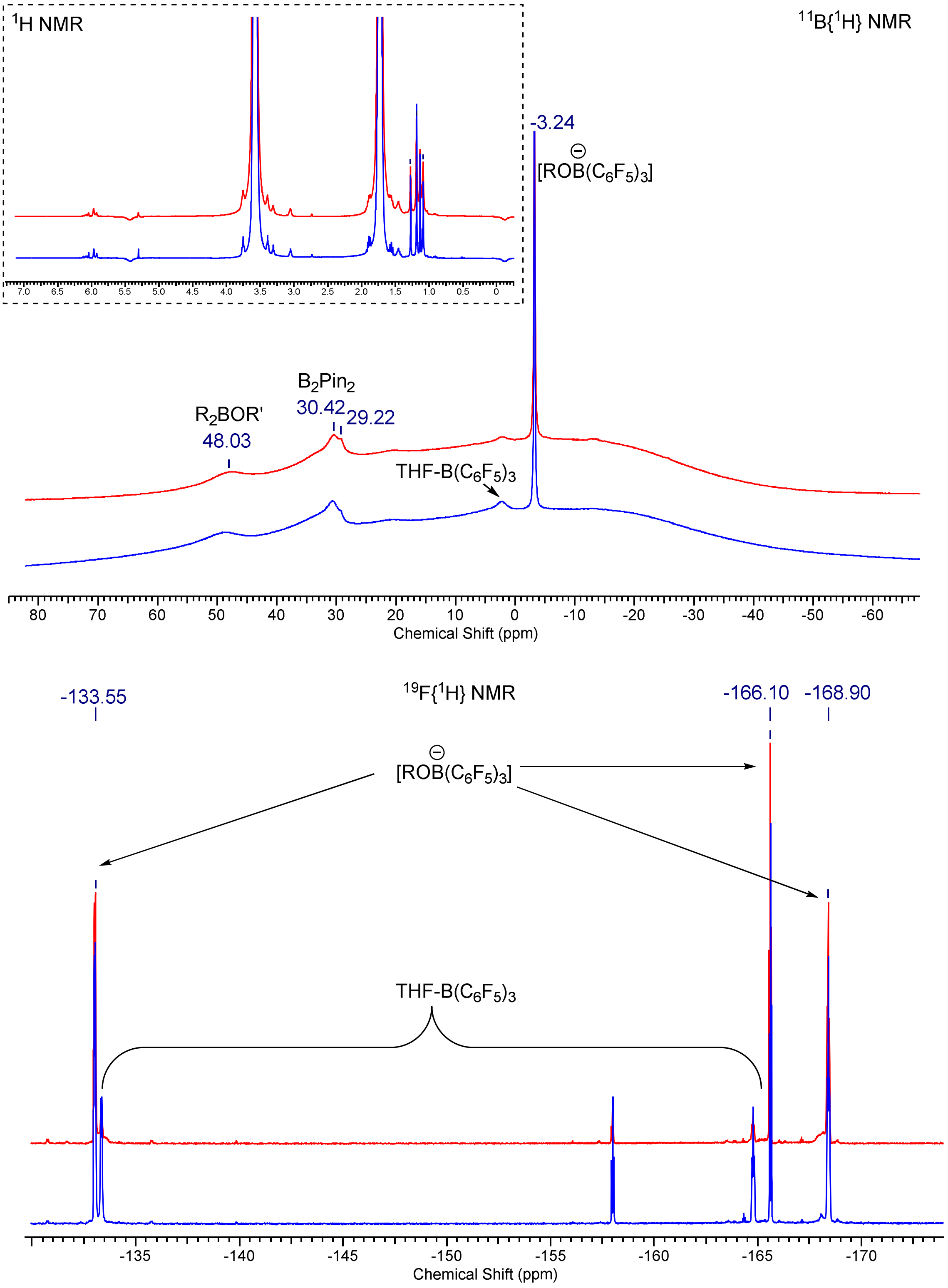

Figure S2. In-situ ^{1}H, $^{11}B\{^{1}H\}$, and $^{19}F\{^{1}H\}$-NMR spectra of an equimolar mixture of B_2Pin_2, vinylMgBr and $B(C_6F_5)_3$ in dry THF. Blue (after 2 hours at -78°C), red (after 18 hours at RT).

3.2 Addition of BPh_3

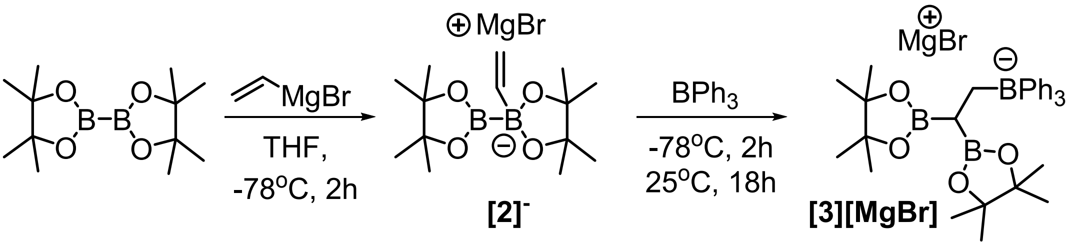

In a J. Young NMR tube, B_2Pin_2 (15 mg, 0.057 mmol, 1.0 eq.) was dissolved in dry THF (0.3 mL) and the solution was then cooled down to -78°C. After 5 min, a 1 M solution of vinyl magnesium bromide (57 µL, 0.057 mmol, 1.0 eq.) was added. The solution was kept at -78°C for 2 hours, with the J. Young NMR tube inverted each 30 min. While still at -78°C, a 0.25 M THF solution of BPh_3 (227 µL, 0.057 mmol, 1.0 eq.) was added to the solution. The solution was kept at -78°C for 2 hours, with the J. Young NMR tube inverted each 30 min. The sample was left warming to room temperature and then was monitored by multi-nuclear NMR spectroscopy, revealing the formation of the desired product **[3]⁻** ($^{11}B\{^1H\}$-NMR signals at 34.7 ppm [-BPin moieties] and -9.5 ppm [$RBPh_3^-$]). Moreover, inequivalent signals for the methyl groups and a broad signal at 0.55 ppm for the aliphatic C-H were observed. The J. Young NMR tube was then left at room temperature for 18 hours and periodically inverted. After this time, **[2]⁻** had completely reacted giving significant amount of **[3]⁻**. Mesitylene addition (10 µL, 0.070 mmol, 1.24 eq.) allowed the determination of the in-situ yield by the relative integration of the aromatic signal of mesitylene and the aromatic resonances of **[3]⁻** (NMR yield = 71%). The reaction was then repeated on a bigger scale in a Schlenk Flask with stirring (B_2Pin_2 = 100 mg, THF = 5 mL, vinyl-MgBr = 0.38 mL, BPh_3 = 1.51 mL) and the product was isolated by removing THF (under N_2) and washing with dry Et_2O (2 x 5 mL), obtaining **[3][MgBr(THF)$_2$]** as a white powder (209 mg, 70% yield).

^1H-NMR (400 MHz, d_8-THF) = 7.30 ppm (d, J = 7.28 Hz, 6H, -Ph), 6.80 ppm (t, J = 7.28 Hz, 6H-Ph), 6.64 ppm (t, J = 7.28 Hz, 3H-Ph), 1.29 ppm (brs, 2H, $-CH_2-$), 1.04 ppm (s, 12H, 4 $-CH_3$), 1.03 ppm (s, 12H, 4 $-CH_3$), 0.59 ppm (brs, 1H, -CH-). *THF coordinated to the magnesium appears at 3.62 ppm (m, 8H) and 1.77 ppm (m, 8H).* $^{11}B\{^1H\}$-NMR (128 MHz, d_8-THF) = 35.7 ppm (brs, 2B, RBPin), -9.4 ppm (s, 1B, $RBPh_3$). $^{13}C\{^1H\}$-NMR (100 MHz, d_8-THF) = 167.78 (3C, *ipso*-Ph), 136.39 ppm (6C, *o*-Ph), 125.68 ppm (6C, *m*-Ph), 121.33 ppm (3C, *p*-Ph), 81.78 ppm (4C, C-O), 25.50 ppm (4 CH_3), 20.84 ppm (1C, CH_2), 8.57 ppm (1C, CH) : the carbons attached to the boron atoms (not directly observed) were assigned by HMBC/HSQC analysis, while the signal of the other 4 CH_3 of pinacol is presumably hidden by the solvent peak. *THF coordinated to magnesium appears at 68.31 ppm and 26.43 ppm.* Accurate mass for the anion **[3]⁻**($[C_{32}H_{42}B_3O_4]^-$): 523.3364 (expected: 523.3368).

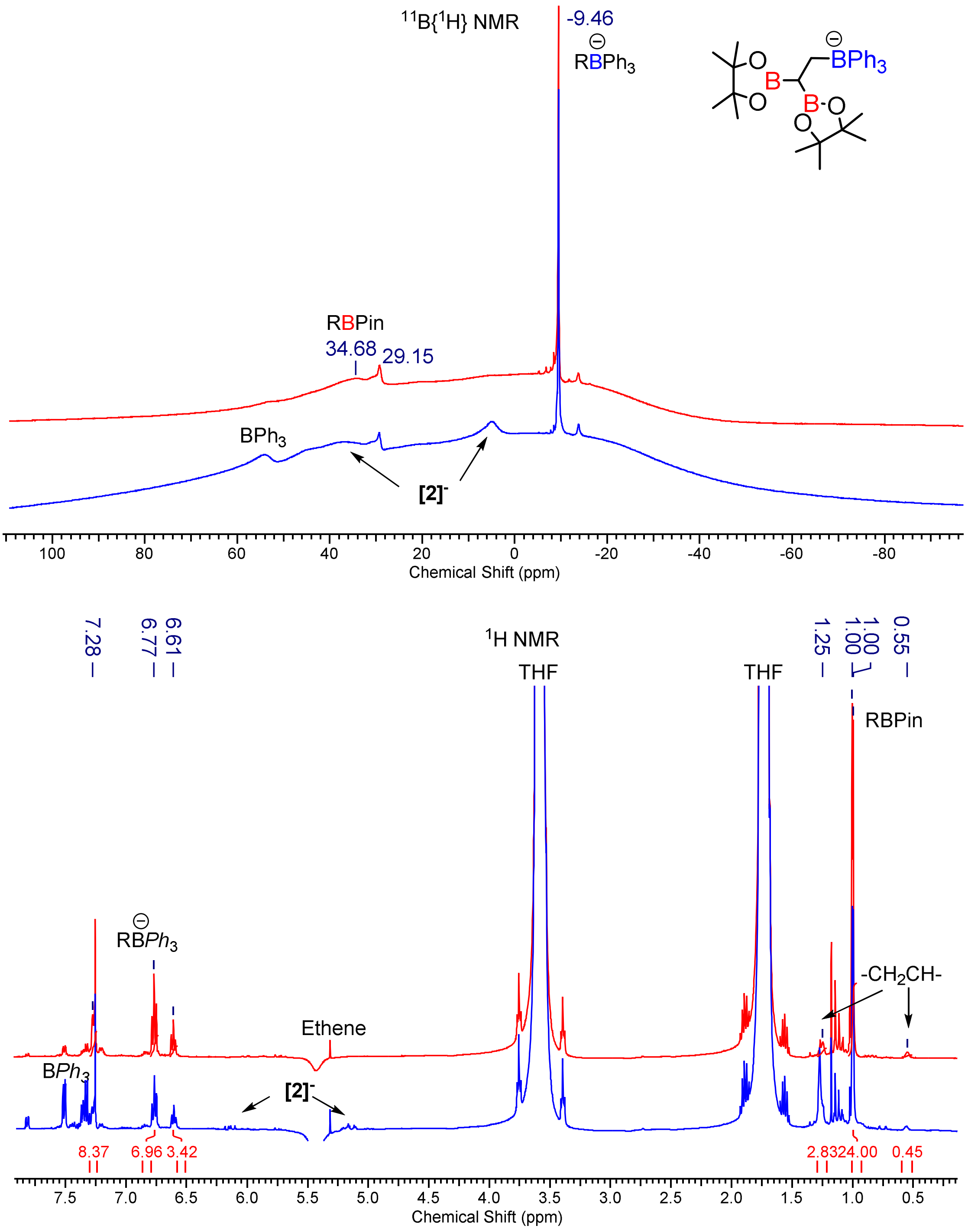

Figure S3. In-situ ^1H and $^{11}B\{^1H\}$-NMR spectra of an equimolar mixture of B_2Pin_2, vinylMgBr and BPh_3 in dry THF. Blue (after 2 hours at -78°C and 10 minutes at RT), red (after a further 18 hours at RT).

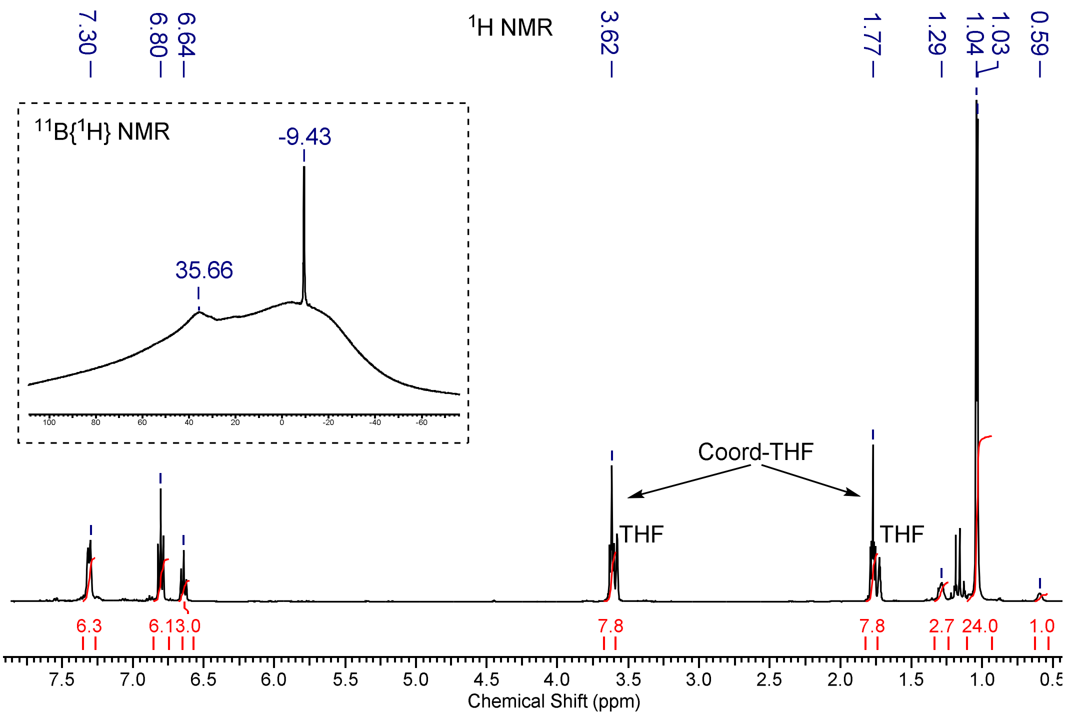

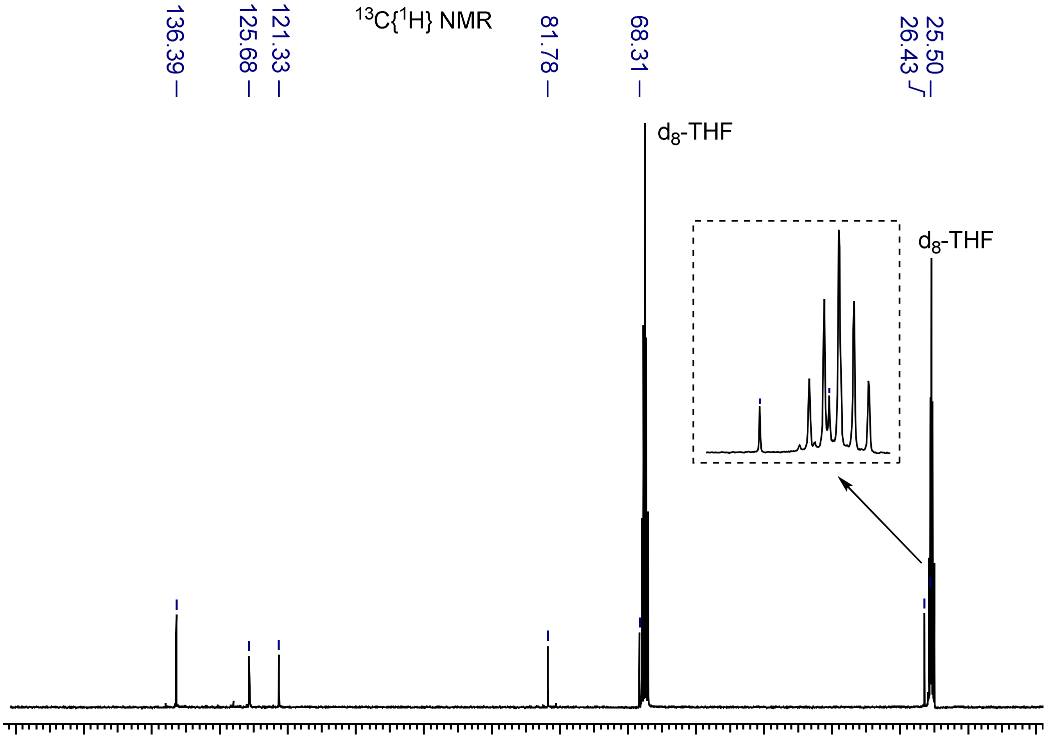

Figure S4. ^{1}H, $^{11}B\{^{1}H\}$, $^{13}C\{^{1}H\}$-NMR spectra of the isolated **[3][MgBr(THF)$_2$]** in d_8-THF.

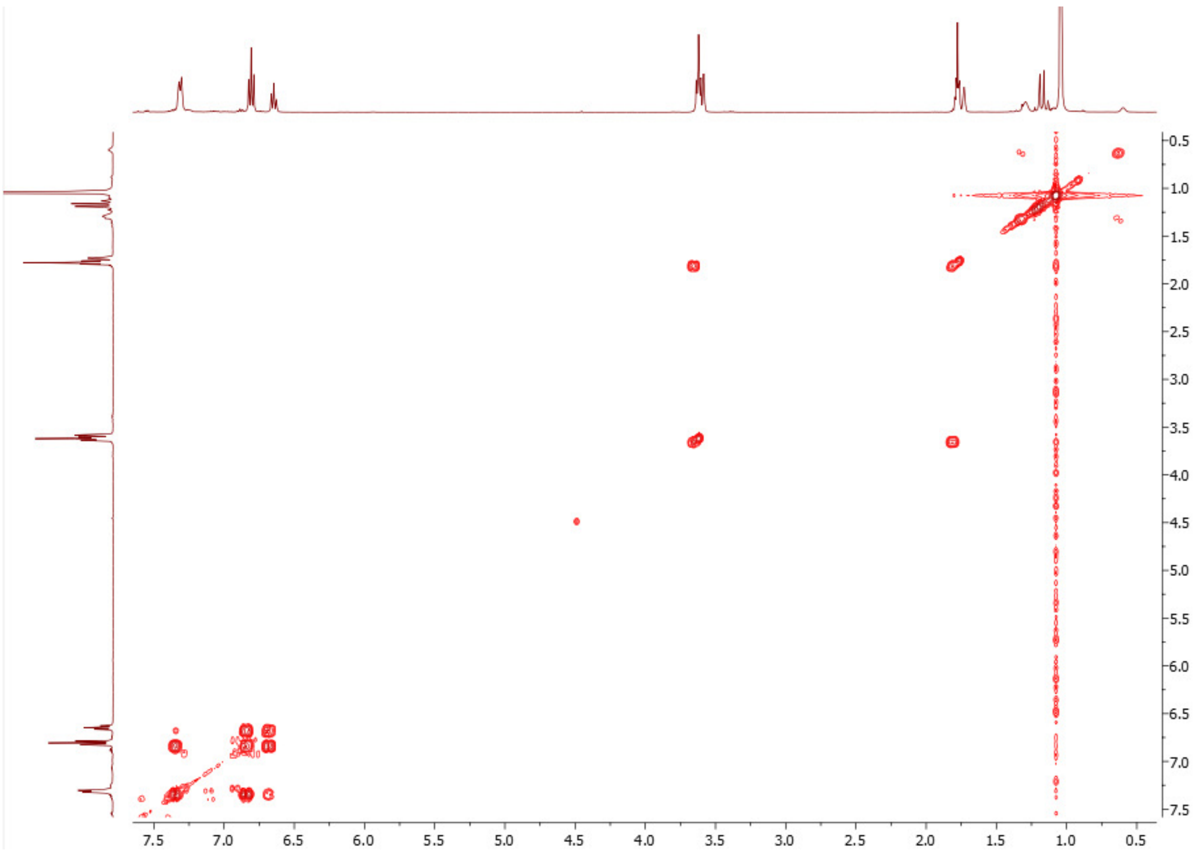

Figure S5. ^{1}H-^{1}H COSY-NMR spectrum of the isolated **[3][MgBr(THF)$_2$]** in d_8-THF.

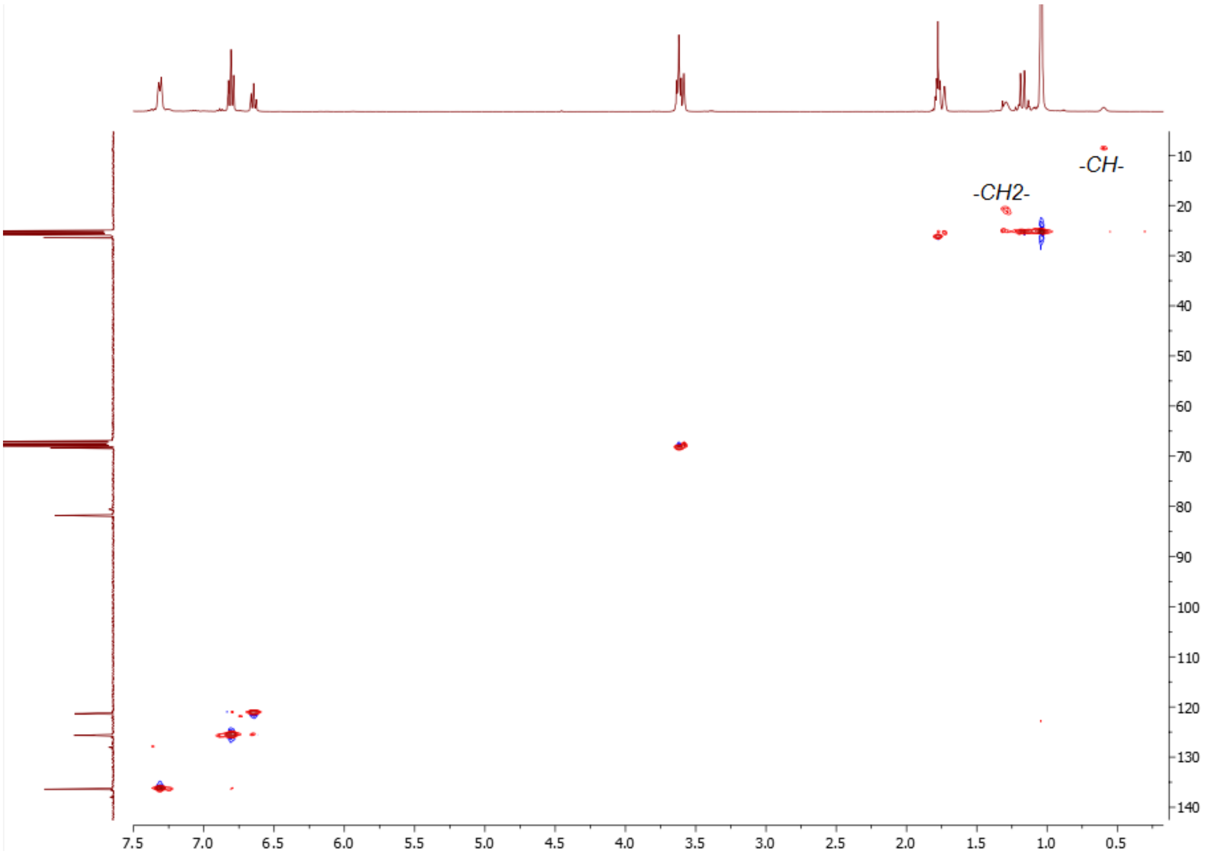

Figure S6. ^{1}H-^{13}C HSQC-NMR spectrum of the isolated **[3][MgBr(THF)$_2$]** in d_8-THF.

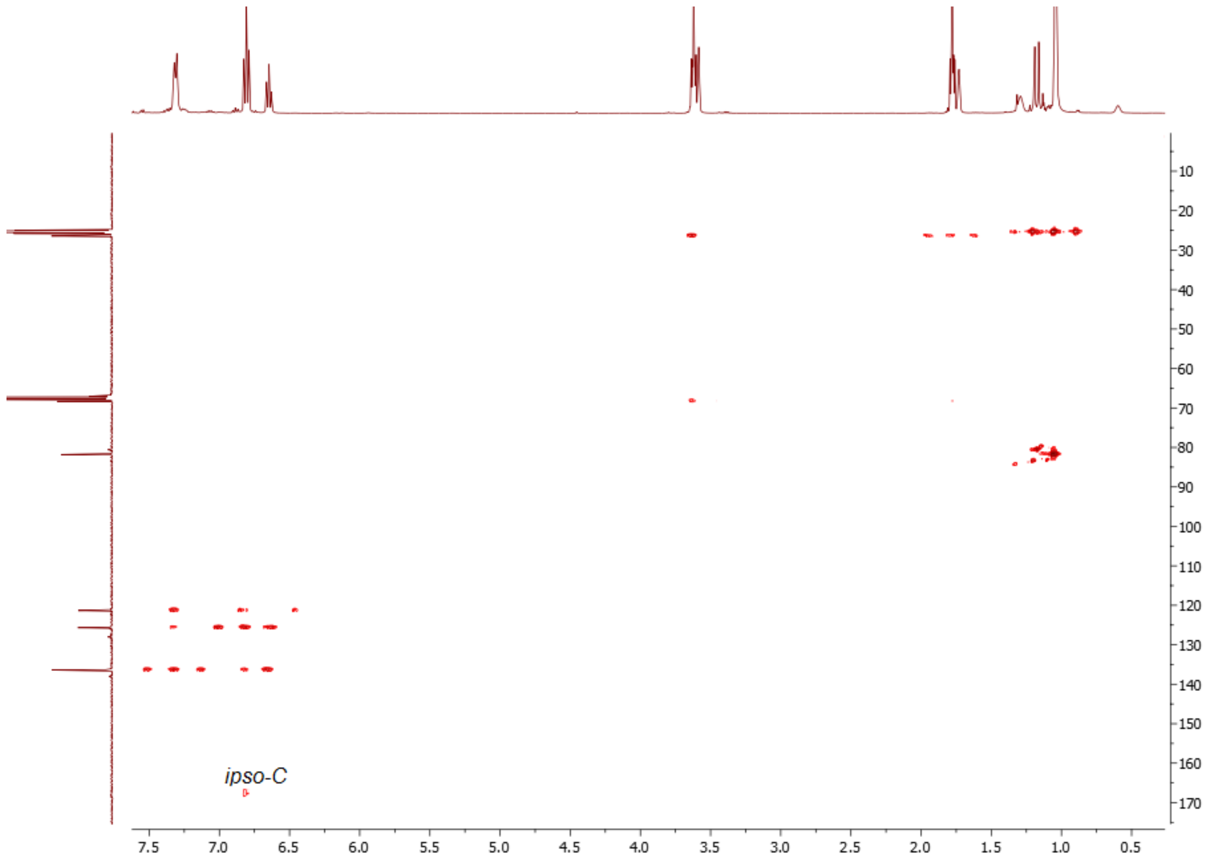

Figure S7. ^{1}H-^{13}C HMBC-NMR spectrum of the isolated **[3][MgBr(THF)$_2$]** in d8-THF.

Suitable crystals for XRD analysis were obtained layering dry pentane onto the THF solution and leaving undisturbed for 3 days. See section 9 for full crystallographic details.

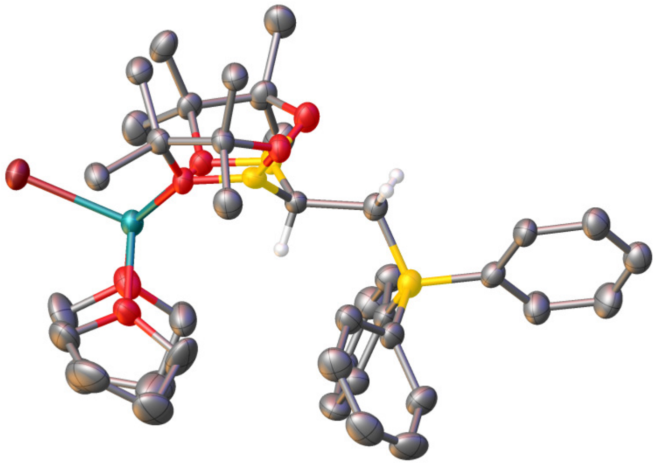

Figure S1. Crystal structure of **[3][MgBr(THF)$_2$]** with ellipsoids depicted at 50% probability (some hydrogen atoms are omitted for clarity). Grey=carbon, white=hydrogen, red=oxygen, yellow=boron, blue=magnesium, dark red=bromo.

The cation could be replaced with $[Me_4N]^+$ via salt metathesis in dry CH_2Cl_2 using 1.5 equivalent of $[Me_4N][Cl]$ (relative to **[3][MgBr(THF)$_2$]**), followed by filtration of $MgBrCl(solv)_x$ and subsequent washing with pentane, obtaining **[3][Me$_4$N]** as a white solid.

[3][Me$_4$N] ^{1}H-NMR (400 MHz, CH_2Cl_2) = 7.31 ppm (m, 6H, -Ph), 6.99 ppm (t, J = 7.28 Hz, 6H-Ph), 6.84 ppm (t, J = 7.28 Hz, 3H-Ph), 2.33 ppm (s, 12H, NMe_4), 1.20 ppm (brs, 2H, $-CH_2-$), 1.09 ppm (s, 24H, 8 $-CH_3$), 0.38 ppm (brs, 1H, -CH-). ^{11}B{^{1}H}-NMR (128 MHz, CH_2Cl_2) = 35.35 ppm (brs, 2B, RBPin), -9.23 ppm (s, 1B, $RBPh_3$). ^{13}C{^{1}H}-NMR (100 MHz, CH_2Cl_2) = 135.67 ppm (6C, *o*-Ph), 126.33 ppm (6C, *m*-Ph), 122.22 ppm (3C, *p*-Ph), 82.40 ppm (4C, C-O), 56.13 ppm (4C, Me_4N), 25.27 ppm (8 CH_3): the carbons attached to the boron atoms were not observed.

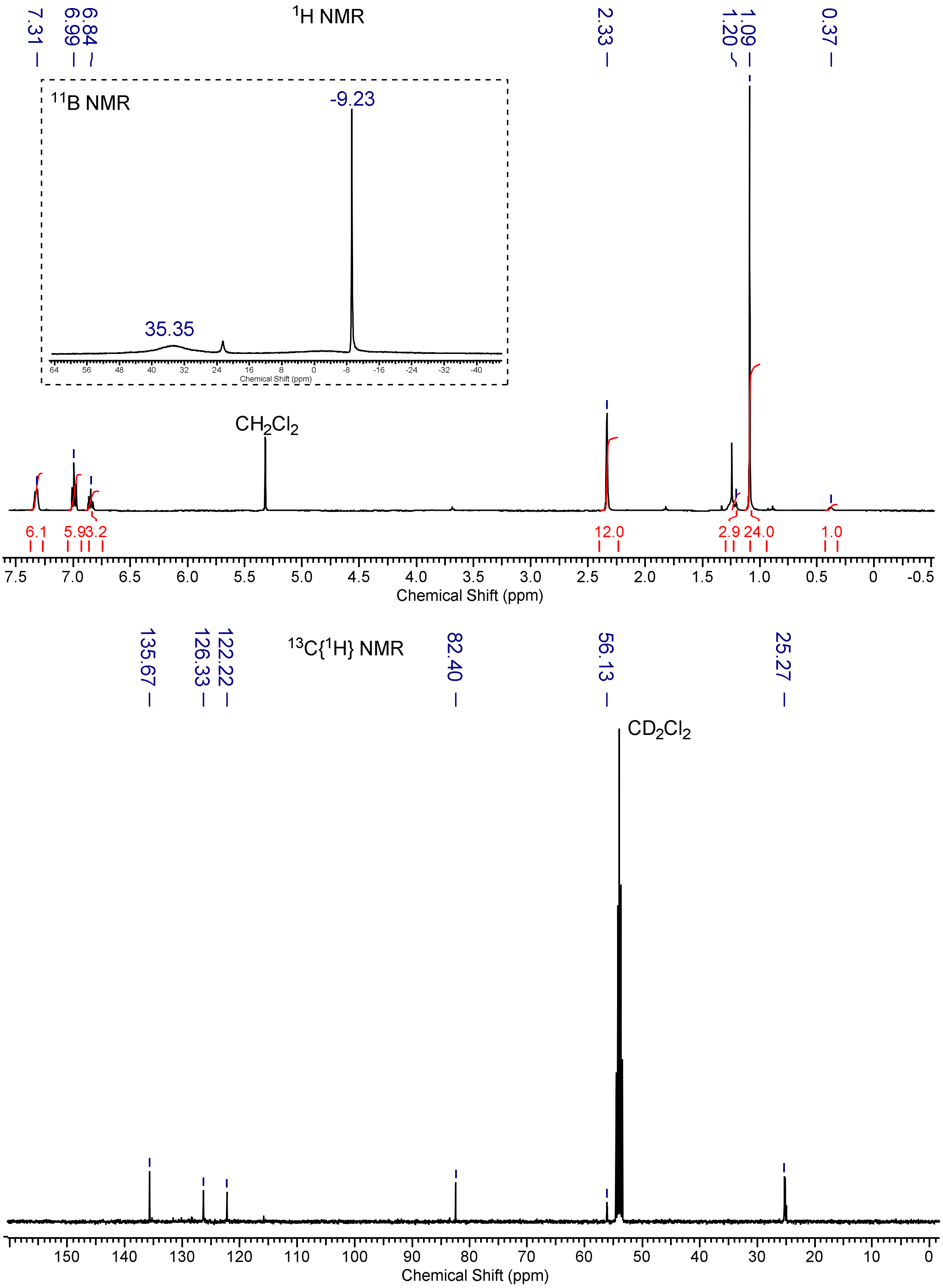

Figure S8. ^{1}H, ^{11}B, $^{13}C\{^{1}H\}$-NMR spectra of the isolated **[3][Me$_4$N]** in CD_2Cl_2.

3.3 Addition of 9-Ph-BBN

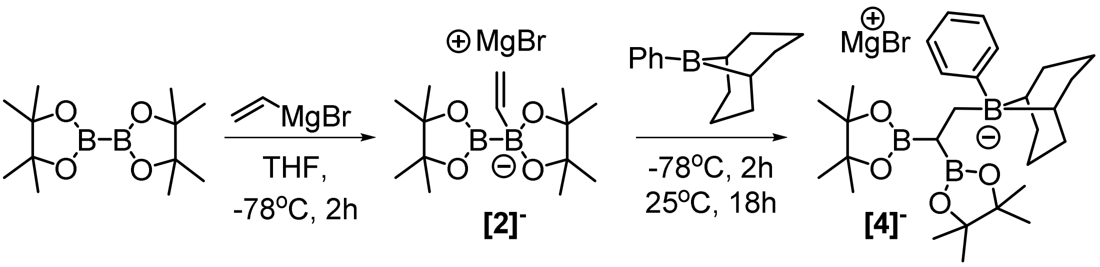

In a J. Young NMR tube, B_2Pin_2 (30 mg, 0.113 mmol, 1.0 eq.) was dissolved in dry THF (0.5 mL) and the solution was then cooled down to -78°C. After 5 min, a 1M solution of vinyl magnesium bromide (114 µL, 0.113 mmol, 1.0 eq.) was added. The solution was kept at -78°C for 2 hours, with the J. Young NMR tube inverted each 30 min. While still at -78°C, 9-Ph-BBN (24 µL, 0.113 mmol, 1.0 eq.) was added to the solution. The solution was kept at -78°C for 2 hours, with the J. Young NMR tube inverted each 30 min. The sample was left warming to room temperature and then was monitored by multi-nuclear NMR spectroscopy, revealing the formation of the desired product **[4]$^-$** ($^{11}B\{^1H\}$-NMR signals at 34.0 ppm [-BPin moieties] and -15.3 ppm [R(Ph)BBN$^-$]). Moreover, inequivalent signals for the methyl groups and a broad signal at 0.24 ppm for the aliphatic C-H were observed. The J. Young NMR tube was then left at room temperature for 18 hours and periodically inverted. After this time, a significant amount of **[4]$^-$** was observed, along with minor amounts of unreacted **[2]$^-$** and 9-Ph-BBN. The reaction was then repeated on a bigger scale (B_2Pin_2 = 300 mg, vinyl-MgBr = 1.14 mL, 9-Ph-BBN = 0.24 mL) and the product was isolated removing THF (under N_2) and washing with dry Et_2O (2 x 5 mL), obtaining the **[4][MgBr(THF)$_2$]** as a white powder (430 mg, 52% yield including two molecules of THF based on ^1H NMR spectroscopy and by analogy to **[3][MgBr(THF)$_2$]**). *Due to restricted rotation of the tetra-coordinated boron centre, desymmetrization of the bicyclo moiety is observed (diastereotopic signals).*

^1H-NMR (400 MHz, d_8-THF) = 7.27 ppm (m, 2H, -Ph), 6.80 ppm (t, J = 7.28 Hz, 2H-Ph), 6.56 ppm (t, J = 7.28 Hz, 1H-Ph), 2.24 ppm (m, 2H, -BBN), 1.85 ppm (m, 3H, -BBN), 1.78 ppm (m, 1H, BBN), 1.47 ppm (m, 3H, -BBN), 1.31 ppm (m, 2H, -BBN), 1.21 ppm (m, 1H, -BBN), 1.01 ppm (s, 12H, 4 -CH_3), 0.99 ppm (s, 12H, 4 -CH_3), 0.79 ppm (m, 2H, -BBN), 0.48 ppm (brs, 2H, -CH_2-), 0.30 ppm (brs, 1H, -CH-). *THF coordinated to the magnesium appears at 3.62 ppm (m, 8H) and 1.78 ppm (m, 8H).* $^{11}B\{^1H\}$-NMR (128 MHz, d_8-THF) = 35.7 ppm (brs, 2B, RBPin), -9.4 ppm (s, 1B, $RBPh_3$). $^{13}C\{^1H\}$-NMR (100 MHz, d_8-THF) = 169.80 ppm (1C, *ipso*-Ph), 134.77 ppm (2C, *o*-Ph), 125.58 ppm (2C, *m*-Ph), 120.25 ppm (1C, *p*-Ph), 81.43 ppm (4C, C-O), 34.86 (2C, -CH_2- BBN), 34.29 (2C, -CH_2- BBN), 28.81 (1C, -CH_2- BBN), 27.99 (1C, -CH_2- BBN), 26.88 ppm (2C, -C(B)H- BBN), 25.43 ppm (4 CH_3), 25.25 (4 CH_3), 22.38 ppm (-CH_2-), 5.39 ppm (-CH-): the carbons attached to the boron atoms were assigned by HMBC/HSQC analysis. *THF coordinated to magnesium appears at 68.33 ppm and 26.43 ppm.* Accurate mass for the anion **[4]$^-$** ($[C_{28}H_{46}B_3O_4]^-$): 479.3673 (expected: 479.3681).

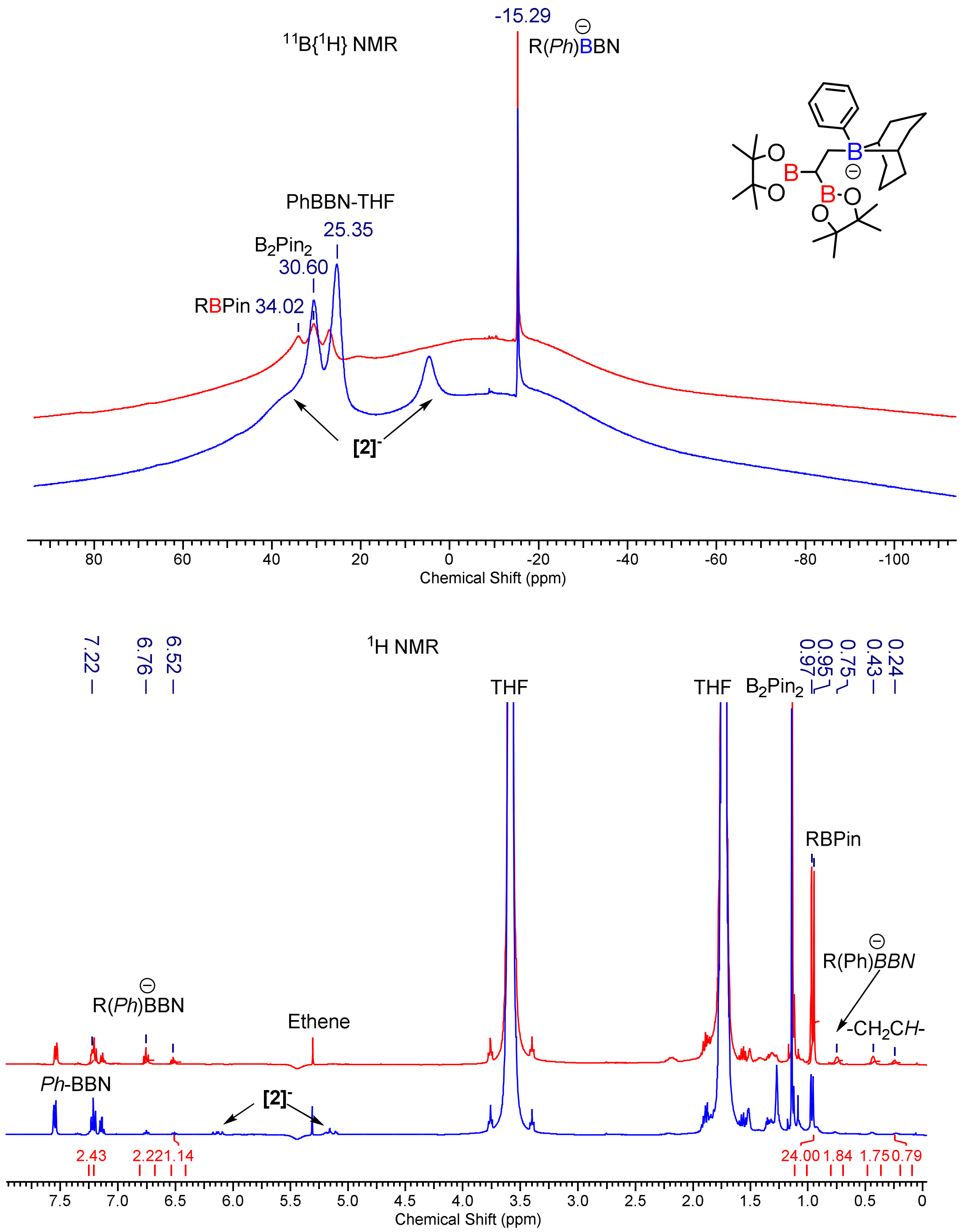

Figure S9. In-situ ^{1}H and $^{11}B\{^{1}H\}$-NMR spectra of an equimolar mixture of B_2Pin_2, vinylMgBr and 9-Ph-BBN in dry THF. Blue (after 2 hours at -78°C and 10 mins at RT), red (after 18 hours at RT).

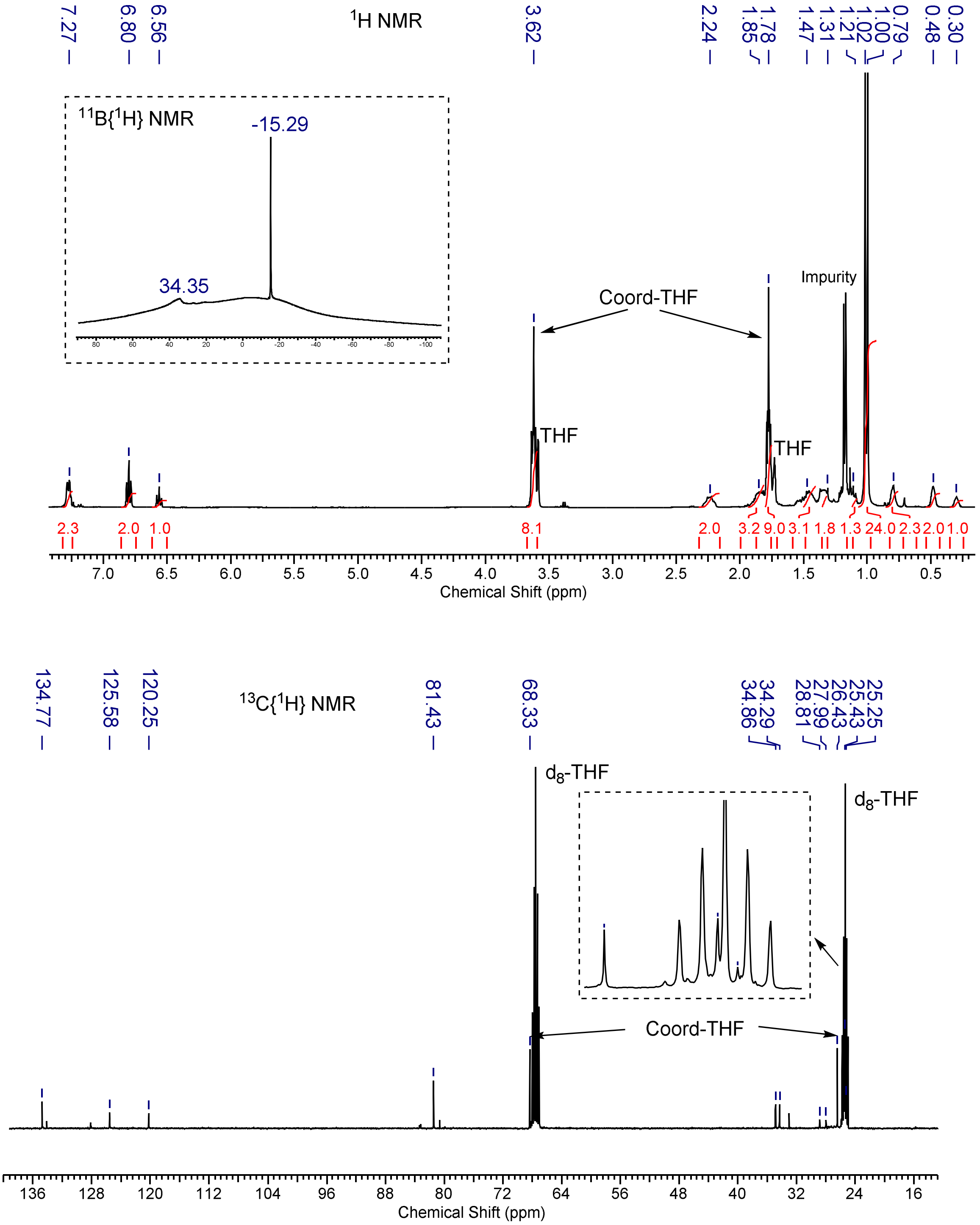

Figure S10. ^{1}H, $^{11}B\{^{1}H\}$, $^{13}C\{^{1}H\}$-NMR spectra of the isolated **[4][MgBr(THF)$_2$]** in d_8-THF.

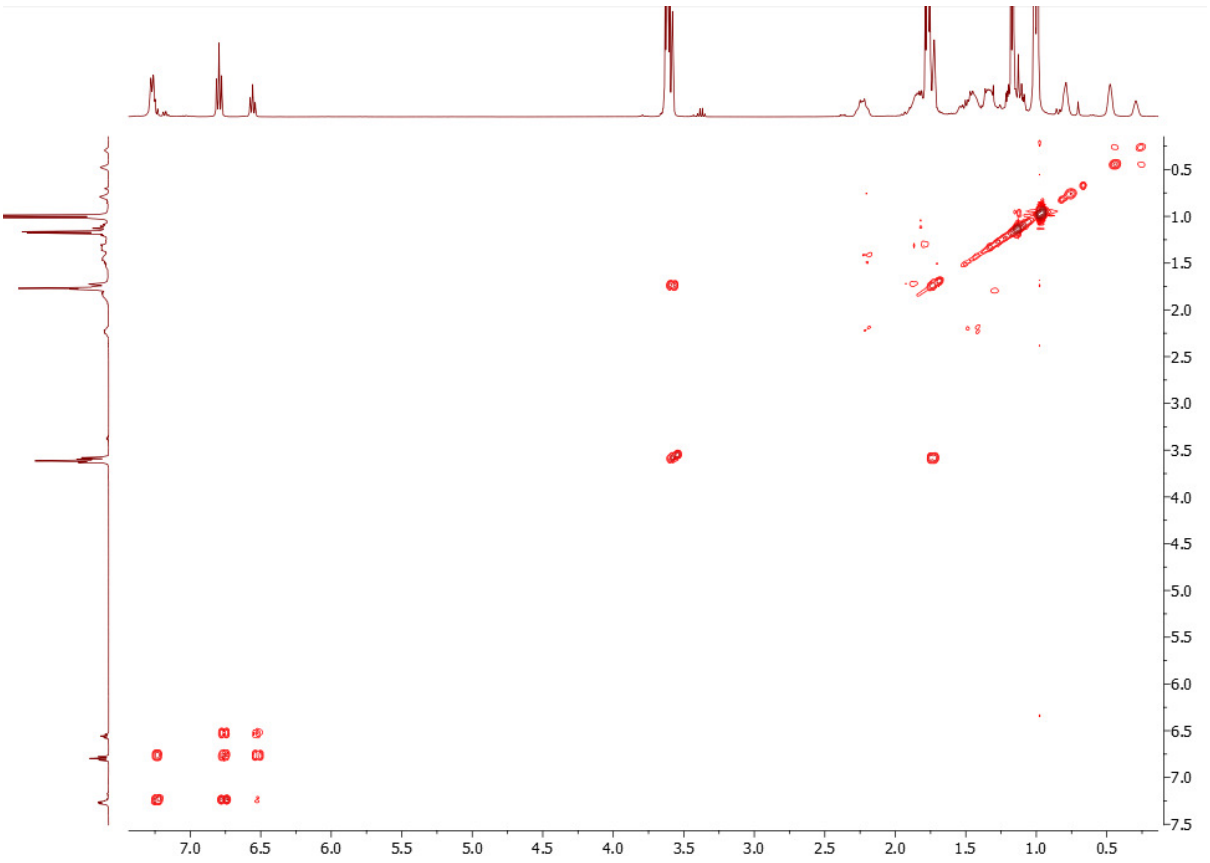

Figure S11. ^{1}H-^{1}H COSY-NMR spectrum of isolated **[4][MgBr(THF)$_2$]** in THF.

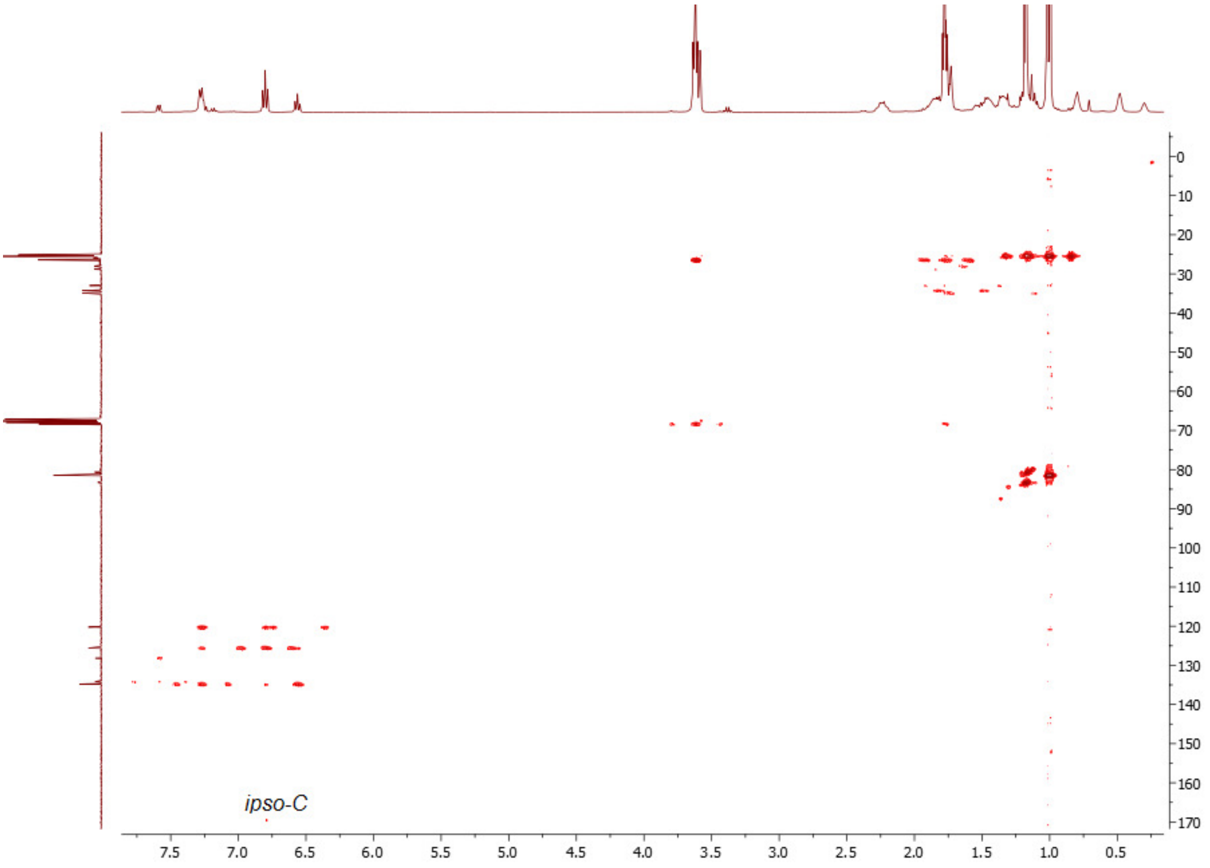

Figure S12. ^{1}H-^{13}C HSQC-NMR spectrum of isolated **[4][MgBr(THF)$_2$]** in d_8-THF.

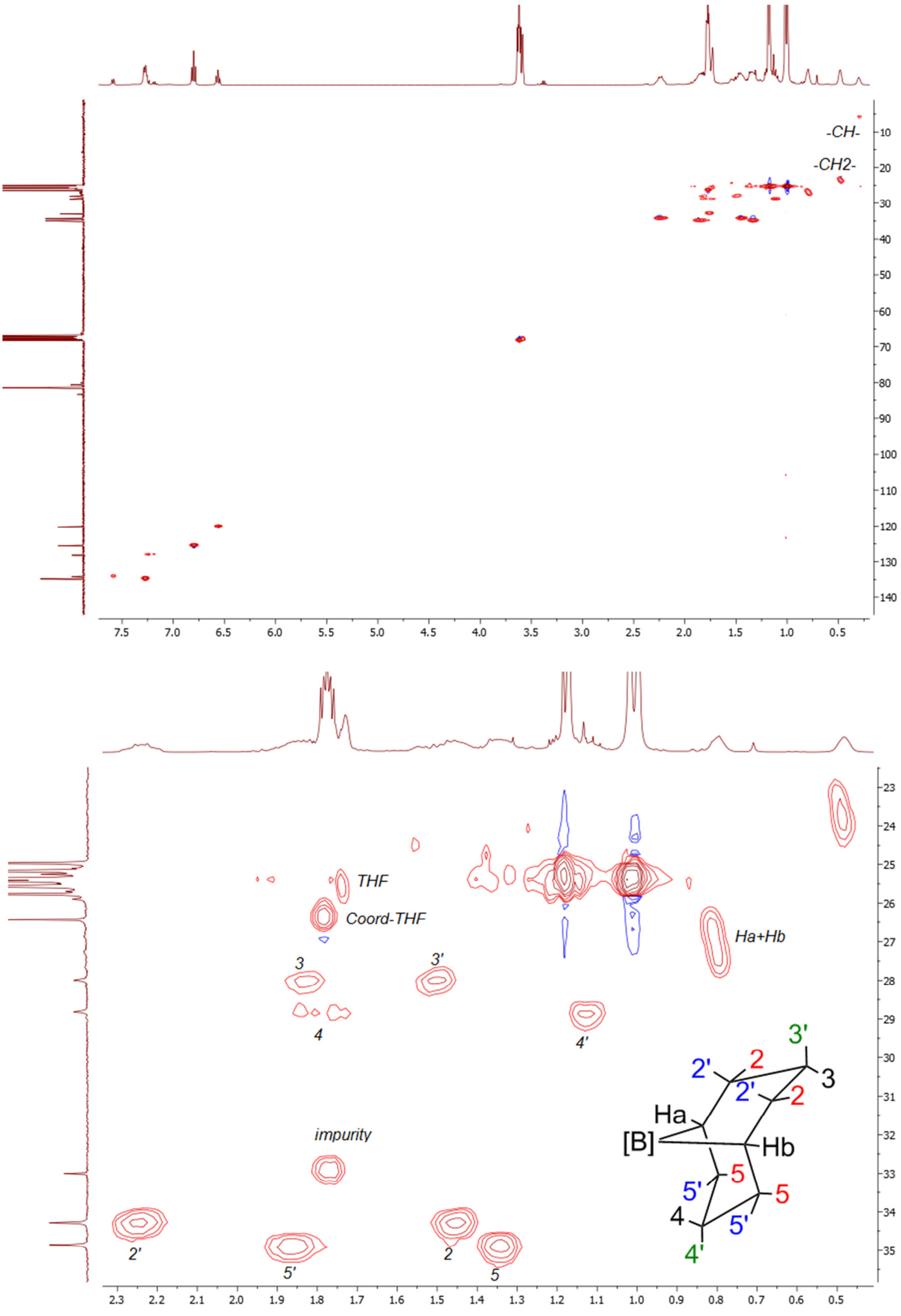

Figure S13. ^{1}H-^{13}C HMBC-NMR spectrum of the isolated **[4][MgBr]** in d8-THF.

3.4 Addition of 9-Mesityl-BBN to $[2]^-$

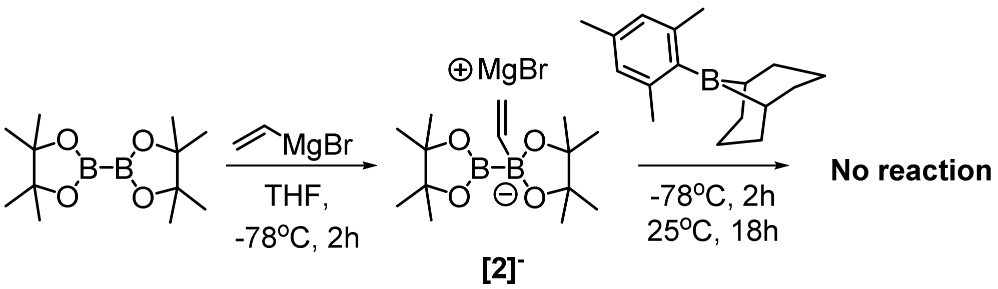

In a J. Young NMR tube, B_2Pin_2 (30 mg, 0.113 mmol, 1.0 eq.) was dissolved in dry THF (0.5 mL) and the solution was then cooled down to -78°C. After 5 min, a 1M solution of vinyl magnesium bromide (114 µL, 0.113 mmol, 1.0 eq.) was added. The solution was kept at -78°C for 2 hours, with the J. Young NMR tube inverted each 30 min. While still at -78°C, 9-Mesityl-BBN (33 µL, 0.113 mmol, 1.0 eq.) was added to the solution. The solution was kept at -78°C for 2 hours, with the J. Young NMR tube inverted each 30 min. The sample was left warming to room temperature and then was monitored by multi-nuclear NMR spectroscopy, revealing no reaction. The steric hindrance around the boron centre prevents THF binding as well (in contrast to PhBBN).

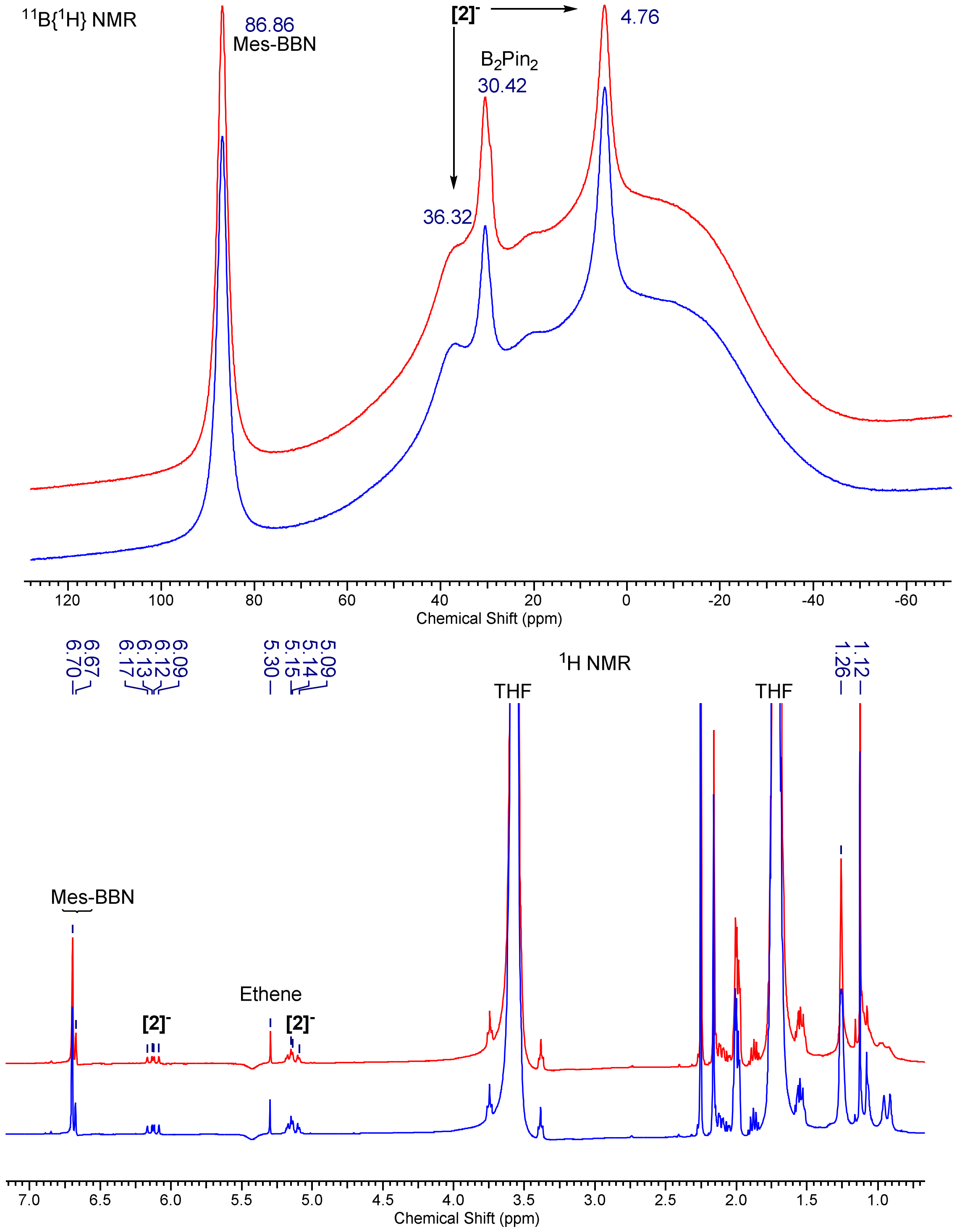

Figure S14. In-situ ^1H and $^{11}B\{^1H\}$-NMR spectra of an equimolar mixture of B_2Pin_2, vinylMgBr and 9-Mesityl-BBN in dry THF. Blue (after 2 hours at -78°C and 10 mins at RT), red (after 18 hours at RT).

3.5 Addition of 9-*o*-tolyl-BBN

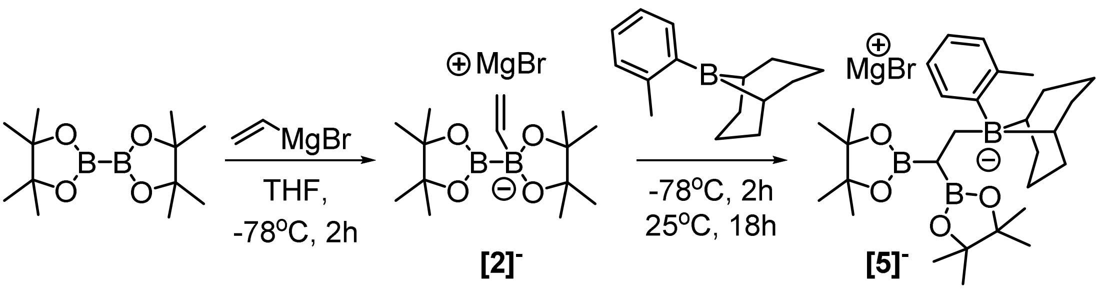

In a J. Young NMR tube, B_2Pin_2 (30 mg, 0.113 mmol, 1.0 eq.) was dissolved in dry THF (0.5 mL) and the solution was then cooled down to -78°C. After 5 min, a 1M solution of vinyl magnesium bromide (114 µL, 0.113 mmol, 1.0 eq.) was added. The solution was kept at -78°C for 2 hours, with the J. Young NMR tube was inverted each 30 min. While still at -78°C, 9-*o*-tolyl-BBN (25 µL, 0.113 mmol, 1.0 eq.) was added to the solution. The solution was kept at -78°C for 2 hours, with the J. Young NMR tube inverted each 30 min. The sample was left warming to room temperature and then was monitored by multi-nuclear NMR spectroscopy, revealing the partial formation of the desired product **[5]⁻** ($^{11}B\{^1H\}$-NMR signals at 34.2 ppm [-BPin moieties] and -13.9 ppm [R(Ph)BBN⁻], while the $-CH_2CH-$ appeared as broad signals at 0.55 ppm and 0.21 ppm). The J. Young NMR tube was then left at room temperature for 18 hours and periodically inverted. After this time, significant amount of product was observed, along with the unreacted **[2]⁻** and 9- *o*-tolyl-BBN. Mesitylene addition (10 µL, 0.070 mmol, 0.62 eq.) allowed the determination of the in-situ yield by the relative integration of the aromatic signal of mesitylene and the aromatic ones of **[5]⁻** (NMR yield = 31%). Due to the overlap of the BBN resonances for starting material and products, only diagnostic peaks are reported as attempts to isolate this product were unsuccessful in our hands (over a range of scales).
Diagnostic peaks: ^1H-NMR (400 MHz, THF) = 7.25 ppm (d, 1H, -Tolyl), 6.58 ppm (t, 1H, -Tolyl), 6.51 ppm (d, 1H, -Tolyl), 6.44 ppm (t, 1H, -Tolyl), 2.31 ppm (s, 3H, -Me), 1.05 ppm (s, 12H, 4 $-CH_3$), 0.85 ppm (s, 6H, 2 $-CH_3$), 0.83 ppm (s, 6H, 2 $-CH_3$), 0.55 ppm (brs, 2H, $-CH_2-$), 0.21 ppm (brs, 1H, -CH-). $^{11}B\{^1H\}$-NMR (128 MHz, THF) = 34.24 ppm (brs, 2B, RBPin), -13.94 ppm (s, 1B, R(Tol)BBN). Accurate mass for the anion **[5]⁻** ($[C_{29}H_{48}B_3O_4]^-$): 493.3831 (expected: 493.3837).

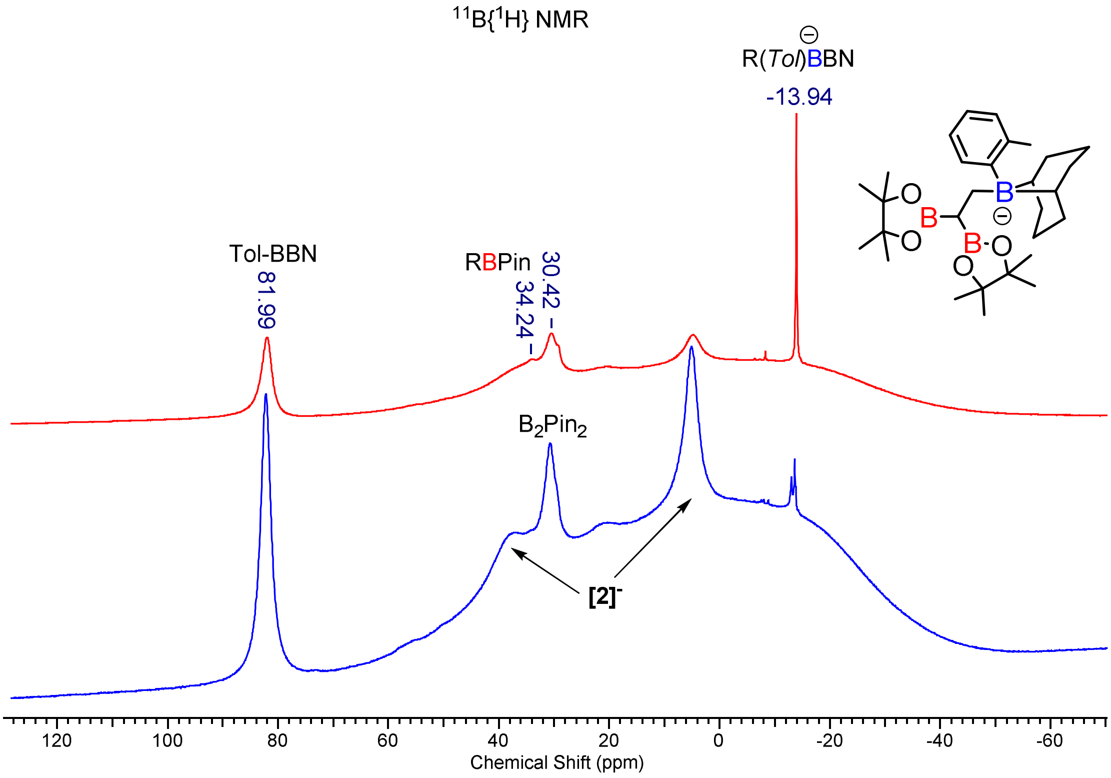

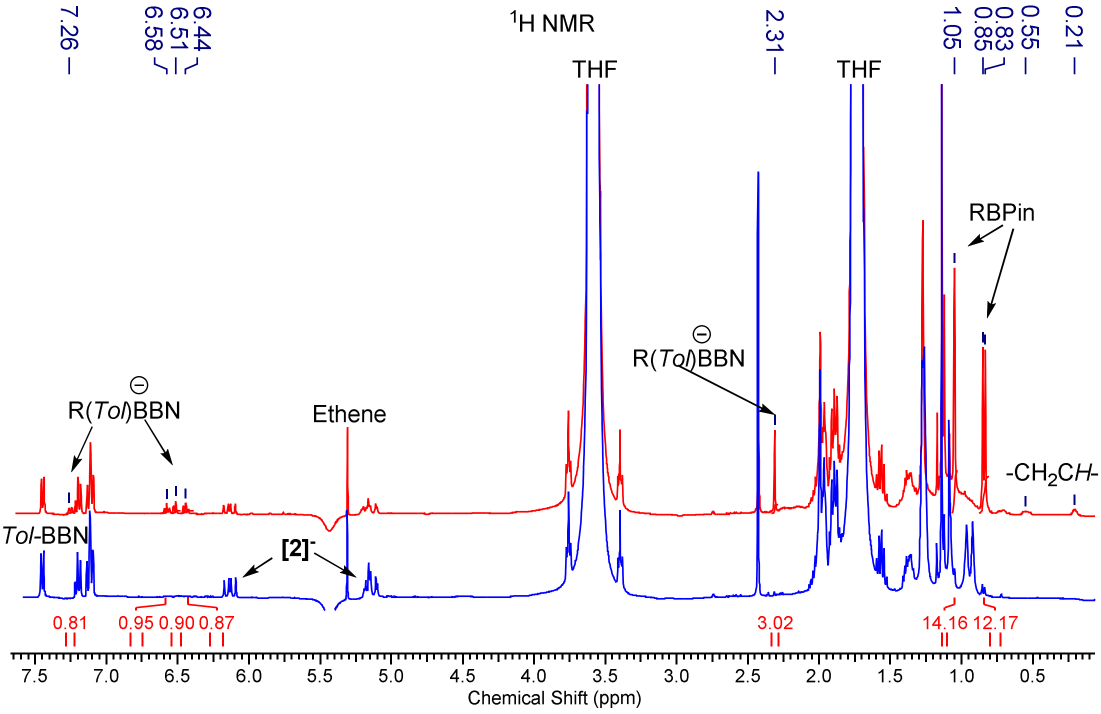

Figure S15. In-situ ^{1}H and ^{11}B{^{1}H}-NMR spectra of an equimolar mixture of B_2Pin_2, vinylMgBr and 9-*o*-tolyl-BBN in dry THF. Blue (after 2 hours at -78°C and 10 mins at RT), red (after 18 hours at RT).

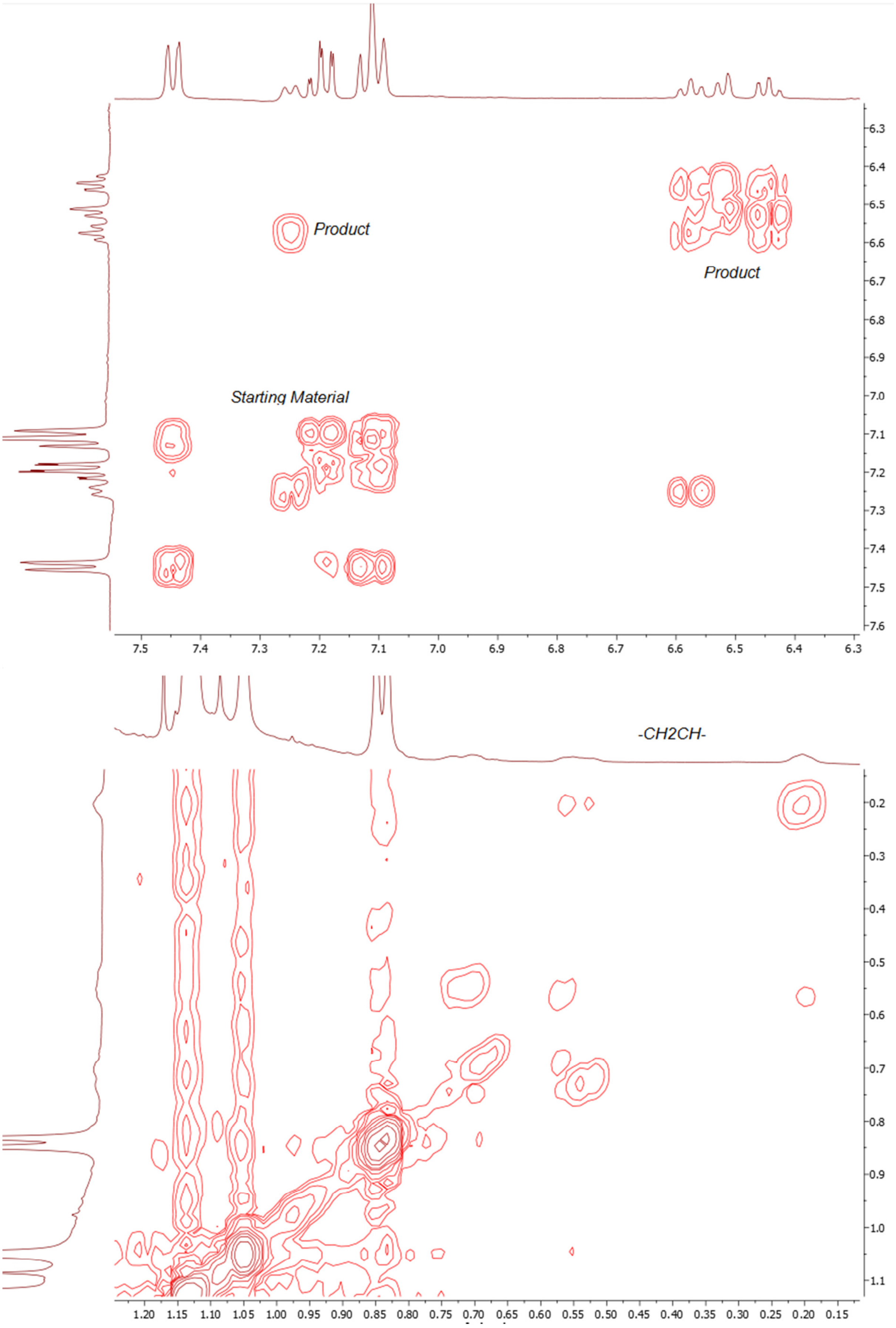

Figure S16. In-situ ^1H-^1H COSY-NMR spectrum of **[5]**$^-$ in THF.

3.6 Addition of 9-*p*-anisyl-BBN

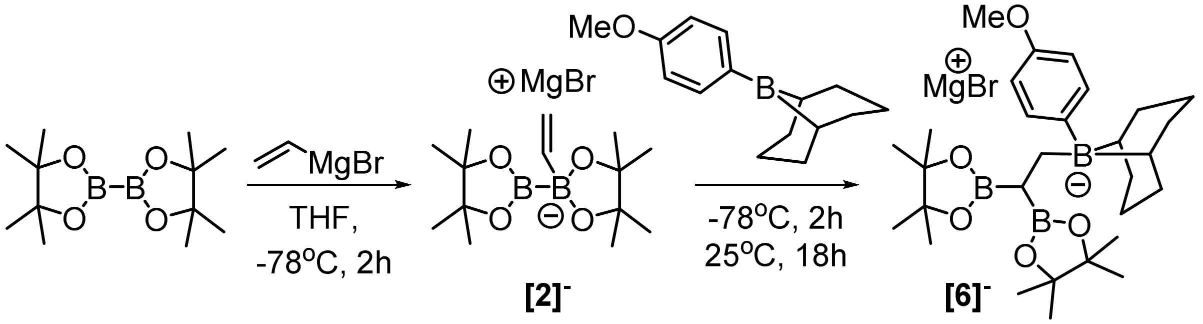

In a J. Young NMR tube, B_2Pin_2 (30 mg, 0.113 mmol, 1.0 eq.) was dissolved in dry THF (0.5 mL) and the solution was then cooled down to -78°C. After 5 min, a 1 M solution of vinyl magnesium bromide (114 µL, 0.113 mmol, 1.0 eq.) was added. The solution was kept at -78°C for 2 hours, with the J. Young NMR tube inverted each 30 min. While still at -78°C, 9-*p*-anisyl-BBN (26 mg, 0.113 mmol, 1.0 eq.) was added to the solution. The solution was kept at -78°C for 2 hours, with the J. Young NMR tube inverted each 30 min. The sample was left warming to room temperature and then was monitored by multi-nuclear NMR spectroscopy, revealing limited formation of the desired product **[6]**$^-$ ($^{11}B\{^1H\}$-NMR signals at 33.9 ppm [-BPin moieties] and -16.0 ppm [R(Ph)BBN$^-$], while the $-CH_2CH-$ appeared as broad signals at 0.42 ppm and 0.24 ppm). The J. Young NMR tube was then left at room temperature for 18 hours and periodically inverted. After this time, more product had formed but the **[2]**$^-$ adduct and 9-Anisyl-BBN were still the major species. Mesitylene addition (10 µL, 0.070 mmol, 0.62 eq.) allowed the determination of the in-situ yield by the relative integration of the aromatic signal of mesitylene and the aromatic ones of **[6]**$^-$ (NMR yield = 15%). Due to the overlap of the BBN resonances of starting materials and product, only diagnostic peaks are reported.

Diagnostic peaks: ^1H-NMR (400 MHz, THF) = 7.13 ppm (d, 2H, -Anisyl), 6.42 ppm (d, 2H, -Anisyl), 0.98 ppm (s, 12H, 4 $-CH_3$), 0.96 ppm (s, 12H, 4 $-CH_3$), 0.42 ppm (brs, 2H, $-CH_2-$), 0.24 ppm (brs, 1H, -CH-). $^{11}B\{^1H\}$-NMR (128 MHz, THF) = 33.91 ppm (brs, 2B, RBPin), -15.98 ppm (s, 1B, R(Anisyl)BBN). Accurate mass for the anion **[6]**$^-$ ($[C_{29}H_{48}B_3O_5]^-$): 509.3776 (expected: 509.3786).

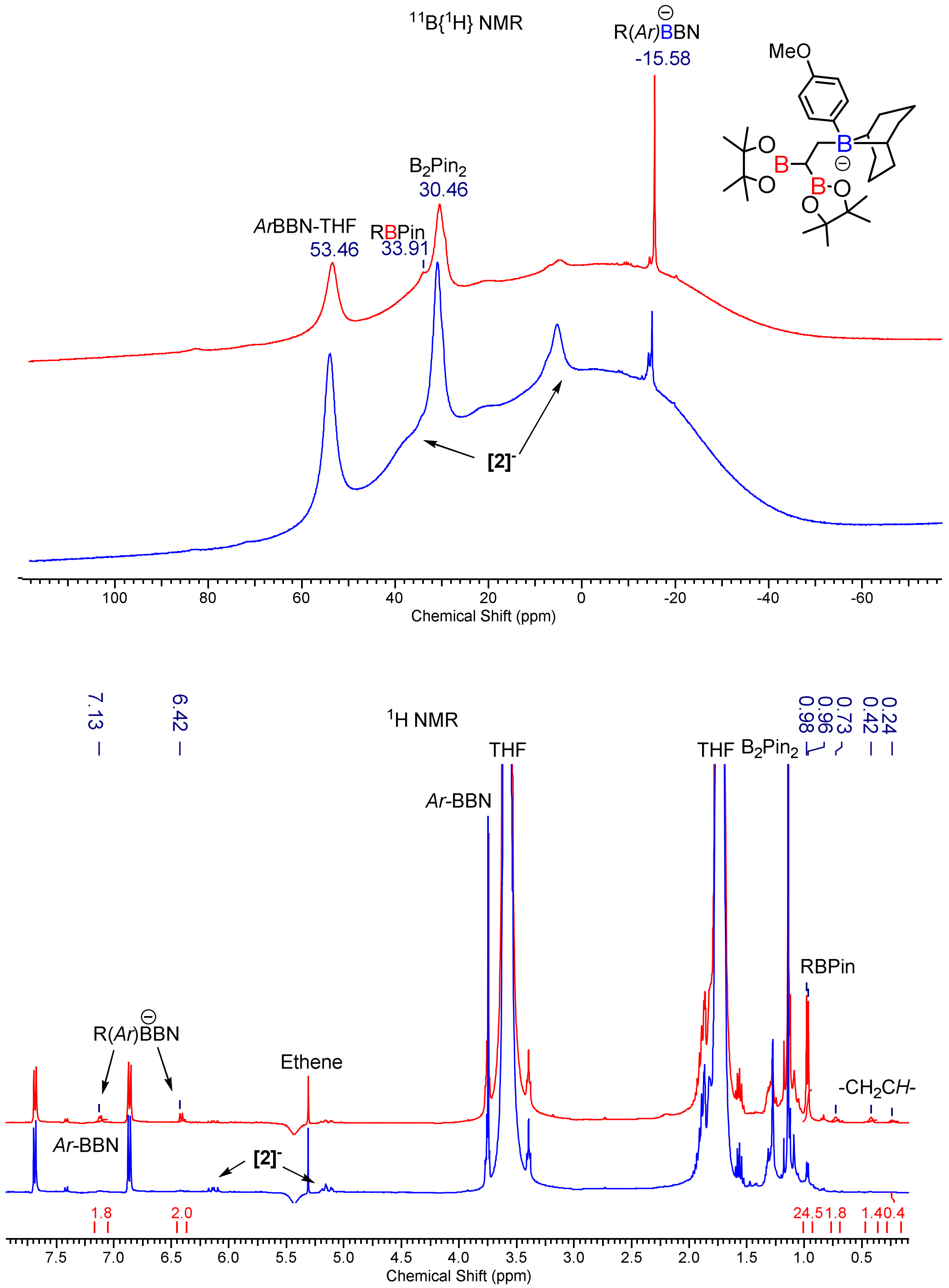

Figure S17. In-situ ^{1}H and $^{11}B\{^{1}H\}$-NMR spectra of an equimolar mixture of B_2Pin_2, vinylMgBr and 9-*p*-anisyl-BBN in dry THF. Blue (after 2 hours at -78°C and 10 mins at RT), red (after 18 hours at RT).

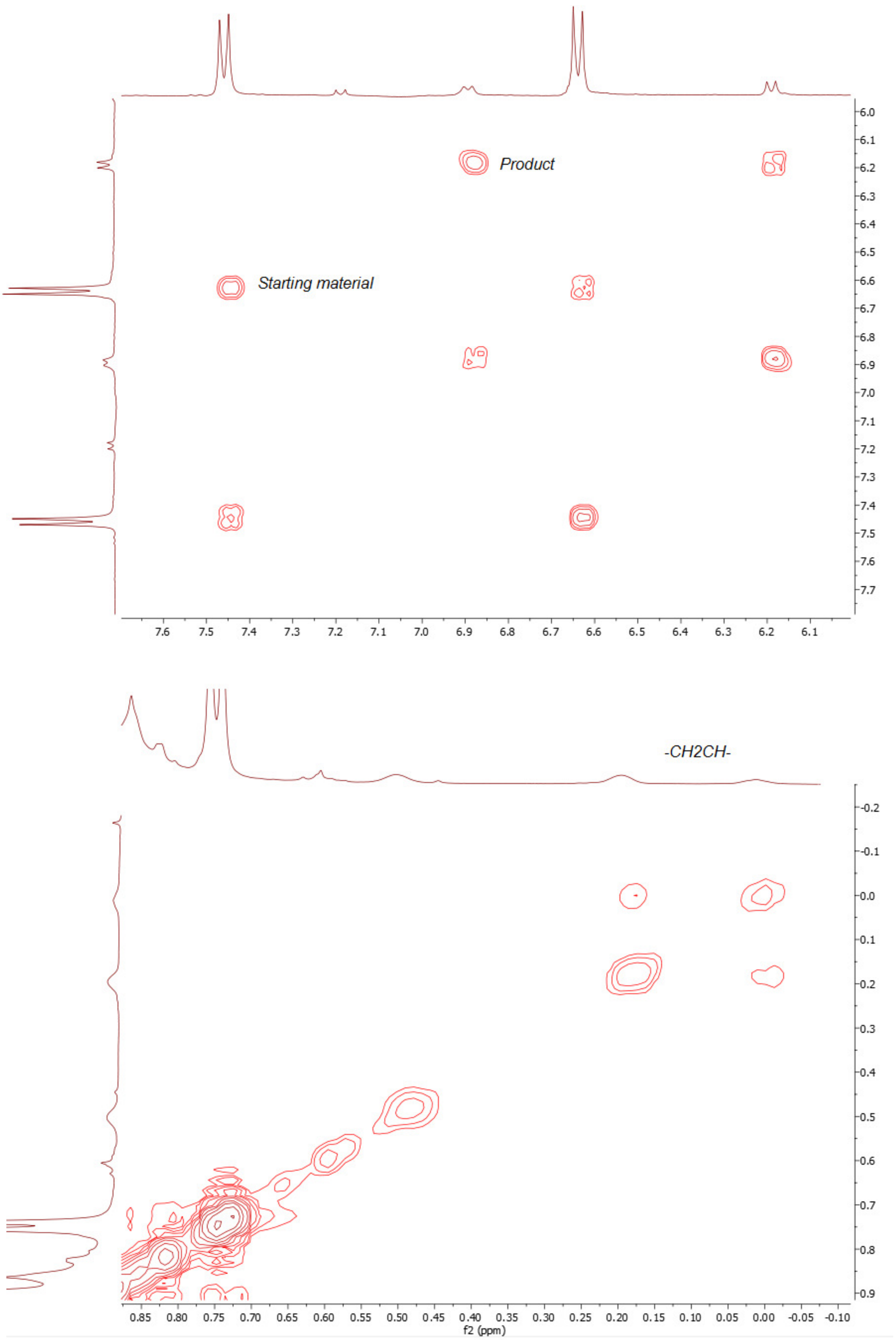

Figure S18. In-situ ^{1}H-^{1}H COSY-NMR spectrum of **[6]**$^{-}$ in dry THF.

4. Addition of BPh_3 to [7]$^-$

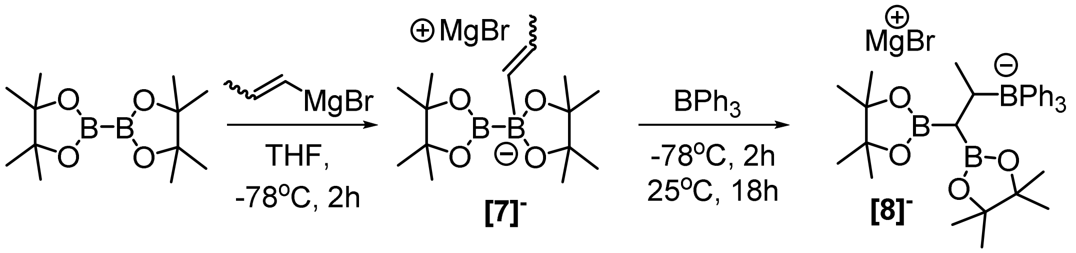

In a J. Young NMR tube, B_2Pin_2 (20 mg, 0.076 mmol, 1.0 eq.) was dissolved in dry THF (0.3 mL) and the solution was then cooled down to -78°C. After 5 min, a 0.5 M THF solution of (E/Z)-1-propenyl magnesium bromide (151 µL, 0.076 mmol, 1.0 eq.) was added, leaving the solution at -78°C for 2 hours. The sample was left warming to room temperature and monitored by multi-nuclear NMR spectroscopy. As for the adduct **[2]**$^-$, signals for tri- and tetra-coordinated the sp^2-sp^3 boron centres in diborane **[7]**$^-$ were observed at 37 ppm and 5-8 ppm, respectively. In this case, as the Grignard is a mixture of E-Z isomers, two different adducts of **[7]**$^-$ were generated which presumably leads to the two observed resonances for the four coordinate borons.

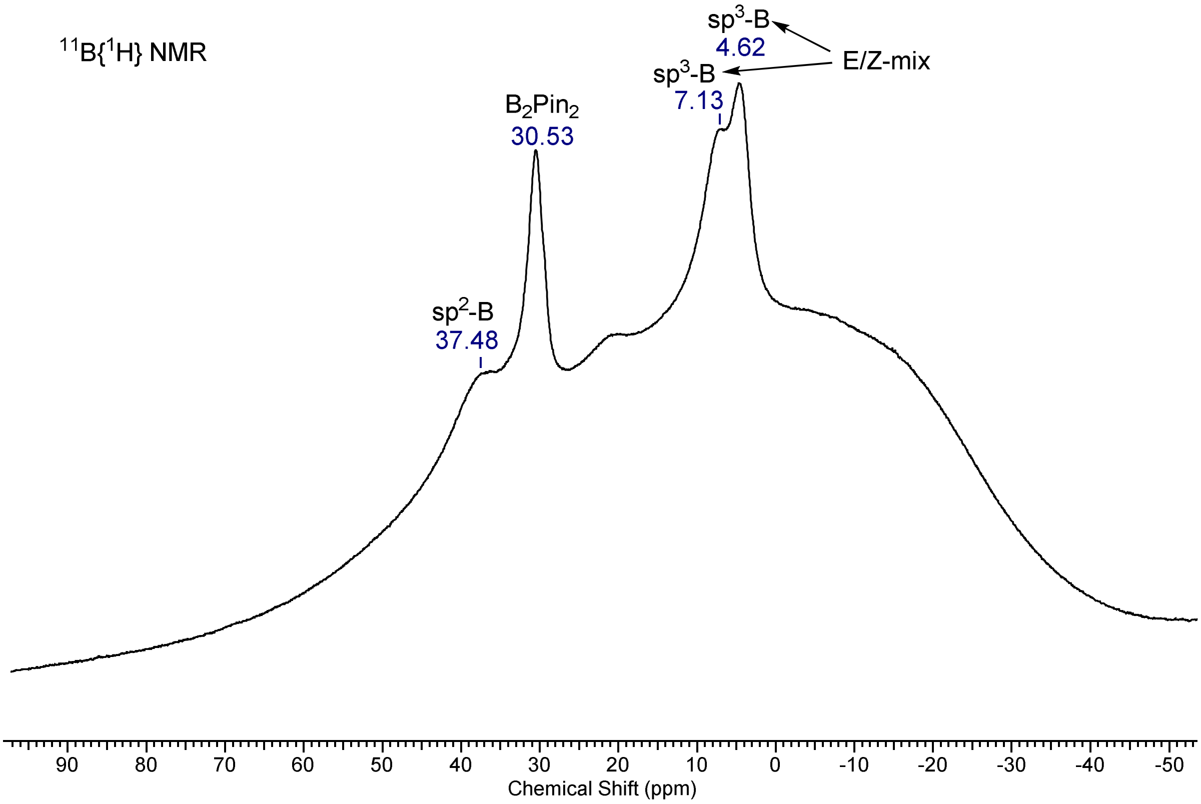

Figure S19. In-situ $^{11}B\{^1H\}$-NMR spectra of an equimolar mixture of B_2Pin_2 and (E/Z)-1-propenyl Grignard in dry THF, after 2 hours at -78°C.

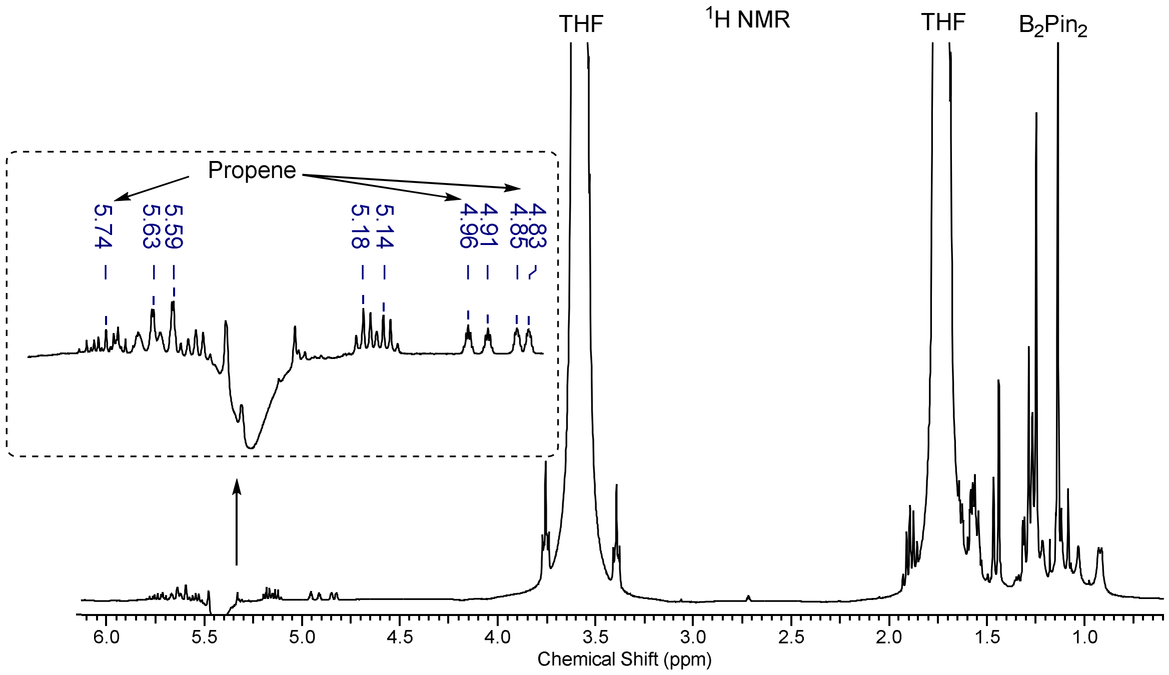

Figure S20. In-situ ^{1}H-NMR spectra of an equimolar mixture of B_2Pin_2 and (E/Z)-1-propenyl Grignard in dry THF, after 2 hours at -78°C and 10 mins at RT.

Then, the solution was cooled to -78°C, followed by the addition of a 0.25 M THF solution of BPh_3 (302 µL, 0.076 mmol, 1.0 eq). The solution was kept at -78°C for 2 hours, with the J. Young NMR tube inverted each 30 min. The sample was left warming to room temperature overnight and then was monitored by multi-nuclear NMR spectroscopy. In the ^{11}B NMR spectrum, there was a single major signal for tetra-coordinate boron-species at -13.8 ppm which was consistent with $[PinB\text{-}BPh_3]^-$; minor four coordinate boron containing products were observed at -9.6/-10.0 ppm due to $[(E/Z)\text{-}1\text{-propenyl-}BPh_3]^-$, respectively, in accordance with the literature and confirmed by independent synthesis. $PhSiMe_3$ addition (10 µL, 0.058 mmol, 0.76 eq.) allowed the determination of the in-situ yield of $[PinB\text{-}BPh_3]^-$ by the relative integration of the methyl signals of $PhSiMe_3$ and the ones of $[PinB\text{-}BPh_3]^-$ (NMR yield ≈ 40%). $[PinB\text{-}BPh_3]^-$ and $[(E/Z)\text{-}1\text{-propenyl-}BPh_3]^-$ were observed in the negative mode of ESI-MS analysis, along with a minor peak for the 1,2-boryla migration product **[8]$^-$**.

$[PinB\text{-}BPh_3]^-$ ^{1}H NMR (400 MHz, THF): 7.20 ppm (d, J = 6.8 Hz, 6H), 6.75 ppm (t, J = 7.3 Hz, 6H), 6.59 ppm (t, J = 7.3 Hz, 3H), 1.03 ppm (s, 12H). ^{11}B{^{1}H} NMR (128 MHz, THF): -13.76 ppm (as reported in the literature,[3] the boron signal for the tricoordinated boron of $[PinB\text{-}BPh_3]^-$ is too broad to be observed in the ^{11}B NMR spectrum). On removing THF and redissolving in dry toluene, the ^{11}B{^{1}H} NMR signal of $[PinB\text{-}BPh_3]^-$ appeared at -14.5 ppm, in analogy with the value reported in the literature (-14.6 ppm, although this is with a different cation (β-diketiminateMg,[3] whereas herein it will be THF_nMgX which may explain the slight difference). Accurate mass for the anion $[PinB\text{-}BPh_3]^-$ ($[C_{24}H_{27}B_2O_2]^-$): 369.2194 (expected: 369.2203).

Accurate mass for the anion [(E/Z)-1-propenyl-BPh_3]$^-$ ($[C_{21}H_{20}B]^-$): 283.1655 (exp: 283.1664). Accurate mass for the anion **[8]$^-$** ($[C_{33}H_{44}B_3O_4]^-$): 537.3507 (exp: 537.3524). In the ^{11}B NMR spectrum, the other signal of a tri-coordinate boron species at 29.33 ppm was assigned (E/Z)-1-propenyl-BPin the expected by-product from intermolecular {BPin} transfer, as confirmed by GC-MS analysis (R_t = 6.101 min, $[M]^+$ = 168.1 m/z; R_t = 6.189 min, $[M]^+$ = 168.1 m/z).

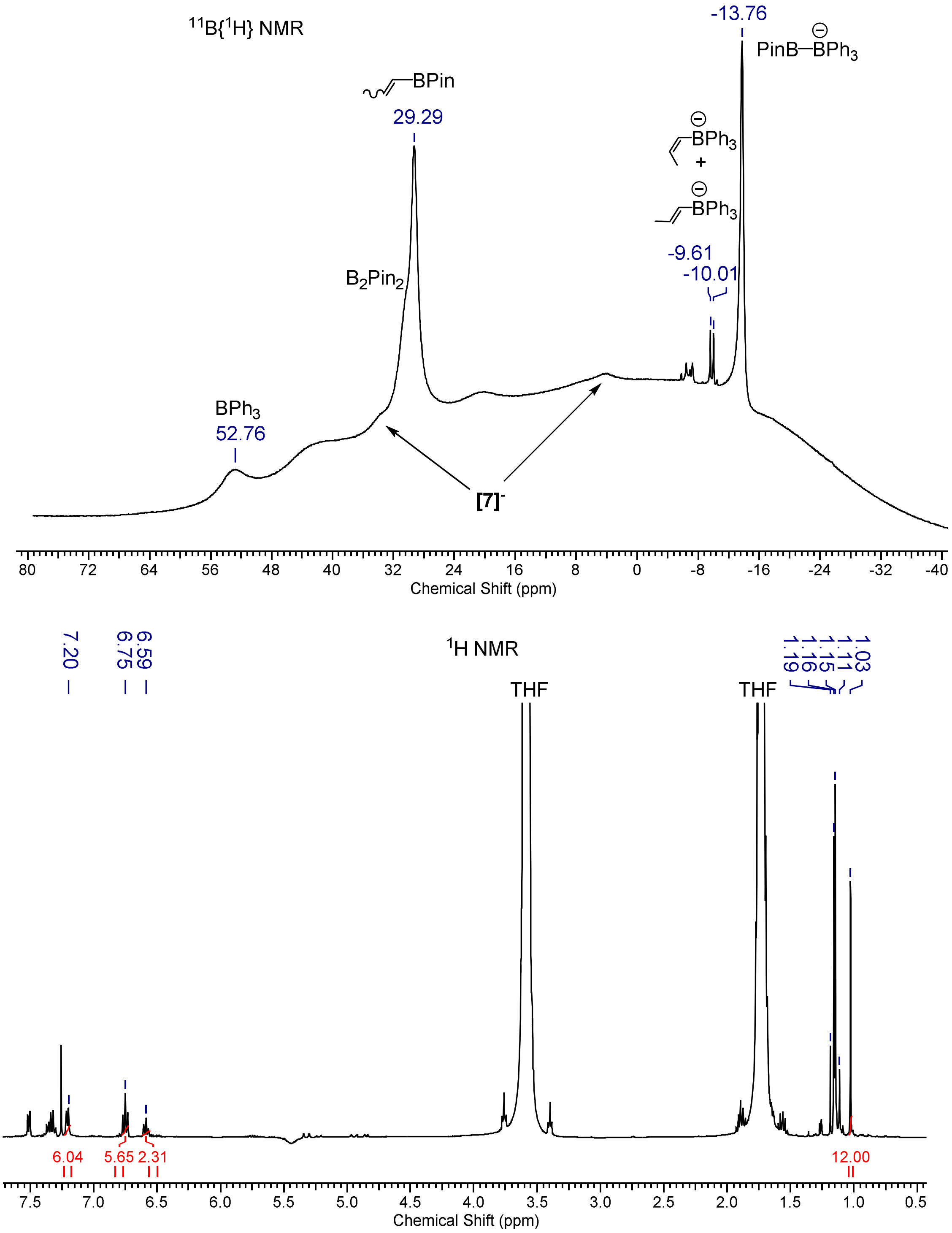

Figure S21. In-situ ^1H and $^{11}B\{^1H\}$-NMR of the reaction employing an equimolar mixture of B_2Pin_2, 1-propenyl magnesium bromide, and BPh_3 (after 18 hr at RT).

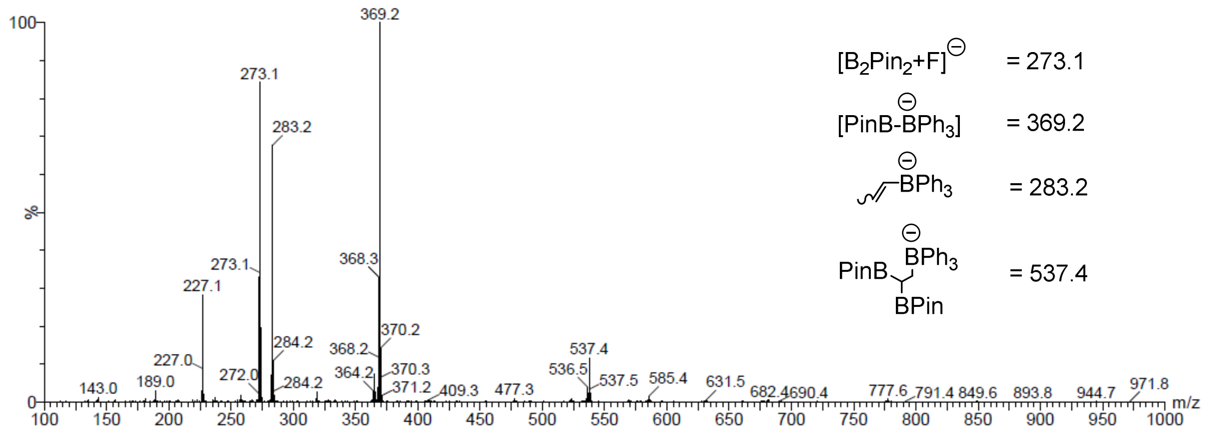

Figure S22. ESI (negative mode) of the reaction employing an equimolar mixture of B_2Pin_2, 1-propenyl magnesium bromide, and BPh_3 (after 18 hr at RT).

5. Addition of $HNTf_2$ to [4][MgBr(THF)$_2$]

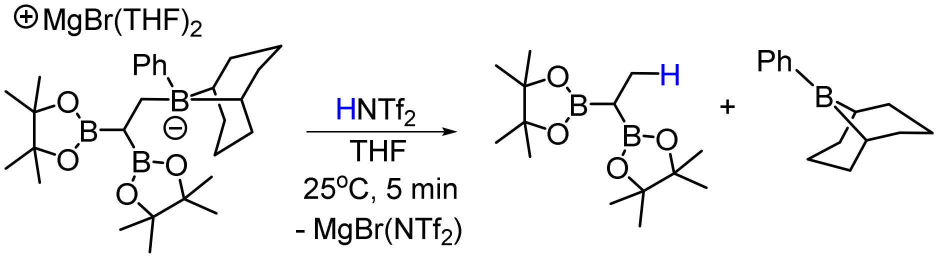

[4][MgBr(THF)$_2$]

In a J. Young NMR tube, **[4][MgBr(THF)$_2$]** (15.0 mg, 0.020 mmol, 1.0 eq.) was dissolved in dry THF (0.5 mL), followed by the addition of $HNTf_2$ (6.0 mg, 0.020 mmol, 1.0 eq.). Multi-nuclear NMR spectroscopy revealed mainly formation of 9-Ph-BBN (77%) and (PinB)$_2$CHCH$_3$ (60%), along with other minor products such as benzene (14%) and a signal at 22.12 ppm in ^{11}B NMR (in-situ yield calculated by adding mesitylene - 3 µL, 1.06 eq. - as internal standard). (PinB)$_2$CHCH$_3$ ^{1}H-NMR (400 MHz, THF) = 1.13 ppm (s, 24H), 0.90 ppm (d, J = 7.28 Hz, 3H), 0.50 ppm (q, J = 7.28 Hz, 1H). ^{11}B{^{1}H}-NMR (128 MHz, THF) = 33.80 ppm. GC-MS: R_t = 9.083 min, $[M]^+$ = 282.1 m/z. Accurate Mass $[M+K]^+$ $C_{14}H_{28}B_2O_4K$: 321.1792 (exp: 321.1805). The data are in agreement to those reported in the literature.[4]

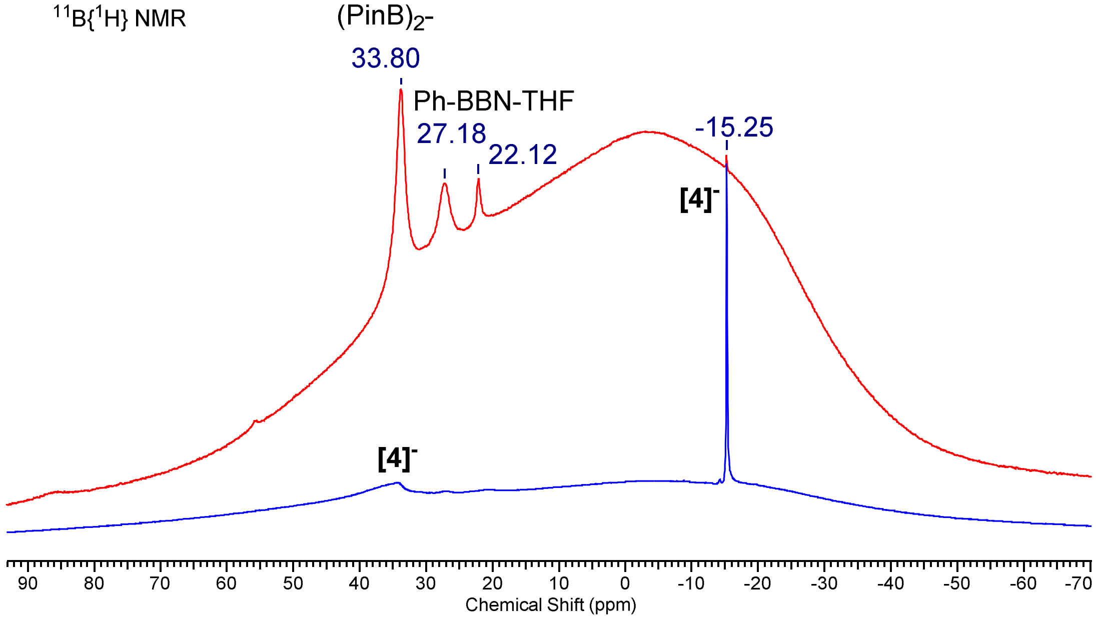

Figure S23. In-situ ^{11}B{^{1}H}-NMR spectra of an equimolar mixture of **[4][MgBr(THF)$_2$]** and $HNTf_2$ in dry THF. Blue (before acid addition), red (after 5 min at RT).

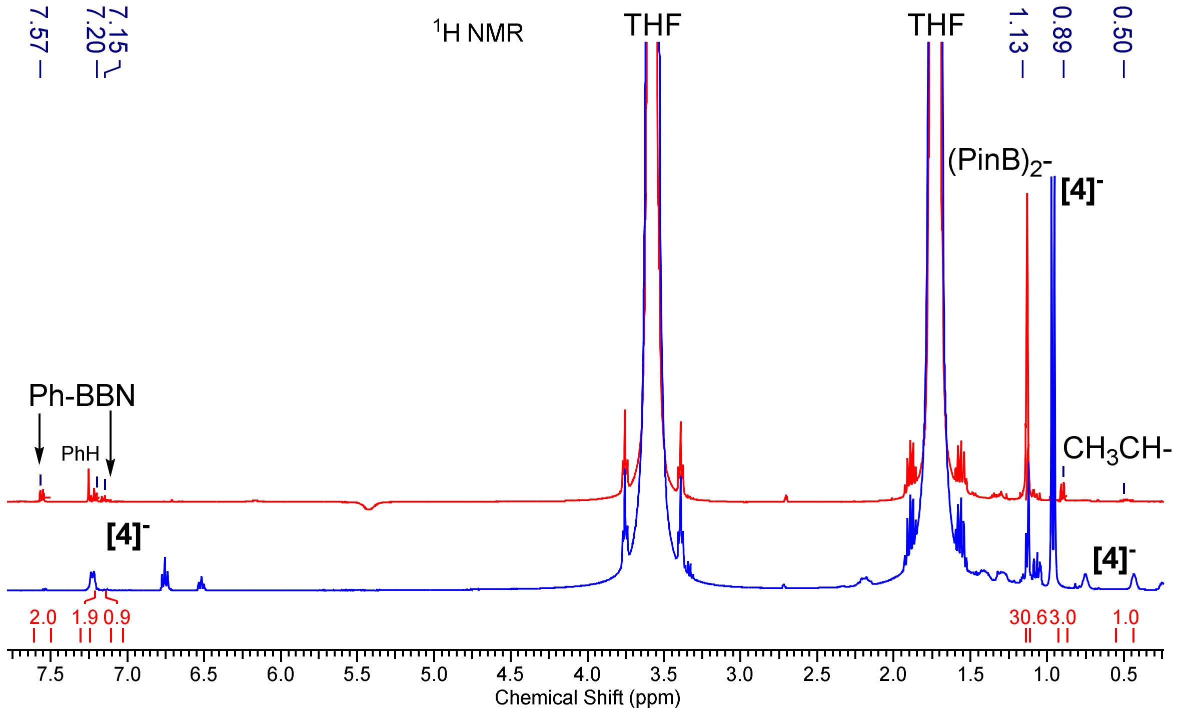

Figure S24. In-situ ^{1}H-NMR spectra of an equimolar mixture of **[4][MgBr(THF)$_2$]** and $HNTf_2$ in dry THF. Blue (before acid addition), red (after 5 min at RT).

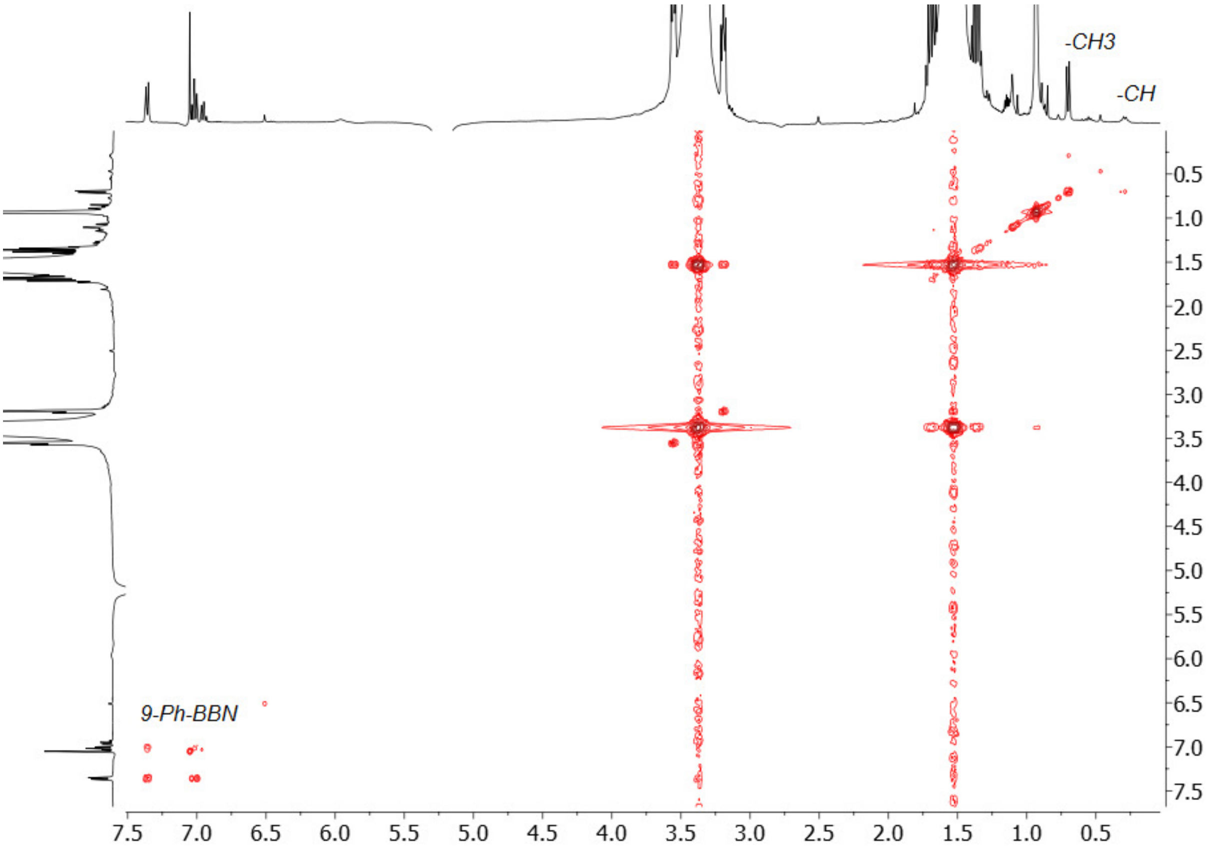

Figure S25. In-situ COSY NMR spectrum of an equimolar mixture of **[4][MgBr(THF)$_2$]** and $HNTf_2$ in dry THF (after 5 min at RT).

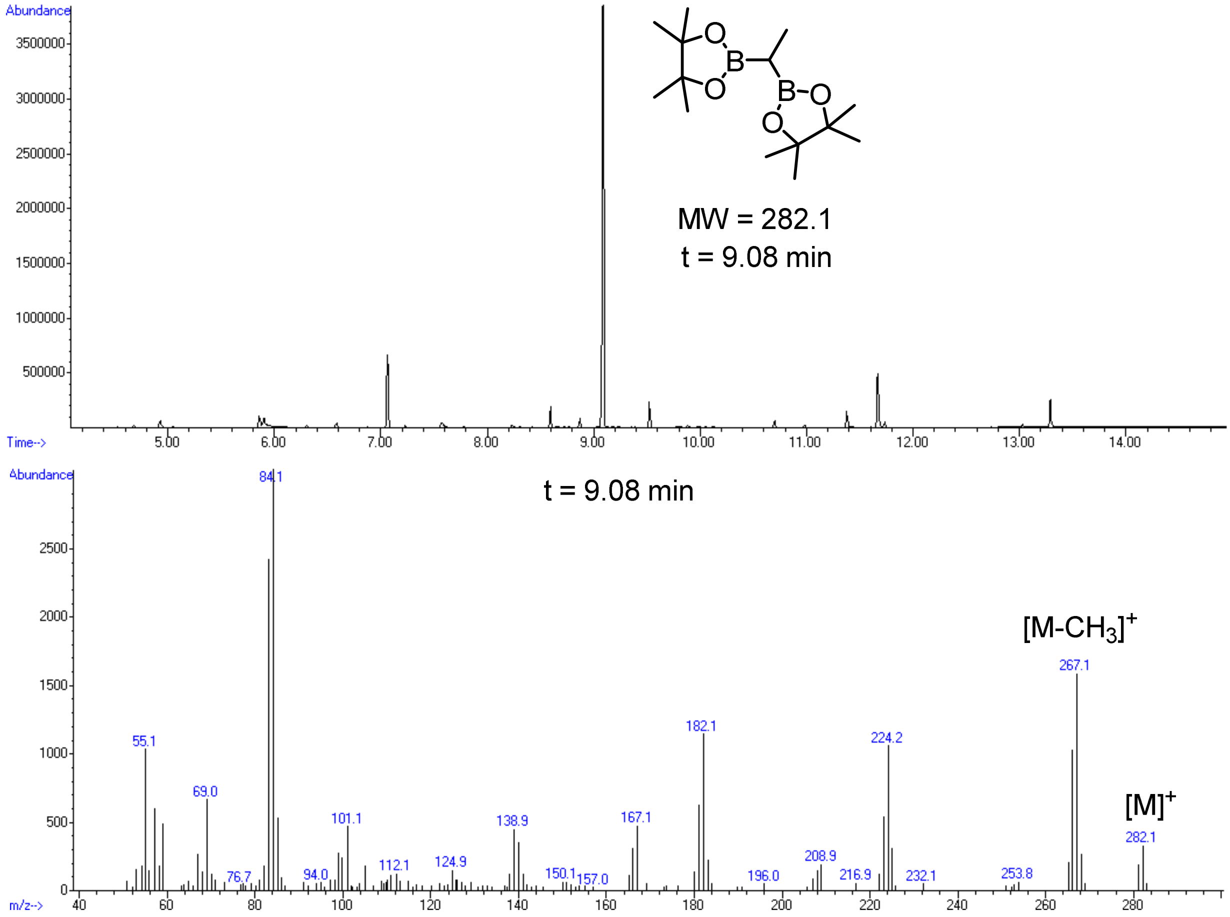

Figure S26. GC-MS analysis of the reaction mixture **[4][MgBr(THF)$_2$]** : $HNTf_2$.

6. Addition of $HNTf_2$ to $[2]^-$

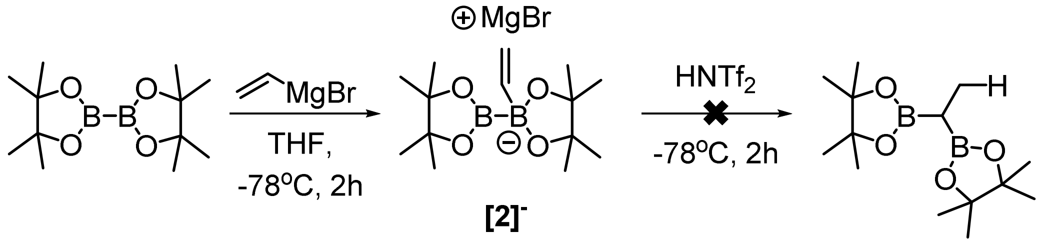

In a J. Young NMR tube, B_2Pin_2 (30 mg, 0.113 mmol, 1.0 eq.) was dissolved in dry THF (0.5 mL) and the solution was then cooled down to -78°C. After 5 min, a 1 M solution of vinyl magnesium bromide (114 µL, 0.113 mmol, 1.0 eq.) was added. The solution was kept at -78°C for 2 hours, with the J. Young NMR tube inverted each 30 min. While still at -78°C, a solution of $HNTf_2$ (34 mg, 0.113 mmol, 1.0 eq.) in THF (0.2 mL) was added to the solution. The solution was kept at -78°C for 2 hours, with the J. Young NMR tube inverted each 30 min. The sample was left warming to room temperature and analysed by multinuclear NMR spectroscopy, revealing no **$[2]^-$** remaining. ^1H and $^{11}B\{^1H\}$-NMR spectra revealed formation of ethene and B_2Pin_2 as major products. Additional boron resonances at 46.0 and 29.8 ppm in the ^{11}B NMR spectrum, along with vinyl resonances in the ^1H NMR spectrum may suggest minor pinacol opening and intermolecular BPin transfer. GC-MS analysis confirmed B_2Pin_2 as major product, along with minor amounts of 1,1-diboryl-ethane and vinyl-BPin.

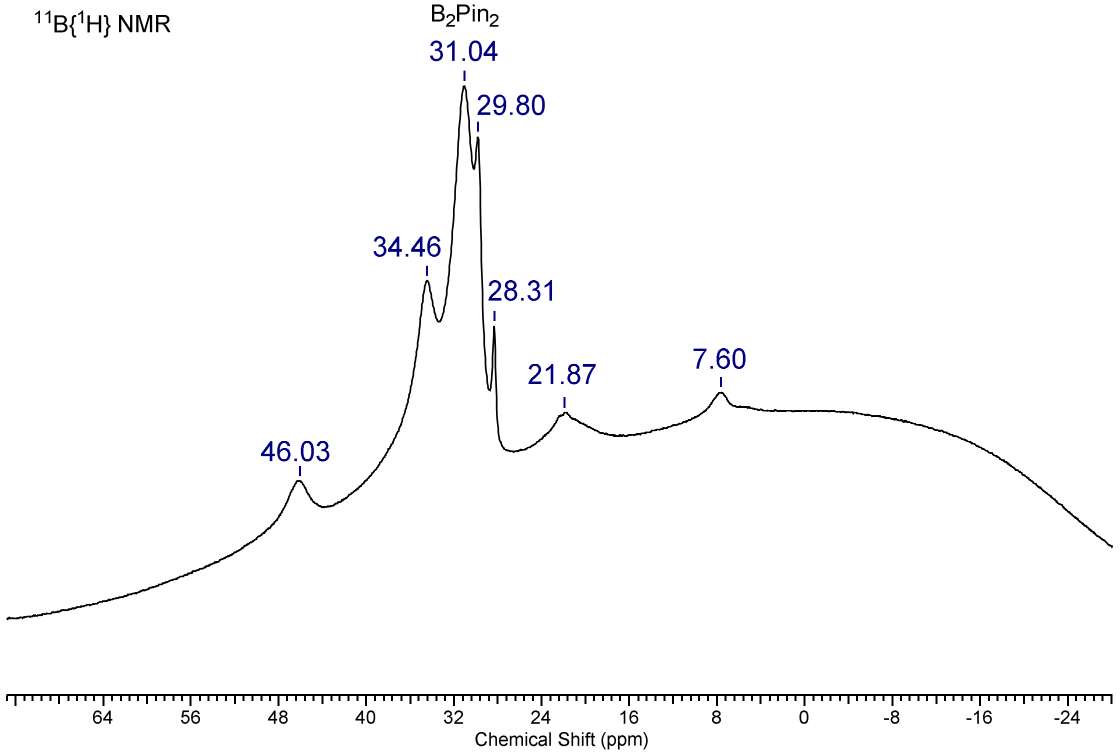

Figure S27. In-situ $^{11}B\{^1H\}$-NMR spectra of an equimolar mixture of B_2Pin_2, vinylMgBr and $HNTf_2$ in dry THF, after 2 hours at -78°C.

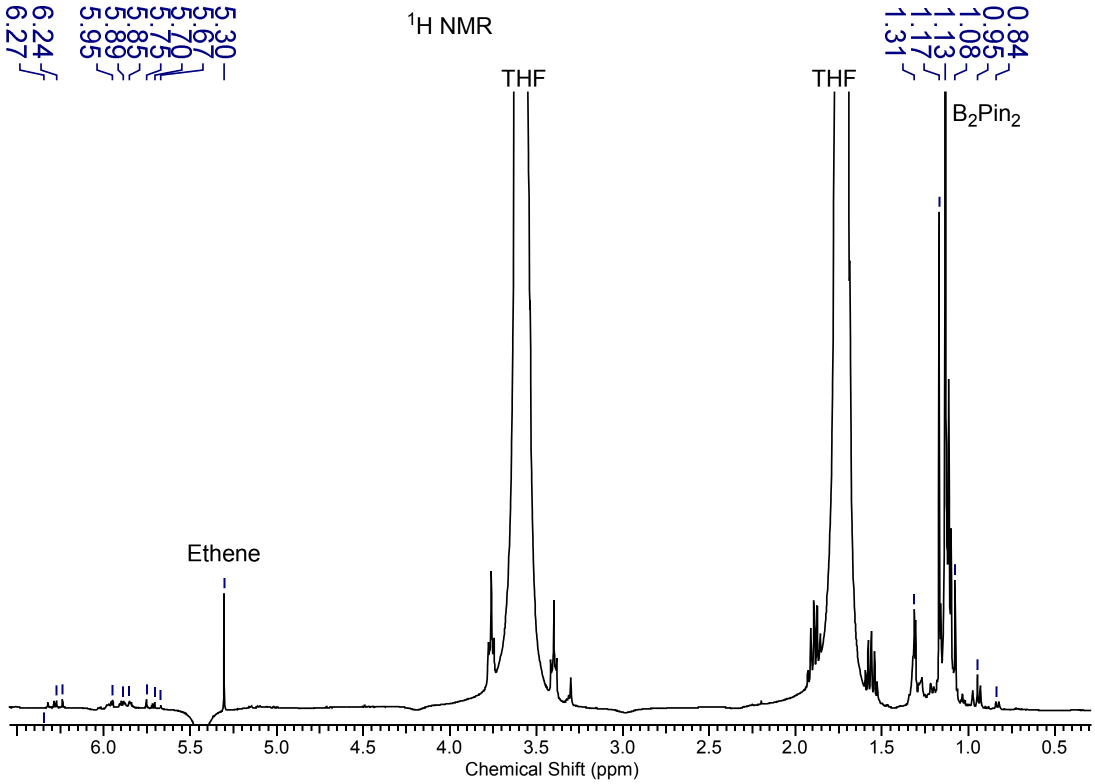

Figure S28. In-situ ^{1}H-NMR spectra of an equimolar mixture of B_2Pin_2, vinylMgBr and $HNTf_2$ in dry THF, after 2 hours at -78°C.

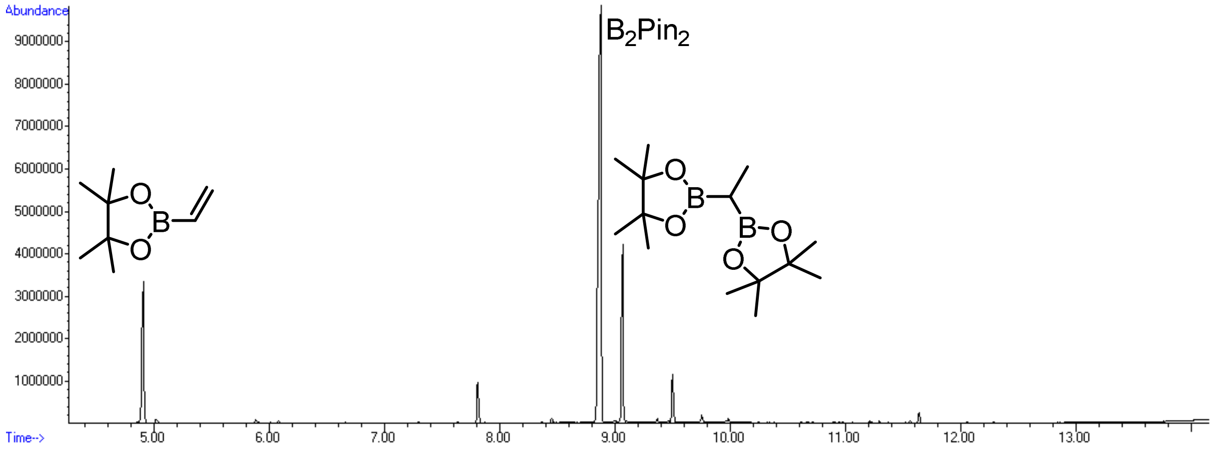

Figure S29. GC-MS analysis of the reaction mixture **[2]**$^-$ + $HNTf_2$.

7. Computational data

Calculations were performed using the Gaussian09[5] suite of programmes. Geometries were optimized with the DFT method using M06-2X functional and 6-311G(d,p) as a basis set, with PCM (Tetrahydrofuran) solvation.[6] All geometry optimizations were full, with no restrictions. All stationary points located in the potential energy hypersurface were characterized as minima (no imaginary frequencies) or as transition states (one and only one imaginary frequency) by vibrational analysis. The analysis also provided zero-point vibrational energy corrections and thermal corrections to various thermodynamic properties. The transition state was further confirmed by IRC calculations (calcALL, forward, maxpoints=40, stepsize=20 / (calcALL, reverse, maxpoints=40, stepsize=20). Full Cartesian coordinates for the optimised geometries are reported below.

BPh_3

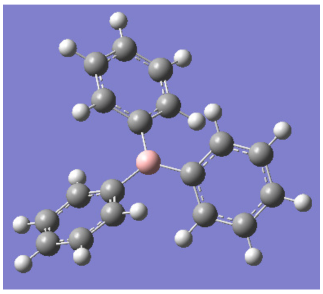

B	-0.136200	0.861600	0.007700
C	0.865500	0.966900	-1.191400
C	0.622600	0.297900	-2.403000
C	2.042800	1.728300	-1.096100
C	1.503000	0.395600	-3.474300
H	-0.278400	-0.297400	-2.509100
C	2.944500	1.803400	-2.151600
H	2.259900	2.257700	-0.174100
C	2.671200	1.141900	-3.345800
H	1.285300	-0.115400	-4.404900
H	3.855500	2.381300	-2.048400
H	3.366700	1.208900	-4.174400
C	-0.339600	2.083000	0.965600
C	-0.224400	3.401200	0.492300
C	-0.646900	1.907800	2.325600
C	-0.423500	4.492600	1.329700
H	0.011700	3.571300	-0.553000
C	-0.817500	2.994200	3.175800
H	-0.741400	0.902900	2.724000
C	-0.713800	4.289700	2.676000
H	-0.346100	5.499800	0.937000

H	-1.037900	2.833300	4.224700
H	-0.858900	5.138200	3.334700
C	-0.934200	-0.465000	0.241700
C	-2.225900	-0.454300	0.794600
C	-0.384900	-1.713600	-0.096000
C	-2.941500	-1.630400	0.988200
H	-2.681300	0.493100	1.063500
C	-1.082700	-2.896000	0.121800
H	0.611100	-1.757700	-0.524200
C	-2.366500	-2.854600	0.658500
H	-3.943200	-1.594800	1.400300
H	-0.630900	-3.847900	-0.131900
H	-2.917100	-3.774300	0.819100

$B(C_6F_5)_3$

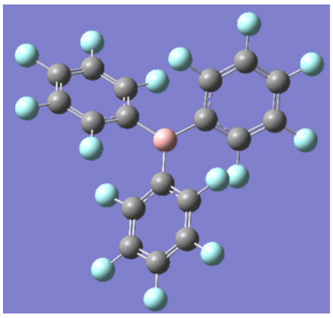

B	-0.147900	0.855700	0.004400
C	0.859600	0.964200	-1.190300
C	0.621300	0.353900	-2.420800
C	2.053200	1.677500	-1.086200
C	1.498900	0.445100	-3.485000
C	2.963400	1.770800	-2.122600
C	2.679500	1.153300	-3.329800
C	-0.351400	2.082000	0.956500
C	-0.277000	3.395200	0.491000
C	-0.608400	1.935800	2.319900
C	-0.457200	4.493000	1.311000
C	-0.774400	3.011700	3.171700
C	-0.702500	4.297300	2.660100
C	-0.942000	-0.472300	0.239900
C	-2.233200	-0.484100	0.769500
C	-0.403000	-1.722400	-0.068000
C	-2.948700	-1.648300	0.977500
C	-1.085000	-2.906500	0.141100
C	-2.366900	-2.865700	0.663900
F	-0.042200	3.644000	-0.797700
F	-0.395000	5.726400	0.822400

F	-0.866300	5.337500	3.459300
F	-1.001600	2.828100	4.467300
F	-0.683200	0.721900	2.865800
F	-2.844300	0.657900	1.085100
F	-4.178800	-1.613200	1.476700
F	-3.035100	-3.988600	0.864000
F	-0.528100	-4.075500	-0.153900
F	0.828200	-1.823800	-0.569000
F	-0.498200	-0.342700	-2.621800
F	1.225800	-0.139200	-4.646000
F	3.535200	1.241100	-4.334400
F	4.098200	2.445200	-1.975200
F	2.373600	2.292500	0.053100

BF_3

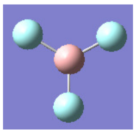

B	-0.136100	0.861500	0.006400
F	-0.307300	1.884600	0.810700
F	-0.805900	-0.249900	0.205000
F	0.704200	0.950400	-0.998100

VinylMgBr(THF)$_2$

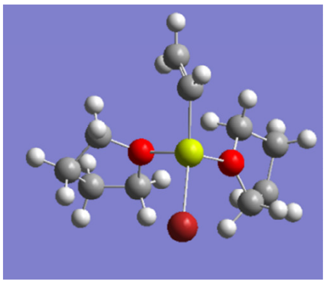

Mg	0.409300	4.102400	-0.422900
O	-0.529800	5.910700	-0.122900
C	0.246800	7.100800	0.165200
C	-1.732200	6.260900	-0.851900
C	-0.641300	8.267000	-0.251400
H	0.497500	7.095900	1.226900
H	1.161800	7.053900	-0.431300
C	-1.457300	7.654000	-1.391800
H	-1.893200	5.500700	-1.615400
H	-2.570600	6.252200	-0.151300
H	-0.056300	9.134100	-0.554700
H	-1.299400	8.557800	0.570300

H	-0.854800	7.593400	-2.301200
H	-2.374500	8.199600	-1.609700
O	1.418700	4.479800	1.311200
C	0.696300	4.424900	2.566100
C	2.759400	4.993100	1.511400
C	1.770200	4.577300	3.631500
H	-0.019300	5.253300	2.582200
H	0.152800	3.481700	2.595900
C	2.765200	5.514100	2.942300
H	3.460400	4.168000	1.371500
H	2.945200	5.755800	0.755600
H	2.236800	3.611300	3.836800
H	1.370400	4.979300	4.561400
H	3.761500	5.484000	3.381100
H	2.401000	6.543800	2.975300
Br	2.124600	4.660000	-2.146100
C	-1.166000	2.721100	-0.115400
H	-1.254200	1.703200	-0.510800
C	-2.203600	3.078600	0.656500
H	-2.260100	4.064900	1.123400
H	-3.055000	2.429900	0.877500

B_2Pin_2

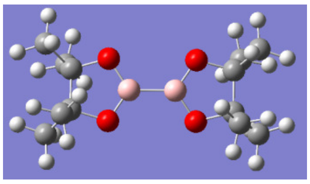

C	0.846400	1.206300	-2.214000
C	0.286800	2.652200	-2.395600
O	1.925000	1.173200	-3.184400
O	1.459100	3.376900	-2.852200
B	2.317500	2.466300	-3.407900
C	5.722700	2.673700	-5.328300
C	5.289400	4.172600	-5.303900
B	3.723400	2.890500	-4.262600
O	4.220700	4.165100	-4.322100
O	4.494000	1.995600	-4.956800
C	-0.232100	3.300200	-1.124300
H	-0.606700	4.299500	-1.353000
H	-1.053900	2.712500	-0.707100
H	0.553300	3.389500	-0.374600
C	-0.752100	2.751700	-3.508900
H	-1.690500	2.275900	-3.217200

H	-0.944400	3.805800	-3.715000
H	-0.389200	2.281800	-4.425500
C	1.483300	0.984400	-0.845000
H	0.727100	0.936500	-0.059000
H	2.030000	0.040300	-0.862100
H	2.186700	1.786000	-0.609000
C	-0.139200	0.095700	-2.530100
H	0.342000	-0.871600	-2.374900
H	-1.006300	0.157800	-1.867500
H	-0.480700	0.149200	-3.563200
C	6.184200	2.164600	-6.682000
H	7.058500	2.727900	-7.019100
H	6.465100	1.113300	-6.598500
H	5.398200	2.253100	-7.431100
C	4.671100	4.634000	-6.620600
H	4.206400	5.609000	-6.466000
H	5.428300	4.728200	-7.401700
H	3.902300	3.935600	-6.958100
C	6.750100	2.336500	-4.251400
H	6.852400	1.251700	-4.191900
H	7.725500	2.767600	-4.485600
H	6.426500	2.706400	-3.276100
C	6.368500	5.141400	-4.854200
H	7.229000	5.090000	-5.526300
H	5.975700	6.159400	-4.877500
H	6.700300	4.923000	-3.839700

2′

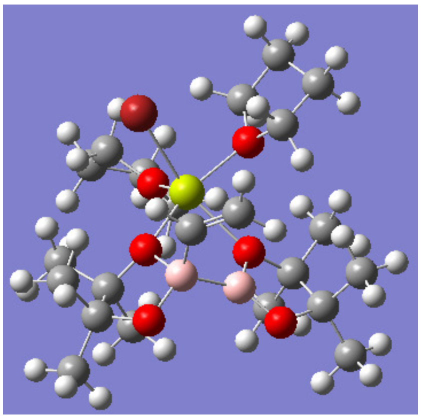

C	-1.568500	0.351000	0.893700
C	-1.941000	1.800300	0.465000
O	-0.266700	0.184000	0.356800
O	-0.707600	2.470900	0.723600
C	1.564600	1.655600	1.523800
H	1.257400	1.556100	2.568400

C	2.851900	1.940900	1.307900
H	3.245800	2.046200	0.296800
H	3.578600	2.061000	2.107900
B	0.444300	1.472200	0.369300
C	2.135800	2.717500	-3.053300
C	1.162700	3.867700	-2.653600
B	1.040200	2.106000	-1.121800
O	0.865200	3.515600	-1.265400
O	1.679000	1.620000	-2.227600
C	-3.054400	2.448400	1.272800
H	-3.305700	3.418600	0.835000
H	-3.954100	1.827200	1.253300
H	-2.750100	2.599000	2.309500
C	-2.256000	1.888600	-1.028900
H	-3.250400	1.493800	-1.249500
H	-2.225100	2.938200	-1.335200
H	-1.521800	1.329600	-1.614000
C	-1.521000	0.237300	2.421400
H	-2.523400	0.216700	2.857600
H	-1.007200	-0.689200	2.685300
H	-0.968500	1.078300	2.846700
C	-2.482900	-0.719600	0.315100
H	-2.179000	-1.697800	0.694200
H	-3.523300	-0.548200	0.605600
H	-2.416100	-0.741200	-0.773100
C	2.055200	2.300600	-4.511900
H	2.297100	3.143100	-5.164900
H	2.778800	1.505000	-4.697200
H	1.063300	1.928800	-4.765000
C	-0.153200	3.798700	-3.421400
H	-0.867800	4.492100	-2.978000
H	-0.009400	4.078800	-4.466600
H	-0.578100	2.794100	-3.380900
C	3.584200	3.000600	-2.666100
H	4.156300	2.077000	-2.765500
H	4.027500	3.758900	-3.314500
H	3.656700	3.336500	-1.629700
C	1.760300	5.259100	-2.747500
H	2.019900	5.468700	-3.788500
H	1.035300	6.005600	-2.419000
H	2.657600	5.363500	-2.140600
Mg	0.038100	4.292000	0.599100
O	-1.608900	5.471900	0.259800
C	-2.449700	6.002200	1.326300

C	-2.035600	6.005200	-1.017800
C	-3.651700	6.582200	0.604200
H	-2.668500	5.189100	2.014100
H	-1.883100	6.769600	1.856900
C	-3.013100	7.119100	-0.678100
H	-1.149500	6.340500	-1.559100
H	-2.526300	5.202500	-1.574000
H	-4.146900	7.351700	1.194600
H	-4.374700	5.796600	0.370000
H	-2.476400	8.049000	-0.474800
H	-3.727000	7.291900	-1.482000
O	1.334600	5.850600	0.247500
C	0.971900	7.233300	0.480500
C	2.729400	5.649100	0.573900
C	2.257900	7.926300	0.928400
H	0.592000	7.632200	-0.463900
H	0.189600	7.262800	1.239500
C	3.351400	7.016000	0.361600
H	2.800200	5.329600	1.618000
H	3.101300	4.856900	-0.075300
H	2.314200	7.938600	2.018500
H	2.316200	8.950400	0.562700
H	4.304900	7.120800	0.877100
H	3.500200	7.205700	-0.704600
Br	0.231700	4.776700	3.127100

2′ *O*-coordinating BPh_3

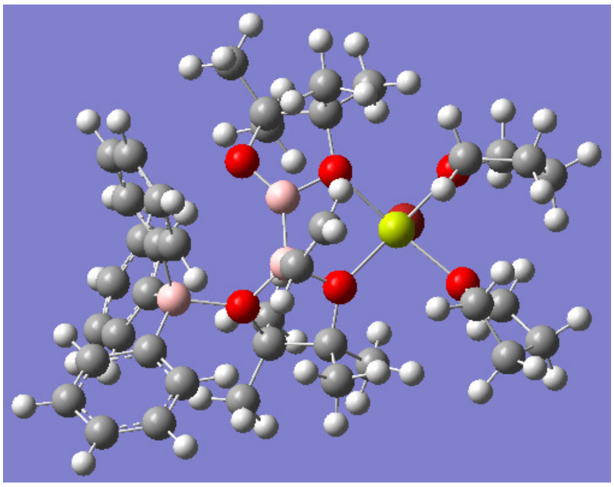

C	2.716700	6.940800	2.415500
C	3.839800	6.185800	3.185900
O	3.245600	6.956900	1.078900
O	4.153100	5.089200	2.246600

B	3.891200	5.585300	0.797500
C	3.372400	5.656300	4.531600
H	4.177300	5.119300	5.030700
H	3.099100	6.508800	5.159200
H	2.514300	4.994800	4.448400
C	5.041900	7.113200	3.380100
H	4.719000	8.018200	3.895900
H	5.805700	6.646700	3.993500
H	5.480200	7.407000	2.422600
C	1.378000	6.202000	2.449200
H	0.918600	6.304100	3.434600
H	0.702500	6.647300	1.717500
H	1.479500	5.145600	2.218900
C	2.485200	8.361600	2.915600
H	1.551300	8.744200	2.504500
H	2.392400	8.367100	4.004000
H	3.295300	9.031700	2.628300
C	2.801900	4.703200	-0.007900
H	2.139200	4.019900	0.526500
C	7.266100	6.430600	-1.158900
C	6.146600	7.087700	-2.011100
B	5.248600	5.973400	-0.168700
O	5.037600	7.077700	-1.044000
O	6.514500	5.507200	-0.334000
C	8.289000	5.641600	-1.956500
H	8.791000	6.287500	-2.681300
H	9.041100	5.239300	-1.275400
H	7.822500	4.808700	-2.481900
C	5.720000	6.202200	-3.175300
H	4.793400	6.588100	-3.600400
H	6.482700	6.204200	-3.956100
H	5.550900	5.173000	-2.850100
C	6.453900	8.496800	-2.477600
H	7.404100	8.495000	-3.018400
H	5.676600	8.844300	-3.158800
H	6.524900	9.191300	-1.640100
C	7.948000	7.423300	-0.222400
H	8.566600	6.868900	0.485800
H	8.590300	8.109700	-0.777900
H	7.211500	8.007700	0.336200
C	2.583200	4.822800	-1.320400
H	1.796900	4.280000	-1.837900
H	3.207400	5.462200	-1.942400
Mg	3.662800	8.427800	-0.262800

O	1.838700	9.377300	0.060200
C	1.576300	10.707500	0.579000
C	0.601100	8.733800	-0.308500
C	0.054700	10.864500	0.547100
H	1.986400	10.752500	1.587900
H	2.100900	11.434600	-0.041800
C	-0.453100	9.418200	0.542200
H	0.415400	8.903400	-1.374100
H	0.722200	7.664800	-0.129800
H	-0.259900	11.372200	-0.367400
H	-0.305800	11.439900	1.398400
H	-1.450500	9.318800	0.116100
H	-0.463400	9.001100	1.551600
O	3.381700	9.078600	-2.228000
C	3.385000	10.475100	-2.581000
C	2.590000	8.385200	-3.216000
C	1.966100	10.730600	-3.059700
H	3.694600	11.038900	-1.702800
H	4.114300	10.626900	-3.383800
C	1.625500	9.430900	-3.810400
H	3.260600	7.989300	-3.980700
H	2.093800	7.555200	-2.711400
H	1.898600	11.612900	-3.694500
H	1.305500	10.871500	-2.202200
H	1.812800	9.543700	-4.877900
H	0.582300	9.142400	-3.684700
Br	5.104900	10.231700	0.717000
C	5.513800	2.952500	1.282600
C	6.843900	2.709300	0.909400
C	4.542100	2.298600	0.508100
C	7.180800	1.925700	-0.193100
H	7.652900	3.131200	1.491200
C	4.857100	1.532400	-0.609200
H	3.501100	2.369300	0.798700
C	6.187300	1.347900	-0.974200
H	8.225700	1.767600	-0.439100
H	4.064300	1.064100	-1.183200
H	6.444800	0.743600	-1.837200
C	4.147700	2.702400	3.460600
C	4.747600	1.628700	4.140000
C	2.751700	2.666100	3.372600
C	4.003100	0.611200	4.728300
H	5.831400	1.578900	4.195100
C	1.989300	1.653700	3.952300

H	2.242800	3.447800	2.821900
C	2.612900	0.621600	4.644400
H	4.508600	-0.196600	5.247000
H	0.908600	1.669300	3.858000
H	2.028200	-0.168900	5.100900
C	6.370500	4.315400	3.481400
C	6.547700	4.134500	4.858800
C	7.352000	5.083500	2.830200
C	7.622100	4.691600	5.553100
H	5.819500	3.558700	5.420800
C	8.430600	5.642200	3.505600
H	7.246300	5.273000	1.767600
C	8.570500	5.451100	4.878700
H	7.713600	4.533600	6.622400
H	9.159300	6.238300	2.965900
H	9.405900	5.889500	5.413000
B	5.084700	3.755700	2.651400

2′ *O*-coordinating $B(C_6F_5)_3$

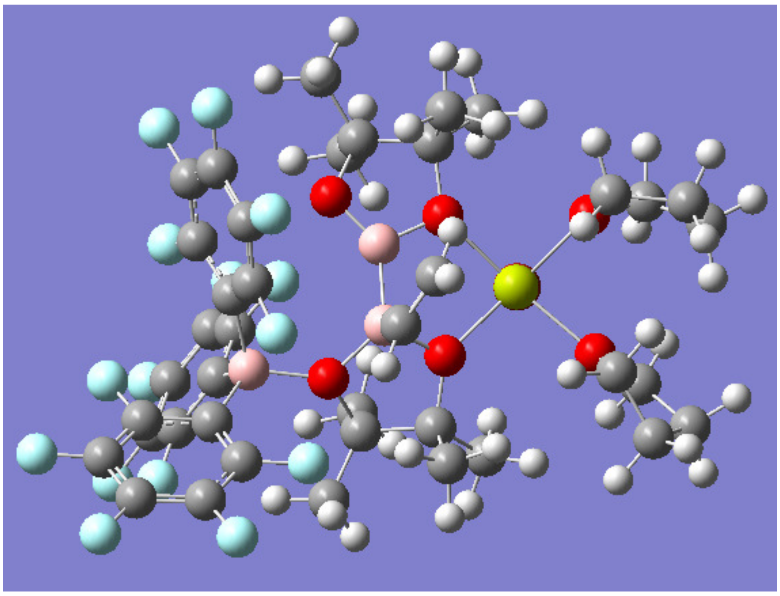

C	2.690200	6.973700	2.428800
C	3.744900	6.199900	3.277900
O	3.315800	6.986300	1.134100
O	4.155400	5.110900	2.332700
B	3.868100	5.603400	0.836100
C	3.172700	5.614900	4.557200
H	3.928000	5.049100	5.102300
H	2.856700	6.441100	5.199400
H	2.316300	4.973500	4.372100
C	4.907000	7.142000	3.602200
H	4.508200	8.049800	4.050300

H	5.576800	6.717100	4.341400
H	5.460200	7.423800	2.704000
C	1.329200	6.277000	2.391100
H	0.806600	6.454900	3.334000
H	0.728500	6.709500	1.590400
H	1.402400	5.208100	2.236200
C	2.460400	8.404100	2.904100
H	1.553100	8.788500	2.441300
H	2.310900	8.425500	3.986000
H	3.293900	9.056600	2.643200
C	2.716600	4.763700	0.072000
H	2.015700	4.142400	0.622600
C	7.139000	6.072200	-1.412000
C	6.048400	6.917000	-2.123400
B	5.195700	5.865600	-0.215600
O	5.036800	7.002600	-1.053400
O	6.343300	5.223500	-0.552100
C	7.963500	5.181200	-2.325000
H	8.494700	5.778400	-3.070200
H	8.700800	4.644900	-1.724600
H	7.342000	4.445100	-2.836000
C	5.416600	6.154900	-3.280400
H	4.498600	6.653200	-3.586600
H	6.096600	6.130600	-4.134100
H	5.176400	5.128100	-2.995700
C	6.493200	8.302200	-2.547100
H	7.373900	8.216300	-3.188900
H	5.702400	8.791200	-3.116800
H	6.740200	8.923200	-1.686100
C	8.044600	6.917400	-0.520400
H	8.628700	6.253400	0.117800
H	8.726800	7.521500	-1.121800
H	7.457100	7.579200	0.122400
C	2.515400	4.849600	-1.246800
H	1.692900	4.342500	-1.743700
H	3.167500	5.434400	-1.891600
C	5.638300	3.118400	1.410100
C	6.968900	2.952000	1.031000
C	4.740600	2.507800	0.532900
C	7.359400	2.387700	-0.173400
C	5.082000	1.966600	-0.693700
C	6.412500	1.925100	-1.062500
C	4.192800	2.640600	3.495500
C	4.929000	1.573200	4.004500

C	2.823900	2.433400	3.514600
C	4.373100	0.434000	4.555500
C	2.216900	1.310800	4.060000
C	2.995800	0.303500	4.592100
C	6.237600	4.432200	3.753100
C	6.411100	4.188000	5.109000
C	7.176000	5.308300	3.222100
C	7.445000	4.728800	5.863300
C	8.228900	5.861100	3.920900
C	8.369200	5.562900	5.265400
B	5.042200	3.858300	2.771900
Mg	3.699700	8.395000	-0.311200
O	1.911400	9.400800	0.033100
C	1.696200	10.737100	0.559900
C	0.654000	8.817700	-0.369200
C	0.180400	10.948700	0.528700
H	2.107100	10.762100	1.568500
H	2.245800	11.449700	-0.056000
C	-0.382600	9.522900	0.485800
H	0.489900	9.022300	-1.431400
H	0.728200	7.739600	-0.220200
H	-0.112900	11.491500	-0.372300
H	-0.161500	11.514700	1.393900
H	-1.377700	9.472800	0.046200
H	-0.422400	9.083600	1.484800
O	3.387700	9.063400	-2.283700
C	3.485500	10.468500	-2.595000
C	2.566000	8.468800	-3.310600
C	2.082900	10.838000	-3.048500
H	3.844600	10.984000	-1.706100
H	4.214000	10.592400	-3.403200
C	1.633000	9.584300	-3.821700
H	3.218300	8.120700	-4.113400
H	2.053200	7.614700	-2.867300
H	2.074300	11.737000	-3.662900
H	1.444800	11.009700	-2.180100
H	1.773100	9.719500	-4.893700
H	0.582200	9.351800	-3.652100
Br	5.239500	10.098500	0.679500
F	7.044300	5.707100	1.952500
F	9.083700	6.692200	3.323900
F	9.367400	6.080700	5.973200
F	7.551900	4.446300	7.162100
F	5.548900	3.432500	5.808400

F	6.271200	1.618100	3.974500
F	5.145400	-0.538000	5.042400
F	2.433500	-0.782900	5.116300
F	0.888100	1.188500	4.049100
F	1.980400	3.309900	2.945900
F	7.994000	3.342300	1.801000
F	8.654000	2.331300	-0.500000
F	6.775600	1.427700	-2.242600
F	4.145500	1.464100	-1.500700
F	3.446600	2.391900	0.863600

2′ *O*-coordinating BF_3

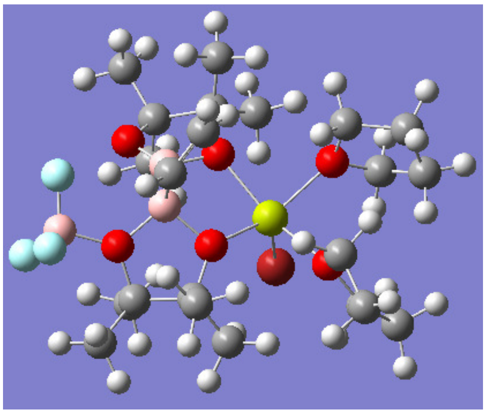

C	2.747200	6.983500	2.419600
C	3.891100	6.219600	3.139000
O	3.194700	6.984600	1.045800
O	4.122000	5.151700	2.176400
B	3.851200	5.626500	0.735300
C	3.487300	5.649100	4.490000
H	4.348800	5.189600	4.972600
H	3.126900	6.460100	5.126500
H	2.707000	4.897300	4.389300
C	5.172800	7.037000	3.249700
H	5.069300	7.807000	4.017100
H	5.987800	6.368700	3.528400
H	5.419800	7.526200	2.304600
C	1.426500	6.220600	2.517400
H	1.003400	6.317400	3.519300
H	0.712900	6.639600	1.806400
H	1.560900	5.161300	2.295000
C	2.577200	8.401700	2.935600
H	1.667100	8.838200	2.524700
H	2.477500	8.390200	4.023400
H	3.425200	9.033800	2.668200
C	2.853700	4.620000	-0.018300

H	2.356400	3.830000	0.549500
C	7.277900	6.471500	-1.092400
C	6.186900	7.125100	-1.986600
B	5.229100	6.021000	-0.179200
O	5.046900	7.123600	-1.057000
O	6.499900	5.554100	-0.277900
C	8.323600	5.677700	-1.854200
H	8.847600	6.321000	-2.565500
H	9.054900	5.278200	-1.149800
H	7.873900	4.844000	-2.392800
C	5.790100	6.233800	-3.156700
H	4.882900	6.627300	-3.615200
H	6.577800	6.218000	-3.912000
H	5.596600	5.210300	-2.827700
C	6.503900	8.531900	-2.452500
H	7.459000	8.528300	-2.984400
H	5.732400	8.878200	-3.141700
H	6.566300	9.228600	-1.616700
C	7.931400	7.472100	-0.145200
H	8.498900	6.922100	0.607100
H	8.613900	8.133100	-0.683500
H	7.179700	8.084400	0.359800
C	2.582900	4.693700	-1.323500
H	1.886100	4.025600	-1.821600
H	3.067600	5.433600	-1.959600
B	4.648200	3.761400	2.530500
Mg	3.657200	8.470500	-0.279200
O	1.829700	9.406300	0.051100
C	1.563000	10.742500	0.552100
C	0.591600	8.740900	-0.275900
C	0.039000	10.876500	0.563000
H	2.005400	10.813900	1.546000
H	2.056500	11.465100	-0.098900
C	-0.446900	9.423100	0.595600
H	0.376300	8.893500	-1.338800
H	0.732400	7.676600	-0.085300
H	-0.309100	11.363600	-0.350600
H	-0.305400	11.461300	1.414500
H	-1.455300	9.302700	0.202300
H	-0.418800	9.020500	1.610800
O	3.368500	9.087100	-2.249000
C	3.339900	10.476300	-2.628200
C	2.575800	8.357600	-3.210000
C	1.911600	10.694600	-3.097400

H	3.646000	11.061700	-1.762800
H	4.058600	10.627700	-3.440200
C	1.589800	9.373900	-3.819200
H	3.243800	7.948900	-3.970100
H	2.094100	7.535900	-2.678800
H	1.820500	11.562000	-3.749400
H	1.256100	10.839900	-2.236700
H	1.768300	9.468300	-4.890000
H	0.552900	9.069000	-3.680400
Br	5.079300	10.298900	0.678600
F	5.669400	3.912200	3.459400
F	3.594600	3.024800	3.057500
F	5.100600	3.218000	1.343700

[3][MgBr(THF)$_2$]

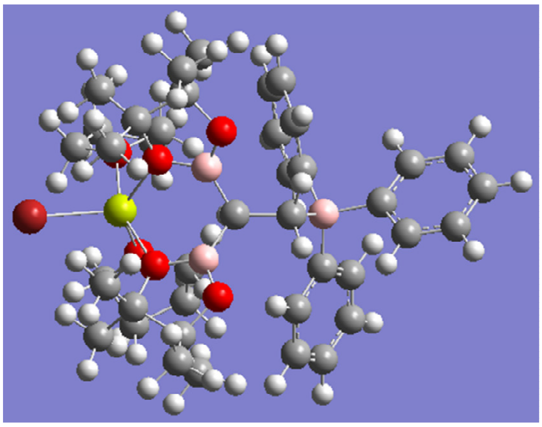

Br	4.062200	14.378700	6.569300
Mg	3.395100	12.121800	5.772300
O	5.179000	11.086300	5.299300
O	2.954000	11.989800	3.754500
O	6.419400	9.569000	4.133500
O	2.962700	11.023000	1.692900
O	3.214400	10.834700	7.374300
O	1.348800	12.403800	5.958100
C	3.829400	9.582700	3.711000
H	3.180500	9.478700	4.600100
C	7.299100	10.087900	5.164800
C	2.604300	5.894600	2.486500
C	6.589400	11.412300	5.592100
C	3.744000	5.241400	1.983500
H	4.724200	5.558400	2.331700
C	7.334800	9.047200	6.278800
H	6.349700	8.916800	6.734200
H	8.046100	9.328400	7.057000
H	7.641300	8.090400	5.851700

C	6.738700	11.747000	7.064200
H	6.259000	12.701700	7.283300
H	7.800200	11.831100	7.310000
H	6.298500	10.973800	7.694600
C	8.684500	10.279400	4.575300
H	9.100500	9.304100	4.318700
H	9.344600	10.752300	5.306500
H	8.655600	10.890900	3.674500
C	3.801900	8.252200	2.918600
H	4.819300	7.851600	2.841400
H	3.496800	8.463700	1.887600
C	3.420100	6.442800	4.933000
C	2.739200	13.091400	2.797800
C	1.322400	7.810500	3.874300
C	3.666700	4.198200	1.067100
H	4.573800	3.725900	0.703500
C	0.694200	8.566800	2.865500
H	1.216800	8.723700	1.924300
C	4.650100	6.812300	5.493700
H	5.250000	7.573200	5.003100
C	2.423500	3.754600	0.617400
H	2.354000	2.940600	-0.095600
C	0.585200	7.664800	5.060400
H	1.014200	7.110000	5.888600
C	-0.700700	8.184800	5.219500
H	-1.232800	8.027800	6.152600
C	-0.588600	9.092200	3.006800
H	-1.039700	9.647000	2.190100
C	2.750500	5.392000	5.589400
H	1.816000	5.030200	5.166900
C	1.371200	5.419400	2.017500
H	0.455100	5.880700	2.375100
C	2.276800	12.294700	1.537900
C	2.696600	12.915900	0.218800
H	3.781000	12.977800	0.135000
H	2.274700	13.919200	0.120700
H	2.320400	12.303400	-0.601800
C	4.090600	13.768200	2.614700
H	4.436400	14.140600	3.581200
H	4.007200	14.613300	1.929600
H	4.829600	13.067900	2.218200
C	6.966300	12.596500	4.714000
H	6.850300	12.354400	3.655200
H	8.003300	12.885300	4.894500

H	6.327600	13.446000	4.958900
C	1.713400	14.061100	3.351700
H	0.788000	13.557900	3.627200
H	1.496000	14.820000	2.595800
H	2.114300	14.560300	4.236200
C	5.160600	6.218900	6.651500
H	6.121500	6.534500	7.046200
C	-1.301700	8.896500	4.187700
H	-2.304500	9.294600	4.299500
C	1.275200	4.369500	1.102000
H	0.299400	4.032200	0.766800
C	3.237500	4.792400	6.745700
H	2.681000	3.988800	7.217200
C	3.388800	11.212600	8.764900
H	2.423000	11.552300	9.149900
H	4.106200	12.033000	8.801500
C	0.783600	11.995100	1.548700
H	0.569700	11.260800	0.769700
H	0.200200	12.896700	1.352700
H	0.478300	11.570700	2.507600
C	0.393700	11.382900	5.590800
H	0.920000	10.538100	5.140300
H	-0.299800	11.798100	4.850900
C	4.449600	5.212200	7.293100
H	4.840100	4.747300	8.191400
B	3.227800	10.827900	3.004900
B	5.170600	10.048900	4.346200
B	2.788100	7.114900	3.572900
C	3.077700	8.864100	8.673700
H	3.514400	7.868200	8.754600
H	2.037300	8.821600	9.005100
C	3.843600	9.935800	9.454300
H	4.921000	9.797500	9.333400
H	3.610600	9.943800	10.518200
C	-0.471600	12.473200	7.498100
H	-0.544100	12.465000	8.584900
H	-1.370600	12.945700	7.099400
C	0.781300	13.215600	7.018300
H	0.565500	14.204700	6.610600
H	1.539300	13.318700	7.794400
C	3.165600	9.386300	7.249800
H	4.083700	9.057700	6.759600
H	2.308300	9.118900	6.627000
C	-0.311300	11.072200	6.897900

H	0.332200	10.443300	7.520900
H	-1.260500	10.562500	6.746200

[2]$^{-}$

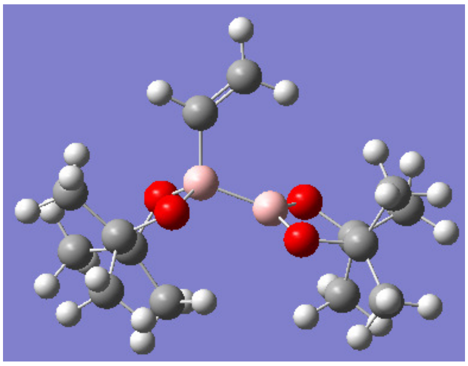

C	-1.927600	0.566600	0.236600
C	-2.203300	2.055000	-0.139300
O	-0.539400	0.443300	0.025200
O	-1.079200	2.713800	0.393000
C	1.013700	1.849300	1.554200
H	0.576600	1.425800	2.467800
C	2.240900	2.362200	1.676300
H	2.743500	2.809900	0.819200
H	2.796000	2.378900	2.612800
B	0.107300	1.799900	0.202800
C	2.416900	1.940700	-3.032500
C	2.045200	3.441800	-2.866500
B	1.044900	2.200400	-1.198100
O	1.513200	3.473600	-1.529500
O	1.454800	1.296500	-2.179900
C	-3.470700	2.641200	0.472900
H	-3.608200	3.666500	0.119100
H	-4.354100	2.060400	0.188900
H	-3.397400	2.664400	1.561100
C	-2.237300	2.242100	-1.664700
H	-3.163400	1.863300	-2.106800
H	-2.152400	3.309500	-1.884900
H	-1.392300	1.725700	-2.127600
C	-2.248500	0.301400	1.714600
H	-3.325100	0.263100	1.907000
H	-1.810900	-0.659300	1.997400
H	-1.804800	1.080800	2.336800
C	-2.665600	-0.449300	-0.630800
H	-2.450300	-1.460600	-0.275200
H	-3.749000	-0.296000	-0.589100
H	-2.338500	-0.379100	-1.668800

C	2.283000	1.399900	-4.447300
H	2.939300	1.945500	-5.131600
H	2.571100	0.346600	-4.462500
H	1.256700	1.479400	-4.805500
C	0.922700	3.873400	-3.808700
H	0.567800	4.858100	-3.499200
H	1.265100	3.933200	-4.844600
H	0.085200	3.174400	-3.750200
C	3.801500	1.614500	-2.473700
H	3.909700	0.529700	-2.423400
H	4.597700	2.018200	-3.103800
H	3.911300	2.014900	-1.463300
C	3.216000	4.406000	-2.968700
H	3.697300	4.323200	-3.947600
H	2.855900	5.429800	-2.848700
H	3.958400	4.211300	-2.194700

[2B]$^-$

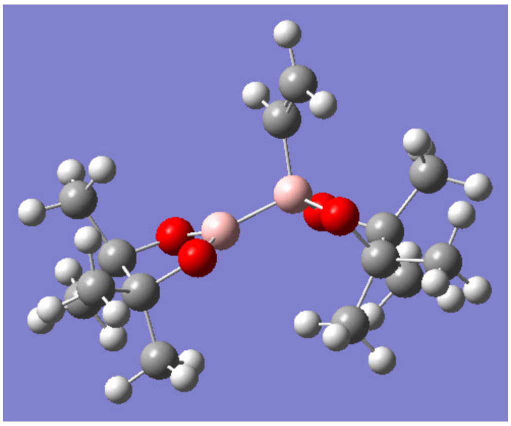

C	1.520200	1.591600	2.350200
C	2.391900	1.935000	1.100600
O	0.212700	1.860800	1.896500
O	1.620500	1.421300	0.040700
B	0.175900	1.608500	0.403200
C	3.767900	1.278900	1.087500
H	4.316600	1.594500	0.196000
H	4.352400	1.562400	1.968500
H	3.673800	0.192700	1.061200
C	2.544500	3.455500	0.937200
H	3.236000	3.881400	1.670200
H	2.924000	3.665300	-0.065900
H	1.572700	3.944000	1.046200
C	1.640900	0.106400	2.717700
H	2.604400	-0.130400	3.179500
H	0.845800	-0.139600	3.426500

H	1.509500	-0.506900	1.824500
C	1.814800	2.446600	3.578800
H	1.182800	2.124100	4.410800
H	2.860800	2.352000	3.888100
H	1.600400	3.496500	3.376900
C	-0.673200	0.281800	0.011800
H	-1.693700	0.203200	0.404800
C	-0.830700	4.737200	-1.845600
C	-2.136200	4.516100	-1.027500
B	-0.551900	2.959700	-0.403400
O	-1.686700	3.647900	0.029000
O	-0.113000	3.514000	-1.605000
C	-1.033800	4.915000	-3.342100
H	-1.663400	5.785900	-3.546300
H	-0.066100	5.071900	-3.823400
H	-1.496800	4.033500	-3.785000
C	-3.199300	3.753400	-1.817000
H	-3.996900	3.456400	-1.133300
H	-3.629900	4.367900	-2.611200
H	-2.773100	2.849900	-2.259200
C	-2.734700	5.777000	-0.424800
H	-2.995800	6.493200	-1.209500
H	-3.644600	5.522300	0.122500
H	-2.040500	6.251700	0.268400
C	0.023000	5.877500	-1.292600
H	1.005200	5.840800	-1.768300
H	-0.427700	6.852700	-1.491800
H	0.161400	5.766400	-0.214800
C	-0.275000	-0.691700	-0.812700
H	-0.899700	-1.538200	-1.093600
H	0.725800	-0.664600	-1.239600

TS

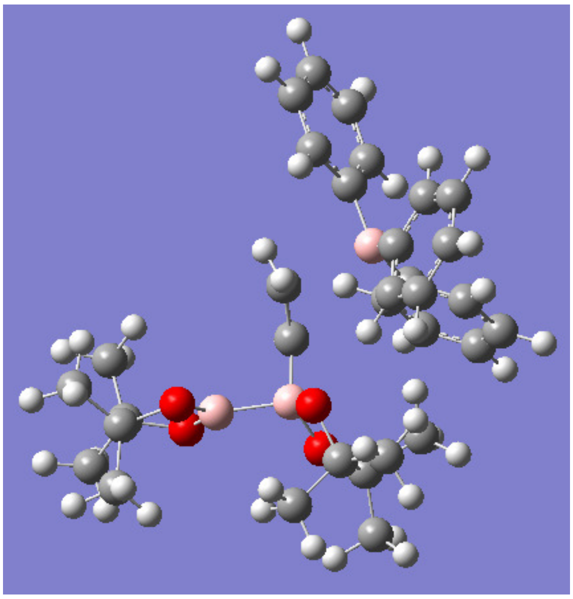

C	3.755500	6.548800	3.300100
C	4.625200	6.887200	2.046900
O	2.438300	6.756200	2.828100
O	3.857000	6.349800	0.987200
B	2.431800	6.496000	1.361700
C	6.003800	6.238700	2.041600
H	6.543700	6.531600	1.137000
H	6.590900	6.553400	2.909800
H	5.917300	5.151000	2.050900
C	4.759300	8.402200	1.847000
H	5.432400	8.855600	2.579900
H	5.152800	8.588700	0.844900
H	3.780500	8.882800	1.925700
C	3.905400	5.082300	3.710900
H	4.871900	4.875100	4.180800
H	3.110000	4.837300	4.419700
H	3.784800	4.431400	2.842400
C	4.006500	7.454300	4.500600
H	3.379600	7.134400	5.336900
H	5.052500	7.406900	4.820000
H	3.756600	8.489500	4.264600
C	1.452700	5.338500	0.840000
H	0.493400	5.330100	1.366900
C	1.263800	9.544500	-0.933100
C	-0.034300	9.257000	-0.124700
B	1.624500	7.812800	0.531400
O	0.461000	8.440400	0.956900
O	2.051000	8.364900	-0.668200
C	1.064000	9.686900	-2.432900

H	0.384100	10.514700	-2.653400
H	2.023800	9.895300	-2.910100
H	0.658400	8.772300	-2.864900
C	-1.033500	8.402800	-0.903200
H	-1.812000	8.064900	-0.216600
H	-1.501800	8.966500	-1.713300
H	-0.541400	7.522000	-1.323000
C	-0.720900	10.486400	0.445700
H	-1.021100	11.166100	-0.356900
H	-1.617600	10.183400	0.990000
H	-0.067200	11.021100	1.134400
C	2.045200	10.737800	-0.385900
H	3.034200	10.749700	-0.848200
H	1.542800	11.682400	-0.606900
H	2.173000	10.650900	0.695600
C	1.476500	4.598600	-0.307500
H	0.556700	4.237200	-0.753000
H	2.343400	4.631500	-0.961800
C	0.738700	1.772100	-0.345200
C	0.809600	1.800200	-1.749500
C	-0.276000	0.988600	0.217200
C	-0.089100	1.106300	-2.547300
H	1.592800	2.387400	-2.221700
C	-1.182100	0.280000	-0.573700
H	-0.358900	0.918900	1.296400
C	-1.095400	0.339800	-1.958200
H	-0.007600	1.156000	-3.627700
H	-1.954700	-0.319000	-0.103900
H	-1.799000	-0.206800	-2.575800
C	3.352700	2.314700	0.071600
C	3.733300	1.059900	-0.427200
C	4.367700	3.267700	0.247300
C	5.061700	0.759800	-0.727400
H	2.976400	0.295800	-0.577600
C	5.693400	2.979300	-0.063900
H	4.116800	4.262500	0.608700
C	6.048900	1.722000	-0.548500
H	5.322000	-0.223100	-1.105500
H	6.453200	3.742900	0.069700
H	7.082500	1.498300	-0.789100
C	1.606100	2.518400	2.125700
C	0.413200	2.955200	2.724400
C	2.572200	1.969000	2.978300
C	0.204700	2.875100	4.095600

H	-0.371600	3.368700	2.097300
C	2.369300	1.866600	4.353900
H	3.511000	1.619900	2.559100
C	1.186900	2.326300	4.919500
H	-0.723100	3.237700	4.524300
H	3.141500	1.438100	4.983600
H	1.027700	2.260000	5.989900
B	1.840300	2.528300	0.543500

[3]⁻

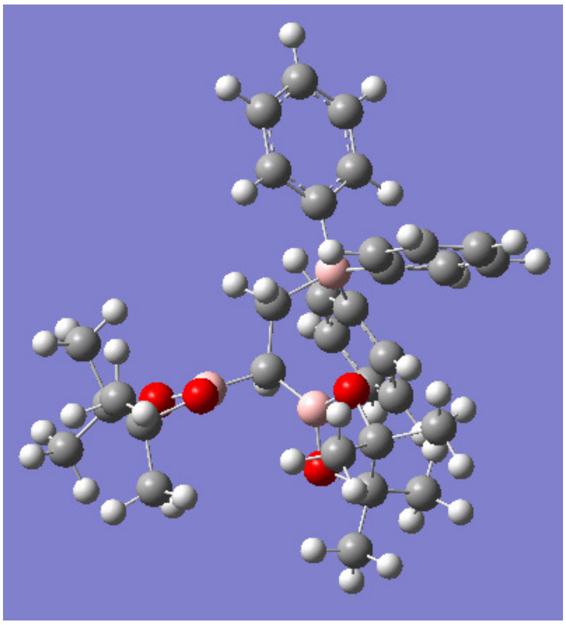

C	4.591700	6.507200	3.514600
C	5.251100	6.283300	2.111400
O	3.195000	6.641700	3.181900
O	4.173100	5.719300	1.342800
B	2.988100	6.066500	1.938900
C	6.411600	5.302400	2.100600
H	6.815900	5.232300	1.088600
H	7.209300	5.633000	2.771400
H	6.078200	4.307300	2.398200
C	5.638300	7.590100	1.423100
H	6.478100	8.078400	1.922500
H	5.922100	7.366400	0.393200
H	4.785000	8.271900	1.397400
C	4.724500	5.301000	4.440600
H	5.749900	5.184000	4.797900
H	4.067400	5.450300	5.299500
H	4.422200	4.382300	3.936600
C	5.048900	7.764900	4.237200
H	4.539200	7.834700	5.200300

H	6.126200	7.731300	4.420700
H	4.817200	8.659900	3.659900
C	1.576300	5.998600	1.290300
H	0.858300	5.697200	2.061100
C	1.587100	9.609100	0.180800
C	0.056500	9.319900	0.283500
B	1.240500	7.460200	0.865000
O	0.031200	8.093100	1.047800
O	2.146200	8.279700	0.215500
C	2.021600	10.301800	-1.098900
H	1.538500	11.278500	-1.188800
H	3.102400	10.454400	-1.079300
H	1.774900	9.704300	-1.975900
C	-0.576300	9.010900	-1.070800
H	-1.583200	8.624100	-0.905600
H	-0.642400	9.904500	-1.695200
H	-0.000200	8.249300	-1.601800
C	-0.744500	10.381100	1.017500
H	-0.649000	11.346700	0.513500
H	-1.799500	10.100400	1.025900
H	-0.408100	10.486900	2.048600
C	2.128700	10.349500	1.401900
H	3.219400	10.354400	1.353900
H	1.777900	11.383200	1.432500
H	1.827800	9.845700	2.323800
C	1.396900	5.085100	0.036300
H	0.350000	5.195100	-0.285400
H	1.991200	5.505800	-0.786100
C	0.879600	2.617100	-0.949800
C	0.486400	3.189000	-2.168700
C	0.609700	1.245400	-0.805700
C	-0.137700	2.452300	-3.176100
H	0.674600	4.245100	-2.342100
C	-0.015800	0.495000	-1.796700
H	0.897900	0.754500	0.121000
C	-0.396100	1.097800	-2.994700
H	-0.423700	2.938200	-4.103900
H	-0.208000	-0.561700	-1.638100
H	-0.885000	0.520700	-3.772000
C	3.268800	3.046700	-0.050300
C	3.895800	1.968500	0.594500
C	4.031700	3.674900	-1.048000
C	5.197200	1.562500	0.295900
H	3.348500	1.423200	1.358500

C	5.328600	3.283800	-1.364400
H	3.599000	4.504000	-1.599700
C	5.928400	2.225100	-0.684600
H	5.638800	0.724600	0.827000
H	5.876700	3.806500	-2.142500
H	6.940800	1.917400	-0.923600
C	1.170600	3.053500	1.704800
C	-0.180400	2.779800	1.970700
C	2.000100	3.162200	2.832300
C	-0.674300	2.632700	3.266000
H	-0.869600	2.689800	1.134700
C	1.526800	3.022100	4.135100
H	3.054600	3.370600	2.672200
C	0.178800	2.753900	4.360000
H	-1.728400	2.426600	3.424300
H	2.208600	3.124700	4.974100
H	-0.201200	2.642900	5.369600
B	1.694200	3.454400	0.209600

8. Crystallographic details of [3][MgBr(THF)$_2$]

Crystallographic data for **[3][MgBr(THF)$_2$]** was recorded on a Rigaku SuperNova X-ray diffractometer, at 150 K with Mo Kα radiation (mirror monochromator, λ = 0.71073). The CrysAlisPro[7] software package was used for data collection, cell refinement and data reduction. The CrysAlisPro software package was used for empirical absorption corrections, which were applied using spherical harmonics, implemented in SCALE3 ABSPACK scaling algorithm. All further data processing was undertaking within the Olex2 software.[8] The structure was solved using the ShelXT[9] structure solution program using Intrinsic Phasing. The structure was refined with the SHELXL[10] refinement package using Least Squares minimisation against F2. Non-hydrogen atoms were refined anisotropically. CCDC 1856184.

Identification code	[3][MgBr(THF)2]
Empirical formula	$C_{40}H_{58}B_3BrMgO_6$
Formula weight	771.51
Temperature/K	150.0(3)
Crystal system	monoclinic
Space group	$P2_1/c$
a/Å	17.499(3)
b/Å	13.2494(11)
c/Å	18.240(2)
α/°	90
β/°	107.952(16)
γ/°	90
Volume/Å^3	4023.1(10)
Z	4
ρ_{calc}g/cm^3	1.274
μ/mm^{-1}	1.081
F(000)	1632.0
Crystal size/mm^3	0.2 × 0.1 × 0.01
Radiation	MoKα (λ = 0.71073)
2Θ range for data collection/°	5.514 to 58.412
Index ranges	$-22 \leq h \leq 23$, $-15 \leq k \leq 17$, $-23 \leq l \leq 23$
Reflections collected	20620
Independent reflections	9297 [R_{int} = 0.1070, R_{sigma} = 0.1965]
Data/restraints/parameters	9297/0/468
Goodness-of-fit on F^2	0.960
Final R indexes [I>=2σ (I)]	R_1 = 0.0750, wR_2 = 0.1377
Final R indexes [all data]	R_1 = 0.1890, wR_2 = 0.1855
Largest diff. peak/hole / e Å^{-3}	0.62/-1.01

9. References

1 R. Robiette, Y. F. Guang, J. N. Harvey, V. Aggarwal, *Chem. Commun*. **2006**, *7*, 741-743.

2 F. Schodel, J. M. Breunig, V. Thiel, M. Bolte, M. Wagner, H.-W. Lerner, *Z. Naturforsch*. **2017**, *72*, 747-752.

3 A.-F. Pécharman, M. S. Hill, C. L. McMullin, M. F. Mahon, *Angew. Chem. Int. Ed*. **2017**, *56*, 16363-16366.

4 X. Liu, M. T. Deaton, F. Haeffner, J. P. Morken, *Angew. Chem. Int. Ed*. **2017**, *56*, 11485-11489.

5 Gaussian 09, Revision C1, Frisch, M. J.; Trucks, G. W.; Schlegel, H. B.; Scuseria, G. E.; Robb, M. A.; Cheeseman, J. R.; Scalmani, G.; Barone, V.; Mennucci, B.; Petersson, G. A.; Nakatsuji, H.; Caricato, M.; Li, X.; Hratchian, H. P.; Izmaylov, A. F.; Bloino, J.; Zheng, G.; Sonnenberg, J. L.; Hada, M.; Ehara, M.; Toyota, K.; Fukuda, R.; Hasegawa, J.; Ishida, M.; Nakajima, T.; Honda, Y.; Kitao, O.; Nakai, H.; Vreven, T.; Montgomery, Jr., J. A.; Peralta, J. E.; Ogliaro, F.; Bearpark, M.; Heyd, J. J.; Brothers, E.; Kudin, K. N.; Staroverov, V. N.; Kobayashi, R.; Normand, J.; Raghavachari, K.; Rendell, A.; Burant, J. C.; Iyengar, S. S.; Tomasi, J.; Cossi, M.; Rega, N.; Millam, J. M.; Klene, M.; Knox, J. E.; Cross, J. B.; Bakken, V.; Adamo, C.; Jaramillo, J.; Gomperts, R.; Stratmann, R. E.; Yazyev, O.; Austin, A. J.; Cammi, R.; Pomelli, C.; Ochterski, J. W.; Martin, R. L.; Morokuma, K.; Zakrzewski, V. G.; Voth, G. A.; Salvador, P.; Dannenberg, J. J.; Dapprich, S.; Daniels, A. D.; Farkas, Ö.; Foresman, J. B.; Ortiz, J. V.; Cioslowski, J.; Fox, D. J. Gaussian, Inc., Wallingford CT, 2009.

6 http://comp.chem.umn.edu/info/DFT.htm.

7 CrysAlisPro, Agil. Technol. Version 1.1 71.35.19 (release 27-10-2011 CrysAlis171.NET) (compiled Oct 27 2011,150211).

8 O. V. Dolomanov, L. J. Bourhis, R. J. Gildea, J. A. K. Howard and H. Puschmann, *J. Appl. Crystallogr.* 2009, **42**, 339.

9 G. M. Sheldrick, *Acta Cryst.* 2015, **A71**, 3.

10 G. M. Sheldrick, *Acta Cryst.* 2015, **C71**, 3.
